# Palatal rugae change shape following orthodontic treatment: a comparison between extraction and non-extraction borderline cases using fractal analysis and 3D superimposition

**DOI:** 10.1093/ejo/cjae070

**Published:** 2024-12-07

**Authors:** Miltiadis A Makrygiannakis, Dimitrios Konstantonis, Heleni Vastardis, Athanasios E Athanasiou, Demetrios J Halazonetis

**Affiliations:** Department of Orthodontics, School of Dentistry, National and Kapodistrian University of Athens, 2 Thivon str., Athens 11527, Greece; School of Dentistry, European University Cyprus, 6 Diogenous str., Engomi, Nicosia 2404, Cyprus; Department of Orthodontics, School of Dentistry, National and Kapodistrian University of Athens, 2 Thivon str., Athens 11527, Greece; Department of Orthodontics, School of Dentistry, National and Kapodistrian University of Athens, 2 Thivon str., Athens 11527, Greece; School of Dentistry, European University Cyprus, 6 Diogenous str., Engomi, Nicosia 2404, Cyprus; Hamdan Bin Mohammed College of Dental Medicine, Mohammed Bin Rashid University of Medicine and Health Sciences (MBRU), Dubai Healthcare City, P.O. Box 505055, Dubai, United Arab Emirates; Department of Orthodontics, School of Dentistry, National and Kapodistrian University of Athens, 2 Thivon str., Athens 11527, Greece

**Keywords:** palatal rugae, orthodontics, extractions, fractal analysis, 3D superimpostion

## Abstract

**Introduction:**

Palatal rugae are used as anatomical landmarks on the hard palate, in various clinical applications; in forensics, for insertion of mini-screws, and for superimposition. There is ambiguous evidence on whether they change during orthodontic treatment and to what extent. Therefore, we investigated changes in the shape, complexity, and area occupied by palatal rugae following orthodontic treatment with and without extractions.

**Materials and methods:**

Pre- and post-treatment plaster models of maxillae of 28 cases involving first premolar extractions (17 females and 11 males) and 33 non-extraction cases (19 females and 14 males) were scanned and analysed. All participants were selected from a parent sample via discriminant analysis and represent borderline cases. We applied mesh cropping, ball pivoting, distance mapping, contour cropping of rugae, best-fit superimposition, fractal dimension (FD) analysis, and creation of rugae’s convex hull area with Viewbox 4 software. The average distance between the closest points of the outlines of pre- and post-treatment palatal rugae (indicating shape change in the set of rugae), disparity in their pre- and post-treatment FDs (reflecting the complexity of their shapes), area occupied by rugae, arch depth, and size of palatal surface were then computed.

**Results:**

The medians of the average distance between pre- and post-treatment outlines after best-fit superimposition were 0.39 mm (interquartile range [IQR]: 0.34–0.51) and 0.27 mm (IQR: 0.22–0.34) mm for the extraction and non-extraction groups, respectively (*P* < 0.001). The median pre-treatment FDs were 1.497 (IQR: 1.481–1.521) for the extraction group and 1.481 (IQR: 1.456–1.509) for the non-extraction group, whereas their median post-treatment FDs were 1.502 (IQR: 1.472–1.532) and 1.489 (IQR: 1.469–1.501), respectively. The differences between pre- and post-treatment fractal dimensions were not found to be significant, neither within each group, nor across the groups. On the other hand, the surface area occupied by rugae showed a median increase of 14.7 mm^2^ (IQR: 0.0–46.5) (*P* = 0.003) following non-extraction treatment only.

**Conclusion:**

Palatal rugae change shape during orthodontic treatment, but their shape complexity, as measured by fractal dimensions, remains unaltered. Extraction treatment exerts a more pronounced effect in shape change compared to treatment without extractions. Nevertheless, non-extraction orthodontic treatment increases the surface on which rugae lie, as measured by means of the convex hull. Although the alterations may appear minor, it is necessary to exercise caution and prudence when employing rugae for superimposition and forensic dentistry purposes.

## Introduction

The palatal rugae are irregular ridges, located on the mucosa of the hard palate distal to the incisive papilla and lateral to the mid-palatal suture [1, 2]. The reason for their existence has not been fully clarified [3]. However, embryonic rugae have long been thought to contribute to the elevation of the palatal shelves, from the sides of the tongue to a horizontal position above the tongue’s dorsum. In fact, it has also been postulated that these anatomical structures provide rigidity to the palatal shelves [4] and the cellular reorganization within them might participate in the exertion of forces required for the horizontal alignment of the palatal shelves before subsequent fusion along the midline [4–7].

In terms of function, the palatal rugae’s role is considered to be complementary to masticatory activity as they take part in sensing, clenching, and crushing of food [4, 8]. From a clinical and research standpoint, rugae are used as reference structures for superimposition [9]. They can also serve as anatomical guides for the placement of mini-screws in the palatal area [10]. Furthermore, they may be used for post-mortem identification in forensic odontology, when other methods (fingerprints, DNA, dental records, etc.) are ineffective [11–13].

Regarding the assessment of rugae’s morphology, various methods have been utilised, with a significant number of them allowing a qualitative description of the rugae’s traits [1, 14–18]. Their description focuses mainly on the position, length (categorizing rugae as primary, secondary, or fragmentary), shape (curved/wavy/straight/circular), direction, and unification of rugae. Variations among the methods’ indices could lead to different outcomes among studies. To overcome this challenge and obtain quantitative comparisons, a recent method to delineate rugae and assess their complexity was developed [19]. This method produces consistent quantitative results and requires minimal human intervention, rendering it an objective and applicable tool in research [19].

When alterations of palatal rugae need to be assessed, a significant restriction is the lack of stable structures for superimposition. So far, the impact of orthodontic interventions on rugae morphology has been evaluated by several studies [20–28]. Their outcomes have mainly focussed on alterations in qualitative shape characteristics, in the length of individual rugae, and in the positions of their most lateral and medial points, with conflicting results [20–24, 26]. Three-dimensional superimposition was used by a recent study that assessed the stability of rugae after orthodontic treatment [28]. Among the studies that compared the effect of extraction and non-extraction treatment on palatal rugae [21, 24–28], none reported on whether the two groups had similar characteristics at the outset.

Our objective was to examine whether any changes occur in the shape, complexity, and area occupied by palatal rugae, following orthodontic treatment with upper first premolar extractions or without extractions. We used a recently published method that provides a comprehensive evaluation of rugae’s complexity with minimum user intervention and high reliability [19]. In addition, we ensured that the extraction and non-extraction groups were similar, by drawing them from a larger parent sample via discriminant analysis [29–31].

## Methods and materials

### Ethical approval

All methods were conducted in accordance with relevant guidelines and regulations. This study is part of a broader scientific project which has been approved by the Research Ethics Committee of the Dental School of the National and Kapodistrian University of Athens (Protocol number: 495/ 01.03.2022). For this specific part of the project, the Research Ethics Committee waived the need for informed patient consent, since the sample consisted of archived pre- and post-treatment dental models.

### Sample

**Figure 1.**
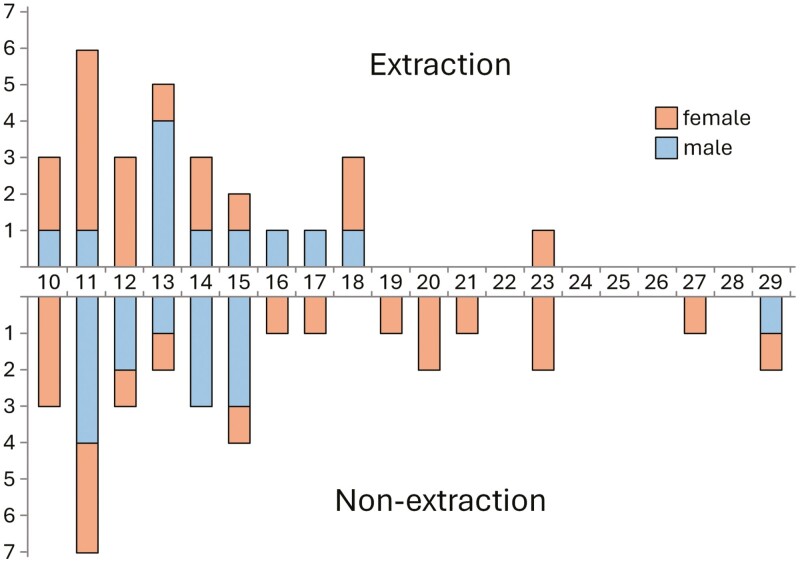
Age and sex distribution in both groups.

Pre- and post-treatment maxillary plaster models (type IV plaster) of patients who had undergone orthodontic treatment were retrieved and scanned with a 3D structured white light scanner (Identica, Medit Co. Ltd, Seoul, South Korea). To avoid susceptibility bias, the casts were sourced from a larger sample to which discriminant analysis had been applied and cases with similar malocclusions, assessed as borderline for extraction treatment, had been identified [29–31]. Twenty-six cephalometric (SNA, SNB, ANB, U1-SN, U1-NA, NSGn, FMIA, IMPA, FMA, L1-NB, U1-L1, SN-PP, SN-OP, Z angle, PNS-A, U1-NA, L1-NB, L1-APg, Pg-NB, Wits, N-Me, N-ANS, ANS-Me, LL-E-plane, S-Go, S-Ar), six dental cast variables (overbite, overjet, maxillary and mandibular crowding, upper and lower midline deviation) along with sex and age were included in the discriminant analysis, encompassing the majority of morphological characteristics which orthodontists might consider in treatment planning [29–31]. Each patient was given a discriminant score, which indicated the likelihood of being classified as either extraction or non-extraction. A more negative score indicated a higher likelihood of treatment involving extractions, while a more positive score indicated a higher likelihood of non-extraction treatment. Patients whose scores were closest to ‘0’ and thus could not be clearly classified into either group were considered the borderline cases included in this study [31]. This sample had been collected from the archives of the Postgraduate Orthodontic Clinic of the National and Kapodistrian University of Athens and five private orthodontic clinics located in Athens, Greece. Twenty-eight cases (17 females, 11 males; mean age 13.5 years) treated with first premolar extractions and 33 cases (19 females, 14 males; mean age 15.6 years) treated without extractions (Fig. 1, Supplementary Table 1) were retrieved.

All included patients were Caucasians and presented with a full complement of permanent teeth (without considering third molars) and a Class I malocclusion, as per patients’ dental casts and intraoral photographs. Individuals with facial deformities, clefts, and history of previous orthodontic or orthognathic surgery treatment were excluded. All patients had been treated with buccally placed fixed edgewise preadjusted orthodontic appliances in both arches. Extra-oral appliances and temporary anchorage devices had not been used.

In the extraction group, after the first premolar extractions, crowding was initially alleviated by canine retraction, and then the remaining space was addressed with reciprocal anchorage. The extraction decisions had been based on orthodontic reasons only and not on the presence of decay or compromised periodontal status. In non-extraction cases, crowding was addressed by the expansion of the arches and proclination of the incisors.

### Analysis of the 3D models

**Figure 2.**
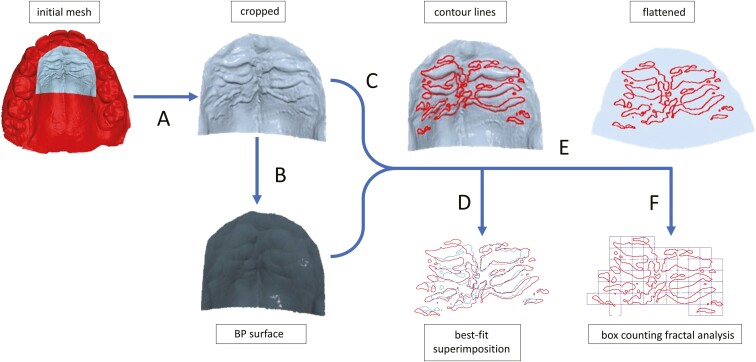
Step-by-step presentation of the methodology: (A) Cropping of the mesh, (B) Creation of Ball Pivoting (BP) surface, (C) Delineation of rugae’s contour lines, (D) Best-fit superimposition between pre- and post-treatment rugae, (E) Flattening of the surface, (F) Box-counting fractal dimension analysis.

**Figure 3.**
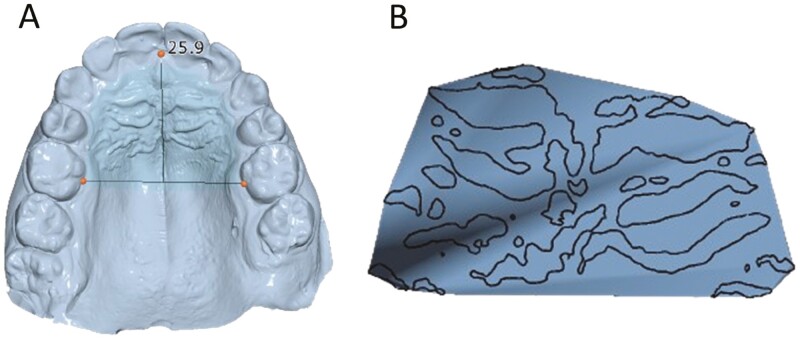
(A) Arch depth measured from the contact point of central incisors to the line connecting first molars' palatal grooves, (B) Convex hull of rugae’s area on flattened surface.

The digitized 3D maxillary models were imported, processed, and analysed with Viewbox 4 software (version 4.1.2.1, dHAL Software, Kifissia, Greece). The processing and analysis procedures were based on a recently published method, used for delineating palatal rugae and assessing their complexity [19]. Nevertheless, some additional methodological steps (steps 7, 8, and 11) were applied to calculate the average distance of the closest points of the contour lines of pre- and post-treatment rugae. The processing and analysis were carried out by one author (MAM) and cross-checked by another author (DJH). The procedure for analysis of both pre- and post-treatment models was the following (Figures 2 and 3):

1. The digital models were oriented to match the transverse, sagittal, and vertical directions with the software’s X-, Y-, and Z-axes, respectively. The X- and Y-axes were aligned with the occlusal plane. This alignment is optional and does not influence the results.
2. The area including the rugae was determined by a line connecting the cervical margins of the first molars at the palatal grooves and extending to the most palatal points of the teeth located mesial to them. The mesh outside this selected area was then removed.
3. A smoothened surface (BP surface) without terrain anomalies was created using the ball pivoting algorithm (BPA) [32, 33].
4. A distance map was created; heights between the original mesh and the BP surface were measured for each vertex, with terrain features identified as height differences between the two surfaces.
5. Contour lines of the rugae were delineated on the mesh based on vertex distances from the BP surface. Each contour line consisted of discrete points (DP) connecting the edges of the triangular mesh surface.
6. Contour lines that represented artefacts (e.g. bubbles, elongations) or adjacent anatomical structures, such as the incisive papilla and palatal gingivae, were removed. In addition, if rugae contour lines were unclear in either the pre- or post-treatment models, they were excluded from both.
7. A best-fit superimposition (iterative closest point [ICP] algorithm) of the rugae between pre- and post-treatment models was performed based on the discrete points forming their contour lines, to evaluate whether rugae coincided before and after treatment and to what extent.
8. The average distance between the closest discrete points of the contour lines of the pre- and post-treatment rugae was calculated.
9. Surface Flattening [34].
10. Box-counting fractal dimension (FD) analysis was used to assess the complexity of rugae’s shape—a geometrical configuration found to be too irregular to be described by means of Euclidean Geometry. This method, which is performed automatically by Viewbox 4 software, involves placing progressively smaller grids (or ‘boxes’) over the image of the rugae contour lines and counting the number of boxes that contain significant details, such as the discrete points of the palatal rugae contours [35]. Further technical details can be found in the publication of the methodology [19].
11. The discrete points of the rugae contours on the flattened surface were converted into point clouds, and their convex hull was created to calculate the surface area covered by the rugae.

All of the above steps were automatically performed using software without any manual intervention, except for steps 2 and 6, which required user input.

Apart from the average distance of the closest points of pre- and post-treatment rugae’s contour lines and pre- and post-treatment FDs, other outcomes, namely, arch depth, palatal surface, and size of the surface on which rugae extended were also measured for every patient. Arch depth was determined by means of a customized template in Viewbox software as the shortest distance from the central incisors’ contact point to a line connecting the palatal grooves of the first molars. The palatal surface area was defined and measured as the one established in step 2.

Finally, the surface on which palatal rugae extended was computed as the surface of the convex hull of the discrete points of rugae’s contours on the flattened surface. A convex hull of a set of points is the smallest convex polygon that encompasses all the points within the set [36].

### Statistical analysis

Inter- and intra-observer reliability of the method has already been presented in a previous publication [19]. A Bland-Altman analysis showed that the 95% limits of agreement for FD ranged from −0.012 to 0.011 and −0.004 to 0.004 for inter- and intra-examiner repeatability, respectively [19]. The operators [MAMand DJH] who performed the measurements were the same for both the previous and present study and had already been calibrated in the methodology.

The distances between the closest discrete points of the contour lines of the pre- and post-treatment rugae were computed by software-based best-fit superimposition and no user interaction was necessary.

We included all the patients who fulfilled the selection criteria. Based on an α probability of 0.05 and a power of 0.8, we computed that a sample size of 28 and 32 patients in each group would allow for the detection of an effect size of 0.75.

As not all sample data followed a normal distribution, it was decided to apply non-parametric tests for all outcomes; namely Mann–Whitney *U* tests for unpaired comparisons between the extraction and non-extraction groups and Wilcoxon signed-rank test for paired pre- and post-treatment outcomes within each group.

## Results

### FD changes

Pre-treatment FDs of the palatal rugae between the two groups (non-extraction: median of 1.481, interquartile range [IQR]: 1.456–1.509 and extraction: median of 1.497, IQR: 1.481–1.521) were not found to be significantly different (*P* = 0.058). The median post-treatment FDs for the non-extraction group and extraction groups were 1.489 (IQR: 1.469–1.501) and 1.502 (1.472–1.532), respectively and the differences between pre- and post-treatment FDs were not found to be significant (*P* > 0.05) (Table 1, Supplementary Table 2).

**Table 1.** Summary results.

Outcome	Non-extraction group		*P*-value^*^	Extraction group		*P*-value^*^	Non-extraction difference Median (IQR)	Extraction difference Median (IQR)	*P*-value** (extraction vs. non-extraction)
	Pre-tx Median (IQR)	Post-tx Median (IQR)		Pre-tx Median (IQR)	Post-tx Median (IQR)				
**FD**	1.481 (1.456–1.509)	1.489 (1.469–1.501)	0.741	1.497 (1.481–1.521)	1.502 (1.472–1.532)	0.891	−0.001 (−0.023 to 0.016)	0.000 (−0.018 to 0.030)	0.891
**Arch depth** **(mm)**	31.9 (30.3–33.3)	31.3 (30.4–32.7)	0.036	32.2 (29.5–33.7)	25.4 (23.9–27.0)	<0.001	−0.6 (−2.6 to 0.4)	−6.7 (−7.7 to −5.1)	<0.001
**Palatal surface** **(mm** ^**2**^)	1101.2 (1014.7–1161.0)	1161.4 (1048.7–1252.8)	0.003	1099.9 (1006.9–1241.9)	989.9 (921.2–1039.4)	<0.001	62.0 (2.3–96.1)	−140.4 (−217.6 to −83.7)	<0.001
**Rugae convex hull area** **(mm** ^**2**^)	414.5 (341.8–464.8)	439.9 (375.0–479.2)	0.003	378.1 (312.5–495.4)	384.5 (320.9–482.5)	0.425	14.7 (0.0–46.5)	−4.0 (−28.3 to 19.8)	0.019
							**Non-extraction** **Median (IQR)**	**Extraction** **Median (IQR)**	
**Average distance** **(mm)**							0.27 (0.22–0.34)	0.39 (0.34–0.51)	<0.001

FD: fractal dimension; IQR: interquartile range; Pre-tx: pre-treatment; Post-tx: post-treatment.

^*^Wilcoxon Signed rank test.

^**^Mann–Whitney *U* test.

### Average distance between the closest points of pre- and post-treatment rugae outlines

After best-fit superimposition, the medians of average distances between pre- and post-treatment outlines were found to be 0.39 mm (IQR: 0.34–0.51) and 0.27 mm (IQR: 0.22–0.34) for the extraction and non-extraction groups, respectively, revealing a statistically significant difference between them (*P* <0.001) (Table 1, Supplementary Table 3). The superimpositions between pre- and post-treatment rugae of all patients are available in Supplementary Figs 1 and 2.

### Arch depth

The arch depth in both extraction and non-extraction cases decreased significantly by a median of −6.7 mm (IQR: −7.7 to −5.1) (*P* < 0.001) and a median of −0.6 mm (IQR: −2.6 to 0.4) (*P* = 0.036), respectively, and, as expected, the disparities between the two groups were also significantly different (*P* < 0.001) (Table 1, Supplementary Table 4).

### Palatal surface

After orthodontic treatment, there was a notable reduction (median −140.4 mm^2^, IQR: −217.6 to −83.7) (*P* < 0.001) in the palatal surface of extraction cases, but an increase (median: 62.0 mm^2^, IQR: 2.3–96.1) (*P* = 0.003) was observed in the non-extraction group. The difference between the two groups was statistically significant (*P* < 0.001) (Table 1, Supplementary Table 5).

### Convex hull of the area occupied by rugae

The convex hull of the area occupied by rugae did not change significantly in extraction cases, but increased in non-extraction cases, by a median 14.7 mm^2^ (IQR: 0.0 to 46.5) (*P* = 0.003) (Table 1, Supplementary Table 6).

## Discussion

This study investigated whether the shape and fractal dimension (complexity) [37] of palatal rugae undergo changes in individuals who underwent orthodontic treatment, with or without extractions. Various fractal analyses have been described in the literature. Among them, the box-counting method has been employed to calculate the area of irregular cartographic features [38, 39], irregularity being a characteristic shared with palatal rugae contours. This analysis has also been widely used in various areas of dental research [40–43] and was employed in a new method for the assessment of the shape complexity of the palatal rugae [19].

Although palatal rugae have been considered stable and suitable for post-mortem identification [44] and superimposition [45], an increasing body of recent research casts doubt on this assertion [21, 22, 46]. Also, it has been demonstrated that rugae undergo positional changes during orthodontic treatment of growing patients, especially in the vertical dimension [47]. Nevertheless, whether changes in the overall shape, complexity and area of the entire set of palatal rugae occur during orthodontic treatment, and whether differences exist between extraction and non-extraction treatment, had not been examined.

### Changes in shape between pre- and post-treatment rugae

Our results show that pre- and post-treatment rugae do not fully coincide after best-fit superimposition in both extraction and non-extraction groups, respectively. These findings may be attributed to the soft tissue stretching or folding of the mucosa and, specifically, of the area on which palatal rugae lie, as it adapts to the adjustments of hard tissue due to tooth movement and remaining growth [22]. Also, it is logical to assume that in cases involving extractions, teeth are required to cover a longer distance. Thus, a relatively greater movement of teeth could be expected to result in an overall greater shape change of rugae in extraction compared to non-extraction cases.

An additional explanation could be provided by a recent paper which demonstrated alterations in overall palatal shape after orthodontic treatment for both non-extraction and extraction cases, with the latter showing more substantial changes [48]. Its aim was to assess changes in palatal shape following orthodontic treatment in borderline cases of Class I relationship patients, comparing those who underwent premolar extractions with those who did not. After applying discriminant analysis, 30 non-extraction and 23 extraction patients were selected, and their palatal shapes were analysed with superimposition and principal component analysis. Results showed no sexual dimorphism in palatal shape, with significant differences found mainly in the extraction group, which had a pronounced decrease in palatal length and an increased palatal height. On the other hand, the non-extraction group exhibited increased palatal width. These alterations could contribute to changes in rugae’s shape and may explain the greater distance observed in the extraction group and the increase of the area occupied by rugae in the non-extraction group.

### Changes in the area occupied by the rugae

We also sought to investigate whether changes occurred not only in the shape but also in the area occupied by the rugae. Consequently, we evaluated the surface on which rugae lie and its alterations following orthodontic treatment. This assessment was conducted using the convex hull method on the flattened surface of the rugae. The convex hull, as explained above, represents the smallest convex polygon enclosing all points within a set [36]. The measurements revealed an increase in the area occupied by rugae only in non-extraction cases, with no significant changes observed in extraction cases. This suggests that in non-extraction cases, the external points of rugae’s contours—forming the convex hull area—move in an expansive direction, while those in extraction cases remain relatively stable. The expansion of the external points of rugae in non-extraction cases could be the result of a potential stretching of the mucosa following the observed increase in palatal surfaces in non-extraction cases.

### Changes in fractal dimensions

Regarding the disparity in fractal dimensions, no remarkable change was detected between pre- and post-treatment rugae within each group and across the groups. Despite the fact that the shape of rugae does change following orthodontic treatment, these alterations are not sufficient to yield a significant distinction in the complexity of rugae before and after treatment and in their difference across the groups. A reasonable explanation could be that the sets of palatal rugae do not become more or less convoluted because their deformation is overall global and not regional.

### Impact on rugae’s employment in various tasks

From a clinical perspective, it becomes evident that rugae’s shape changes after orthodontic treatment, irrespective of whether extractions have been performed. This suggests that palatal rugae are not absolutely stable and, therefore, cannot be considered entirely reliable anatomical landmarks for superimposition. Furthermore, in cases where rugae are utilized for the identification of an individual, pre- and post-mortem rugae would not be anticipated to be in complete agreement, especially when orthodontic treatment has taken place since the acquisition of the last available records.

### Methods of previous studies

Previous studies tried to assess the effect of orthodontic treatment and in some instances compared extraction to non-extraction cases [20–28, 49]. The methodology of some of these studies included the delineation of rugae with a pencil under adequate light and magnification. Rugae smaller than 2 mm were not taken into consideration for further analysis. Rugae’s lengths, qualitative traits of their shape, and distance of their most medial and lateral points from the median palatal plane were the main outcomes assessed. There was controversy with regard to stability and shape changes of palatal rugae and it was concluded that there may be changes in rugae’s lengths and positions of their most medial and lateral points; two of the studies advised caution against using rugae as identification markers in forensic odontology [24, 25].

Moreover, a recent paper by Zhao *et al*. assessed rugae’s stability after orthodontic treatment with 3-D superimposition [28]. In that study, palatal rugae were manually extracted and converted into 3D point clouds. In contrast, the depiction of rugae in our research was based on distance mapping and was achieved by an automated process of the software, hence minimizing human intervention and subjective interpretation. Furthermore, Zhao *et al.* employed a correntropy-based ICP registration algorithm for superimposition, with the minimum point-to-point root mean square (RMS) distances computed to evaluate the variation in palatal rugae scans within the same individual and across different individuals [28]. We also performed superimposition with an ICP algorithm. However, we assessed the distances between sets of rugae within the same individuals, but not across different ones, and we did not investigate rugae individually. It is worth mentioning that our results also show that extraction treatment had a more notable effect on rugae.

3D superimposition was applied by another study, as well [50]. The goal of that study was to examine whether rugae remained stable following slow maxillary expansion [50]. The palatal areas occupied by rugae were manually selected from pre- and post-treatment casts; however, the exact manner these surfaces were isolated was not clear. Subsequently, superimposition was automatically performed in order to achieve the best match between the surfaces of the two models. Each model was superimposed to another cast of the same individual but also to casts of other participants. Their results showed that the RMS of point-to-point distances was smaller when superimposition had been performed on casts of the same person. The study did not present any differences between the RMS values of the test group and the RMS values of the untreated control group. On the other hand, in our study, the area of rugae was cropped following specific guidelines, and the rugae, per se—not the palate—were used for best-fit superimposition. Our outcomes reflected the average distance between pre- and post-treatment rugae, the overall change of complexity of their shape, and changes in palatal surface-related measurements, and not the RMS values of point-to-point distances. Also, we superimposed pre- and post-treatment rugae of the same individuals only.

### Strengths and limitations

As far as the strengths of our investigation are concerned, one of its significant assets is the nature of its sample. The included patients were borderline cases selected from a parent sample via discriminant analysis to minimize susceptibility bias [29–31]. The concept of selecting a sample with these traits was to observe the very effect of extractions on orthodontic treatment. Another strength is the implementation of a recently published method whose reliability and objectivity have been demonstrated. It provides a comprehensive evaluation of the complexity of all rugae (regardless of their size), with fractal analysis and a complete set of information about their contours, with distance mapping. Minimal user intervention renders the results repeatable and objective. In addition, average distance, fractal dimensions, and palatal surface-related measurements are quantitative variables and this makes them comparable.

Regarding limitations, it is crucial to note that the present study had growing patients in its sample and did not have an untreated control group. In terms of growth, apart from bone apposition at the maxillary tuberosity, which is not close to the examined site, the usual growth pattern of the maxilla involves osseous apposition on the oral side of the palate and resorption on the nasal side [51]. Pazera and Gkantidis (2021) found an agreement between their detected forward displacement of all rugae and papilla points and downward and forward movement of the maxilla, led by both primary and secondary translation [47, 51]. As far as the absence of a control group is concerned, it may be speculated that the changes found in this investigation cannot be solely attributed to orthodontic treatment. Therefore, a clear cause and effect relationship between orthodontic treatment and rugae’s shape change cannot be established in the present study.

Regarding digitization of scanned plaster models, a recent publication concluded that both traditional and digital impression techniques are clinically acceptable and can be performed interchangeably for assessing superimpositions and conducting evaluations of palatal rugae during treatment and post-treatment [52]. In this study, Viewbox software was used to address errors related to the presence of artefacts and dimensional changes of the impression materials and gypsum [53, 54]. In fact, all unwanted details on the casts, such as rugae’s elongations and bubbles, were digitally removed. In case an area containing rugae was not clearly depicted in either the pre- or post-treatment cast, then it would be cropped from both models.

### Recommendations for future research

Taking into consideration all the above-mentioned methodological aspects, we think that the present study may be a step towards the direction of revealing the pattern with which rugae change following orthodontic treatment. Concerning recommendations for future investigations on palatal rugae, it would be interesting to observe the effect of other orthodontic treatment modalities, and, if possible, to implement prospective study designs to avoid pertinent limitations. Furthermore, the presence of a control group would answer whether rugae changes occur without any intervention. Also, the study of changes and stability of each ruga individually—not the entire set—and the use of stable references, such as a mini-screws, could prove to be helpful in a further exploration in this field. Finally, intraoral scans could be preferred over dental casts or digitized models.

## Conclusions

Palatal rugae change shape during orthodontic treatment, but their shape complexity, as measured by fractal dimension analysis, remains unaltered. Regarding shape alterations, treatment involving extractions exhibits a more noticeable effect compared to non-extraction treatment. In contrast, non-extraction treatment appears to increase the surface on which rugae lie, as measured by means of the convex hull method. Despite the subtle nature of these changes, caution and prudence is advised when employing rugae for tasks such as superimposition and forensic dentistry.

## Supplementary Material

cjae070_suppl_Supplementary_Tables_1-6

cjae070_suppl_Supplementary_Figures_1-2

## Data Availability

The datasets generated and/or analysed during the current study are available from the corresponding author on reasonable request.
