# Supplementary material for: Palatal rugae change shape following orthodontic treatment: a comparison between extraction and non-extraction borderline cases using fractal analysis and 3D superimposition
Source: Eur J Orthod. 2024 Dec 7;47(1):cjae070. doi: 10.1093/ejo/cjae070 (PMC11631060; doi:10.1093/ejo/cjae070)
Supplement: cjae070_suppl_Supplementary_Tables_1-6 [file cjae070_suppl_supplementary_tables_1-6.docx]

**Supplementary Table 1**. Age (in years) and sex (F: female; M: male) of included patients.

| **Non-extraction group** | | | **Extraction group** | | |
| --- | --- | --- | --- | --- | --- |
| **Patient ID** | **Age** | **Sex** | **Patient ID** | **Age** | **Sex** |
| Nonex01 | 11 | F | Ex01 | 18 | F |
| Nonex02 | 17 | F | Ex02 | 15 | M |
| Nonex03 | 20 | F | Ex03 | 14 | F |
| Nonex04 | 15 | M | Ex04 | 13 | M |
| Nonex05 | 29 | F | Ex05 | 12 | F |
| Nonex06 | 10 | F | Ex06 | 18 | F |
| Nonex07 | 14 | M | Ex07 | 14 | F |
| Nonex08 | 23 | F | Ex08 | 13 | F |
| Nonex09 | 10 | F | Ex09 | 15 | F |
| Nonex10 | 11 | F | Ex10 | 11 | M |
| Nonex11 | 15 | F | Ex11 | 11 | F |
| Nonex12 | 20 | F | Ex12 | 14 | M |
| Nonex13 | 12 | F | Ex13 | 17 | M |
| Nonex14 | 12 | M | Ex14 | 13 | M |
| Nonex15 | 11 | M | Ex15 | 23 | F |
| Nonex16 | 14 | M | Ex16 | 13 | M |
| Nonex17 | 15 | M | Ex17 | 10 | F |
| Nonex18 | 13 | F | Ex18 | 11 | F |
| Nonex19 | 12 | M | Ex19 | 11 | F |
| Nonex20 | 21 | F | Ex20 | 10 | F |
| Nonex21 | 11 | M | Ex21 | 12 | F |
| Nonex22 | 10 | F | Ex22 | 12 | F |
| Nonex23 | 11 | M | Ex23 | 16 | M |
| Nonex24 | 11 | F | Ex24 | 18 | M |
| Nonex25 | 19 | F | Ex25 | 11 | F |
| Nonex26 | 16 | F | Ex26 | 10 | M |
| Nonex27 | 11 | M | Ex27 | 13 | M |
| Nonex28 | 13 | M | Ex28 | 11 | F |
| Nonex29 | 29 | M |  |  |  |
| Nonex30 | 14 | M |  |  |  |
| Nonex31 | 23 | F |  |  |  |
| Nonex32 | 15 | M |  |  |  |
| Nonex33 | 27 | F |  |  |  |
| **Average** | 15.6 |  | **Average** | 13.5 |  |
| **SD** | 5.5 |  | **SD** | 3.1 |  |

SD: standard deviation.

**Supplementary Table 2**. Fractal dimensions (FDs) of pre- and post-treatment palatal rugae.

| **Non-extraction group** | | | | **Extraction group** | | | |
| --- | --- | --- | --- | --- | --- | --- | --- |
| **Patient ID** | **Pre-tx** | **Post-tx** | **Difference** | **Patient ID** | **Pre-tx** | **Post-tx** | **Difference** |
| Nonex01 | 1.438 | 1.490 | 0.052 | Ex01 | 1.464 | 1.502 | 0.038 |
| Nonex02 | 1.434 | 1.502 | 0.068 | Ex02 | 1.468 | 1.410 | -0.058 |
| Nonex03 | 1.492 | 1.501 | 0.009 | Ex03 | 1.456 | 1.487 | 0.031 |
| Nonex04 | 1.448 | 1.499 | 0.051 | Ex04 | 1.537 | 1.539 | 0.002 |
| Nonex05 | 1.592 | 1.569 | -0.023 | Ex05 | 1.519 | 1.454 | -0.065 |
| Nonex06 | 1.473 | 1.488 | 0.015 | Ex06 | 1.496 | 1.440 | -0.056 |
| Nonex07 | 1.495 | 1.533 | 0.038 | Ex07 | 1.484 | 1.521 | 0.037 |
| Nonex08 | 1.457 | 1.437 | -0.020 | Ex08 | 1.517 | 1.502 | -0.015 |
| Nonex09 | 1.462 | 1.408 | -0.054 | Ex09 | 1.518 | 1.510 | -0.008 |
| Nonex10 | 1.501 | 1.490 | -0.011 | Ex10 | 1.421 | 1.485 | 0.064 |
| Nonex11 | 1.462 | 1.473 | 0.011 | Ex11 | 1.495 | 1.484 | -0.011 |
| Nonex12 | 1.477 | 1.484 | 0.007 | Ex12 | 1.491 | 1.520 | 0.029 |
| Nonex13 | 1.498 | 1.480 | -0.018 | Ex13 | 1.530 | 1.557 | 0.027 |
| Nonex14 | 1.518 | 1.501 | -0.017 | Ex14 | 1.530 | 1.504 | -0.026 |
| Nonex15 | 1.515 | 1.489 | -0.026 | Ex15 | 1.532 | 1.544 | 0.012 |
| Nonex16 | 1.463 | 1.490 | 0.027 | Ex16 | 1.488 | 1.473 | -0.015 |
| Nonex17 | 1.456 | 1.444 | -0.012 | Ex17 | 1.560 | 1.548 | -0.012 |
| Nonex18 | 1.481 | 1.439 | -0.042 | Ex18 | 1.468 | 1.467 | -0.001 |
| Nonex19 | 1.512 | 1.515 | 0.003 | Ex19 | 1.517 | 1.550 | 0.033 |
| Nonex20 | 1.479 | 1.495 | 0.016 | Ex20 | 1.497 | 1.450 | -0.047 |
| Nonex21 | 1.485 | 1.487 | 0.002 | Ex21 | 1.484 | 1.436 | -0.048 |
| Nonex22 | 1.553 | 1.548 | -0.005 | Ex22 | 1.469 | 1.473 | 0.004 |
| Nonex23 | 1.454 | 1.417 | -0.037 | Ex23 | 1.499 | 1.543 | 0.044 |
| Nonex24 | 1.516 | 1.489 | -0.027 | Ex24 | 1.545 | 1.546 | 0.001 |
| Nonex25 | 1.443 | 1.442 | -0.001 | Ex25 | 1.472 | 1.490 | 0.018 |
| Nonex26 | 1.506 | 1.501 | -0.005 | Ex26 | 1.505 | 1.463 | -0.042 |
| Nonex27 | 1.509 | 1.553 | 0.044 | Ex27 | 1.485 | 1.529 | 0.044 |
| Nonex28 | 1.444 | 1.469 | 0.025 | Ex28 | 1.527 | 1.512 | -0.015 |
| Nonex29 | 1.540 | 1.543 | 0.003 |  | | | |
| Nonex30 | 1.454 | 1.489 | 0.035 |  |  |  |  |
| Nonex31 | 1.487 | 1.426 | -0.061 |  |  |  |  |
| Nonex32 | 1.535 | 1.509 | -0.026 |  |  |  |  |
| Nonex33 | 1.443 | 1.374 | -0.069 |  |  |  |  |
| **Median** | 1.481 | 1.489 | -0.001 | **Median** | 1.497 | 1.502 | 0.000 |
| **Q1** | 1.456 | 1.469 | -0.023 | **Q1** | 1.481 | 1.472 | -0.018 |
| **Q3** | 1.509 | 1.501 | 0.016 | **Q3** | 1.521 | 1.532 | 0.030 |

Pre-tx: Pre-treatment; Post-tx: Post-treatment; Q1: first quartile; Q3: third quartile.

**Supplementary Table 3.** Average distance (in mm) between corresponding points of the outlines of pre- and post-treatment palatal rugae.

| **Patient ID** | **Distance** | **Patient ID** | **Distance** |
| --- | --- | --- | --- |
| Nonex01 | 0.35 | Ex01 | 0.34 |
| Nonex02 | 0.18 | Ex02 | 0.46 |
| Nonex03 | 0.16 | Ex03 | 0.34 |
| Nonex04 | 0.26 | Ex04 | 0.32 |
| Nonex05 | 0.25 | Ex05 | 0.43 |
| Nonex06 | 0.29 | Ex06 | 0.37 |
| Nonex07 | 0.32 | Ex07 | 0.32 |
| Nonex08 | 0.23 | Ex08 | 0.23 |
| Nonex09 | 0.58 | Ex09 | 0.42 |
| Nonex10 | 0.30 | Ex10 | 0.60 |
| Nonex11 | 0.31 | Ex11 | 0.41 |
| Nonex12 | 0.24 | Ex12 | 0.52 |
| Nonex13 | 0.19 | Ex13 | 0.37 |
| Nonex14 | 0.34 | Ex14 | 0.24 |
| Nonex15 | 0.43 | Ex15 | 0.36 |
| Nonex16 | 0.22 | Ex16 | 0.57 |
| Nonex17 | 0.15 | Ex17 | 0.37 |
| Nonex18 | 0.44 | Ex18 | 0.59 |
| Nonex19 | 0.22 | Ex19 | 0.31 |
| Nonex20 | 0.17 | Ex20 | 0.40 |
| Nonex21 | 0.34 | Ex21 | 0.63 |
| Nonex22 | 0.18 | Ex22 | 0.58 |
| Nonex23 | 0.52 | Ex23 | 0.51 |
| Nonex24 | 0.40 | Ex24 | 0.20 |
| Nonex25 | 0.15 | Ex25 | 0.41 |
| Nonex26 | 0.30 | Ex26 | 0.38 |
| Nonex27 | 0.66 | Ex27 | 0.56 |
| Nonex28 | 0.34 | Ex28 | 0.37 |
| Nonex29 | 0.25 |  | |
| Nonex30 | 0.24 |  |  |
| Nonex31 | 0.54 |  |  |
| Nonex32 | 0.27 |  |  |
| Nonex33 | 0.17 |  |  |
| **Median** | 0.27 | **Median** | 0.39 |
| **Q1** | 0.22 | **Q1** | 0.34 |
| **Q3** | 0.34 | **Q3** | 0.51 |

Pre-tx: Pre-treatment; Post-tx: Post-treatment; Q1: first quartile; Q3: third quartile.

**Supplementary Table 4.** Arch depth (in mm) defined as the shortest distance from central incisors’ contact point to the line connecting the palatal grooves of the first molars.

| **Non-extraction group** | | | | **Extraction group** | | | |
| --- | --- | --- | --- | --- | --- | --- | --- |
| **Patient ID** | **Pre-tx** | **Post-tx** | **Difference** | **Patient ID** | **Pre-tx** | **Post-tx** | **Difference** |
| Nonex01 | 32.4 | 32.7 | 0.3 | Ex01 | 28.8 | 21.8 | -7.0 |
| Nonex02 | 29.3 | 29.7 | 0.4 | Ex02 | 30.3 | 27.0 | -3.3 |
| Nonex03 | 29.7 | 30.0 | 0.3 | Ex03 | 29.8 | 22.9 | -6.9 |
| Nonex04 | 30.3 | 28.5 | -1.8 | Ex04 | 32.9 | 25.4 | -7.5 |
| Nonex05 | 31.9 | 28.2 | -3.7 | Ex05 | 34.9 | 27.0 | -7.9 |
| Nonex06 | 31.1 | 30.5 | -0.6 | Ex06 | 27.6 | 22.8 | -4.8 |
| Nonex07 | 33.2 | 32.9 | -0.3 | Ex07 | 32.5 | 26.1 | -6.4 |
| Nonex08 | 28.5 | 30.6 | 2.1 | Ex08 | 29.5 | 24.3 | -5.2 |
| Nonex09 | 33.9 | 29.1 | -4.8 | Ex09 | 33.8 | 22.3 | -11.5 |
| Nonex10 | 34.7 | 33.3 | -1.4 | Ex10 | 37.3 | 28.1 | -9.2 |
| Nonex11 | 33.3 | 31.3 | -2.0 | Ex11 | 29.0 | 23.7 | -5.3 |
| Nonex12 | 30.3 | 31.5 | 1.2 | Ex12 | 35.8 | 28.1 | -7.7 |
| Nonex13 | 30.3 | 30.8 | 0.5 | Ex13 | 27.9 | 27.3 | -0.6 |
| Nonex14 | 33.2 | 31.9 | -1.3 | Ex14 | 30.8 | 24.0 | -6.8 |
| Nonex15 | 32.1 | 31.0 | -1.1 | Ex15 | 29.7 | 25.9 | -3.8 |
| Nonex16 | 32.9 | 32.0 | -0.9 | Ex16 | 27.8 | 25.2 | -2.6 |
| Nonex17 | 30.1 | 29.9 | -0.2 | Ex17 | 32.2 | 25.3 | -6.9 |
| Nonex18 | 35.0 | 31.5 | -3.5 | Ex18 | 32.3 | 25.7 | -6.6 |
| Nonex19 | 31.2 | 31.4 | 0.2 | Ex19 | 36.0 | 27.4 | -8.6 |
| Nonex20 | 31.0 | 33.1 | 2.1 | Ex20 | 29.4 | 23.5 | -5.9 |
| Nonex21 | 35.4 | 32.8 | -2.6 | Ex21 | 31.1 | 24.5 | -6.6 |
| Nonex22 | 31.1 | 28.5 | -2.6 | Ex22 | 32.8 | 25.6 | -7.2 |
| Nonex23 | 36.0 | 31.4 | -4.6 | Ex23 | 34.2 | 28.5 | -5.7 |
| Nonex24 | 30.8 | 30.9 | 0.1 | Ex24 | 32.1 | 27.9 | -4.2 |
| Nonex25 | 32.7 | 32.8 | 0.1 | Ex25 | 33.7 | 23.2 | -10.5 |
| Nonex26 | 33.5 | 29.1 | -4.4 | Ex26 | 36.1 | 25.8 | -10.3 |
| Nonex27 | 36.7 | 33.7 | -3.0 | Ex27 | 33.0 | 25.3 | -7.7 |
| Nonex28 | 38.4 | 34.2 | -4.2 | Ex28 | 27.6 | 24.5 | -3.1 |
| Nonex29 | 30.8 | 32.3 | 1.5 |  | | | |
| Nonex30 | 30.3 | 31.3 | 1.0 |  |  |  |  |
| Nonex31 | 32.5 | 33.0 | 0.5 |  |  |  |  |
| Nonex32 | 31.4 | 30.4 | -1.0 |  |  |  |  |
| Nonex33 | 27.9 | 30.4 | 2.5 |  |  |  |  |
| **Median** | 31.9 | 31.3 | -0.6 | **Median** | 32.2 | 25.4 | -6.7 |
| **Q1** | 30.3 | 30.4 | -2.6 | **Q1** | 29.5 | 23.9 | -7.7 |
| **Q3** | 33.3 | 32.7 | 0.4 | **Q3** | 33.7 | 27.0 | -5.1 |

Pre-tx: Pre-treatment; Post-tx: Post-treatment; Q1: first quartile; Q3: third quartile.

**Supplementary Table 5.** Palatal surface (in mm^2^), as established in Step 2 of the methodology.

| **Non-extraction group** | | | | **Extraction group** | | | |
| --- | --- | --- | --- | --- | --- | --- | --- |
| **Patient ID** | **Pre-tx** | **Post-tx** | **Difference** | **Patient ID** | **Pre-tx** | **Post-tx** | **Difference** |
| Nonex01 | 1126.5 | 1316.9 | 190.4 | Ex01 | 988.5 | 715.4 | -273.1 |
| Nonex02 | 1027.0 | 1080.8 | 53.8 | Ex02 | 1102.5 | 1004.0 | -98.5 |
| Nonex03 | 992.6 | 1185.8 | 193.2 | Ex03 | 1232.2 | 1034.6 | -197.6 |
| Nonex04 | 972.3 | 1066.6 | 94.3 | Ex04 | 1146.8 | 1055.2 | -91.6 |
| Nonex05 | 1091.4 | 920.1 | -171.3 | Ex05 | 1007.7 | 921.5 | -86.2 |
| Nonex06 | 903.0 | 1047.6 | 144.6 | Ex06 | 1235.7 | 966.7 | -269.0 |
| Nonex07 | 1214.7 | 1310.8 | 96.1 | Ex07 | 1084.2 | 943.6 | -140.6 |
| Nonex08 | 918.2 | 948.4 | 30.2 | Ex08 | 1097.2 | 816.0 | -281.2 |
| Nonex09 | 1142.1 | 1115.4 | -26.7 | Ex09 | 1419.7 | 1018.4 | -401.3 |
| Nonex10 | 1305.4 | 1252.8 | -52.6 | Ex10 | 1343.3 | 1184.4 | -158.9 |
| Nonex11 | 1316.4 | 1382.2 | 65.8 | Ex11 | 1020.4 | 1014.5 | -5.9 |
| Nonex12 | 906.0 | 1004.1 | 98.1 | Ex12 | 1288.3 | 1036.9 | -251.4 |
| Nonex13 | 1070.2 | 1131.9 | 61.7 | Ex13 | 898.2 | 869.9 | -28.3 |
| Nonex14 | 1397.1 | 1354.9 | -42.2 | Ex14 | 1196.0 | 980.0 | -216.0 |
| Nonex15 | 1016.4 | 1038.2 | 21.8 | Ex15 | 1269.4 | 1046.9 | -222.5 |
| Nonex16 | 992.7 | 1057.0 | 64.3 | Ex16 | 957.9 | 985.3 | 27.4 |
| Nonex17 | 913.2 | 893.2 | -20.0 | Ex17 | 993.2 | 949.3 | -43.9 |
| Nonex18 | 1116.9 | 1058.3 | -58.6 | Ex18 | 1021.3 | 879.5 | -141.8 |
| Nonex19 | 1161.0 | 1223.0 | 62.0 | Ex19 | 1479.4 | 1294.7 | -184.7 |
| Nonex20 | 1113.4 | 1273.7 | 160.3 | Ex20 | 888.6 | 809.7 | -78.9 |
| Nonex21 | 1218.4 | 1289.5 | 71.1 | Ex21 | 1040.1 | 932.4 | -107.7 |
| Nonex22 | 963.7 | 992.2 | 28.5 | Ex22 | 1260.6 | 1102.9 | -157.7 |
| Nonex23 | 1028.9 | 1161.8 | 132.9 | Ex23 | 1568.3 | 1341.7 | -226.6 |
| Nonex24 | 1152.6 | 1244.1 | 91.5 | Ex24 | 1134.5 | 994.4 | -140.1 |
| Nonex25 | 1226.2 | 1292.8 | 66.6 | Ex25 | 1091.7 | 1001.7 | -90.0 |
| Nonex26 | 1143.1 | 1048.7 | -94.4 | Ex26 | 1152.4 | 1070.1 | -82.3 |
| Nonex27 | 1101.2 | 1180.0 | 78.8 | Ex27 | 1004.4 | 920.3 | -84.1 |
| Nonex28 | 1266.3 | 1268.6 | 2.3 | Ex28 | 816.1 | 854.4 | 38.3 |
| Nonex29 | 1241.4 | 1248.1 | 6.7 |  | | | |
| Nonex30 | 1105.1 | 1138.1 | 33.0 |  |  |  |  |
| Nonex31 | 1058.5 | 1214.3 | 155.8 |  |  |  |  |
| Nonex32 | 1034.0 | 957.5 | -76.5 |  |  |  |  |
| Nonex33 | 1014.7 | 1161.4 | 146.7 |  |  |  |  |
| **Median** | 1101.2 | 1161.4 | 62.0 | **Median** | 1099.9 | 989.9 | -140.4 |
| **Q1** | 1014.7 | 1048.7 | 2.3 | **Q1** | 1006.9 | 921.2 | -217.6 |
| **Q3** | 1161.0 | 1252.8 | 96.1 | **Q3** | 1241.9 | 1039.4 | -83.7 |

Pre-tx: Pre-treatment; Post-tx: Post-treatment; Q1: first quartile; Q3: third quartile.

**Supplementary Table 6.** Rugae’s convex hull area on flattened surfaces (in mm^2^)

| **Non-extraction group** | | | | **Extraction group** | | | |
| --- | --- | --- | --- | --- | --- | --- | --- |
| **Patient ID** | **Pre-tx** | **Post-tx** | **Difference** | **Patient ID** | **Pre-tx** | **Post-tx** | **Difference** |
| Nonex01 | 458.0 | 472.7 | 14.7 | Ex01 | 302.8 | 255.6 | -47.2 |
| Nonex02 | 280.9 | 312.7 | 31.8 | Ex02 | 241.7 | 249.9 | 8.2 |
| Nonex03 | 414.5 | 417.3 | 2.8 | Ex03 | 391.8 | 475.4 | 83.6 |
| Nonex04 | 456.7 | 454.3 | -2.4 | Ex04 | 536.1 | 508.6 | -27.5 |
| Nonex05 | 336.4 | 507.8 | 171.4 | Ex05 | 312.7 | 325.8 | 13.1 |
| Nonex06 | 341.8 | 431.3 | 89.5 | Ex06 | 385.2 | 374.8 | -10.4 |
| Nonex07 | 479.2 | 479.2 | 0.0 | Ex07 | 382.6 | 399.9 | 17.3 |
| Nonex08 | 587.8 | 567.9 | -19.9 | Ex08 | 367.9 | 351.7 | -16.2 |
| Nonex09 | 325.0 | 330.2 | 5.2 | Ex09 | 638.8 | 607.7 | -31.1 |
| Nonex10 | 464.8 | 447.9 | -16.9 | Ex10 | 500.5 | 463.9 | -36.6 |
| Nonex11 | 420.9 | 435.6 | 14.7 | Ex11 | 501.3 | 522.7 | 21.4 |
| Nonex12 | 380.7 | 427.2 | 46.5 | Ex12 | 591.2 | 556.9 | -34.3 |
| Nonex13 | 446.5 | 451.6 | 5.1 | Ex13 | 352.9 | 453.4 | 100.5 |
| Nonex14 | 457.5 | 513.6 | 56.1 | Ex14 | 472.4 | 438.0 | -34.4 |
| Nonex15 | 364.8 | 439.9 | 75.1 | Ex15 | 567.0 | 503.9 | -63.1 |
| Nonex16 | 358.3 | 375.0 | 16.7 | Ex16 | 331.7 | 387.9 | 56.2 |
| Nonex17 | 356.8 | 351.1 | -5.7 | Ex17 | 242.9 | 240.5 | -2.4 |
| Nonex18 | 336.3 | 385.8 | 49.5 | Ex18 | 385.9 | 388.9 | 3.0 |
| Nonex19 | 630.3 | 724.2 | 93.9 | Ex19 | 629.4 | 606.6 | -22.8 |
| Nonex20 | 472.7 | 452.9 | -19.8 | Ex20 | 324.3 | 344.8 | 20.5 |
| Nonex21 | 572.6 | 592.7 | 20.1 | Ex21 | 311.8 | 306.2 | -5.6 |
| Nonex22 | 180.2 | 194.3 | 14.1 | Ex22 | 373.5 | 348.9 | -24.6 |
| Nonex23 | 394.1 | 444.8 | 50.7 | Ex23 | 289.4 | 302.4 | 13.0 |
| Nonex24 | 301.8 | 326.1 | 24.3 | Ex24 | 493.7 | 513.6 | 19.9 |
| Nonex25 | 550.1 | 573.0 | 22.9 | Ex25 | 353.2 | 327.1 | -26.1 |
| Nonex26 | 448.0 | 378.9 | -69.1 | Ex26 | 211.8 | 231.6 | 19.8 |
| Nonex27 | 267.3 | 336.4 | 69.1 | Ex27 | 411.7 | 381.1 | -30.6 |
| Nonex28 | 488.9 | 480.9 | -8.0 | Ex28 | 235.1 | 257.6 | 22.5 |
| Nonex29 | 381.9 | 406.3 | 24.4 |  | | | |
| Nonex30 | 518.7 | 533.0 | 14.3 |  |  |  |  |
| Nonex31 | 393.0 | 344.3 | -48.7 |  |  |  |  |
| Nonex32 | 447.9 | 458.8 | 10.9 |  |  |  |  |
| Nonex33 | 315.7 | 333.5 | 17.8 |  |  |  |  |
| **Median** | 414.5 | 439.9 | 14.7 | **Median** | 378.1 | 384.5 | -4.0 |
| **Q1** | 341.8 | 375.0 | 0.0 | **Q1** | 312.5 | 320.9 | -28.3 |
| **Q3** | 464.8 | 479.2 | 46.5 | **Q3** | 495.4 | 482.5 | 19.8 |

Pre-tx: Pre-treatment; Post-tx: Post-treatment; Q1: first quartile; Q3: third quartile.
