# Supplementary material for: Palatal rugae change shape following orthodontic treatment: a comparison between extraction and non-extraction borderline cases using fractal analysis and 3D superimposition
Source: Eur J Orthod. 2024 Dec 7;47(1):cjae070. doi: 10.1093/ejo/cjae070 (PMC11631060; doi:10.1093/ejo/cjae070)
Supplement: cjae070_suppl_Supplementary_Figures_1-2 [file cjae070_suppl_supplementary_figures_1-2.docx]

**Supplementary figure 1:** Screenshots of 3D best-fit superimposition of pre- (blue) and post-treatment (red) palatal rugae for the extraction group with age at the beginning of treatment in parenthesis.

| **Ex01 (18 years)** | **Ex02 (15 years)** |
| --- | --- |
| 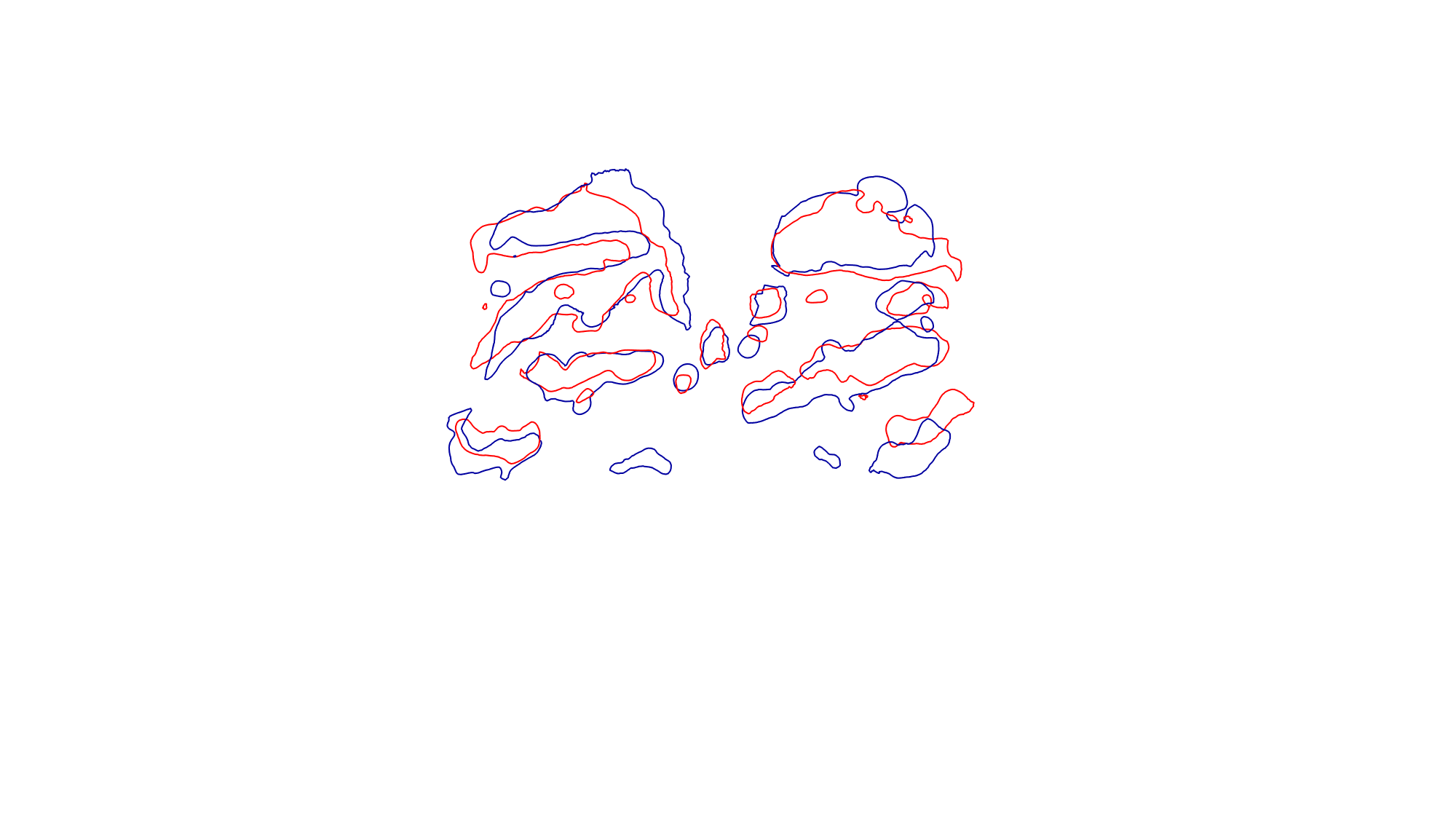 | 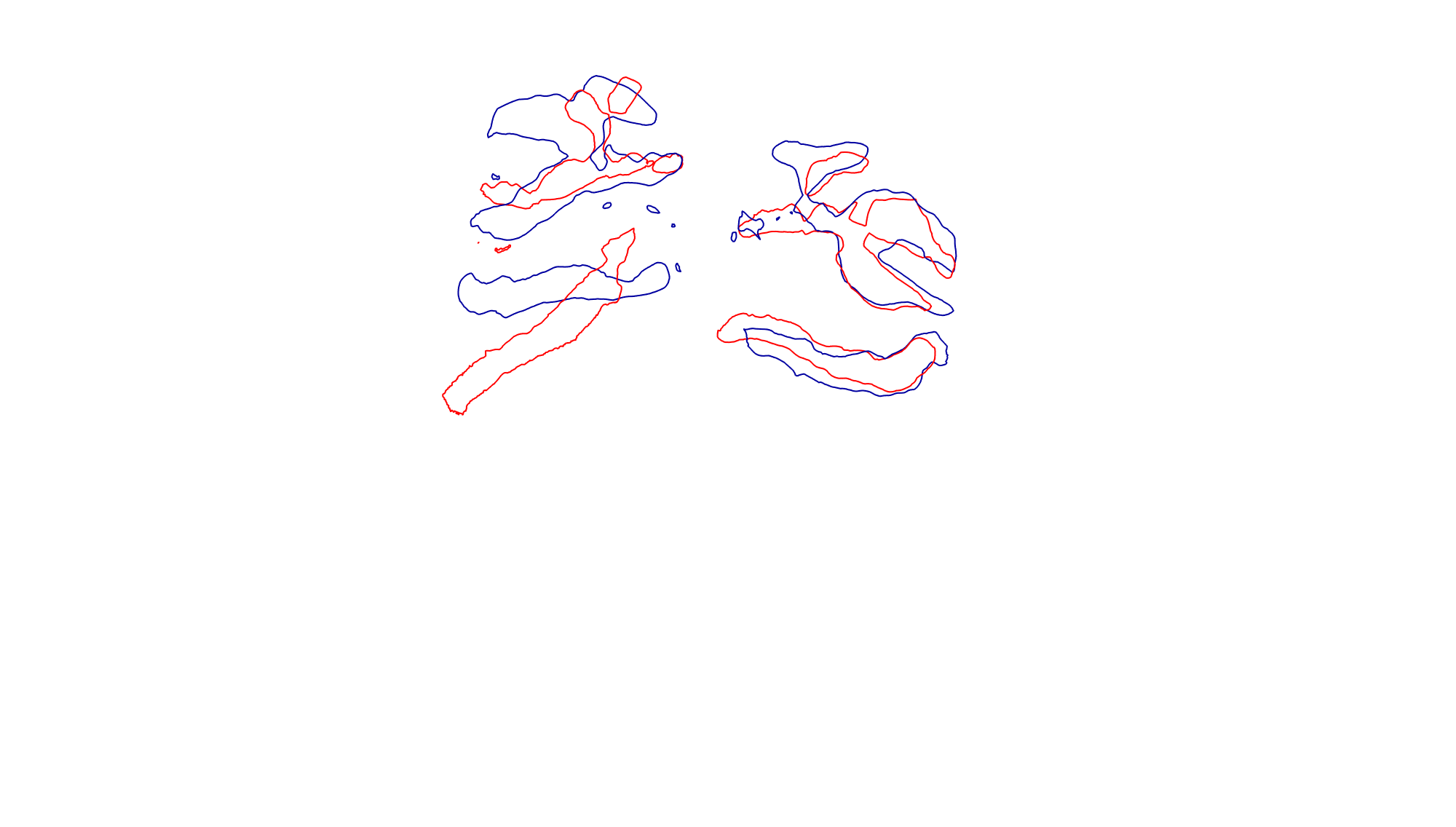 |
| **Ex03 (14 years)** | **Ex04 (13 years)** |
| 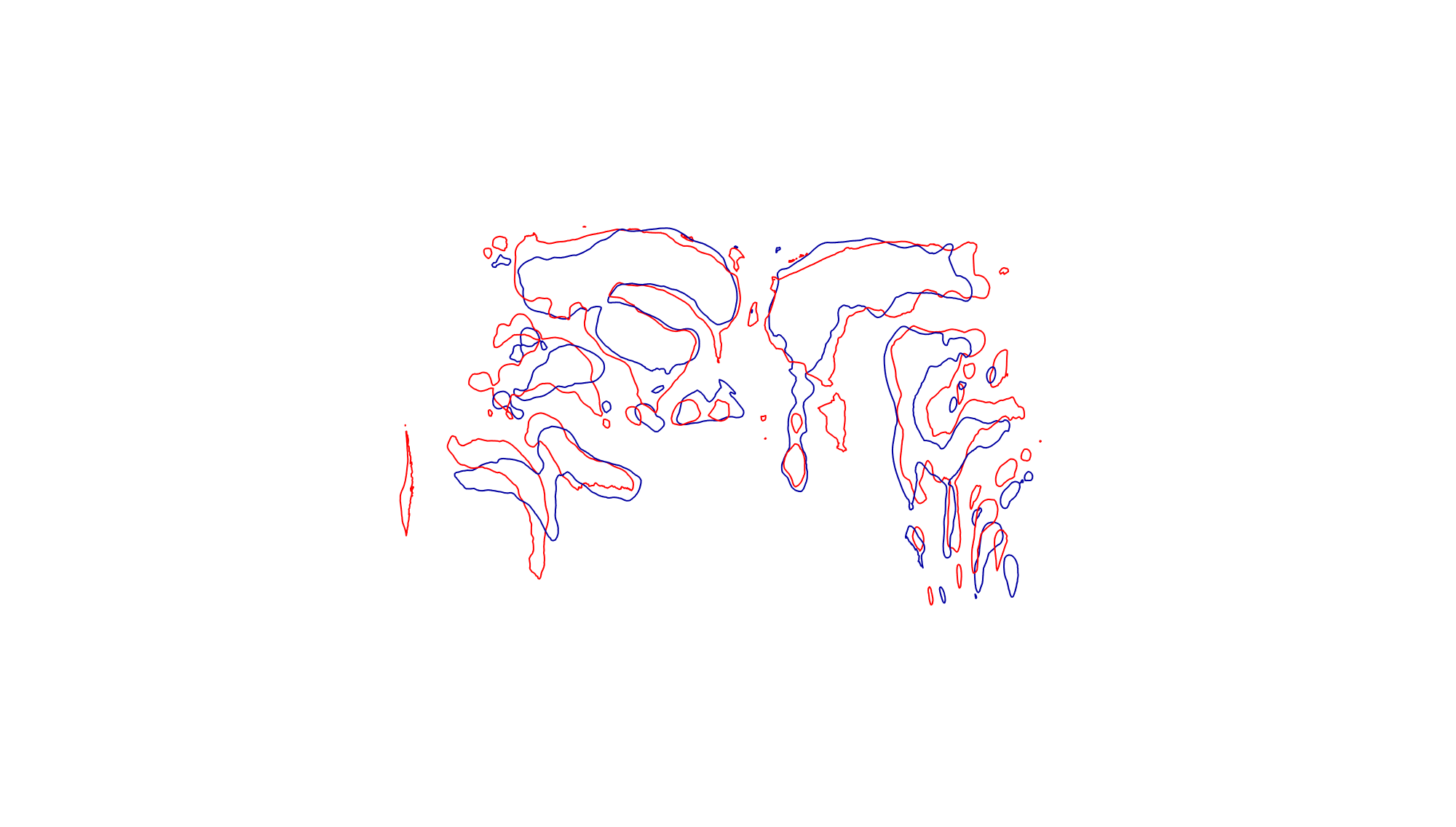 | 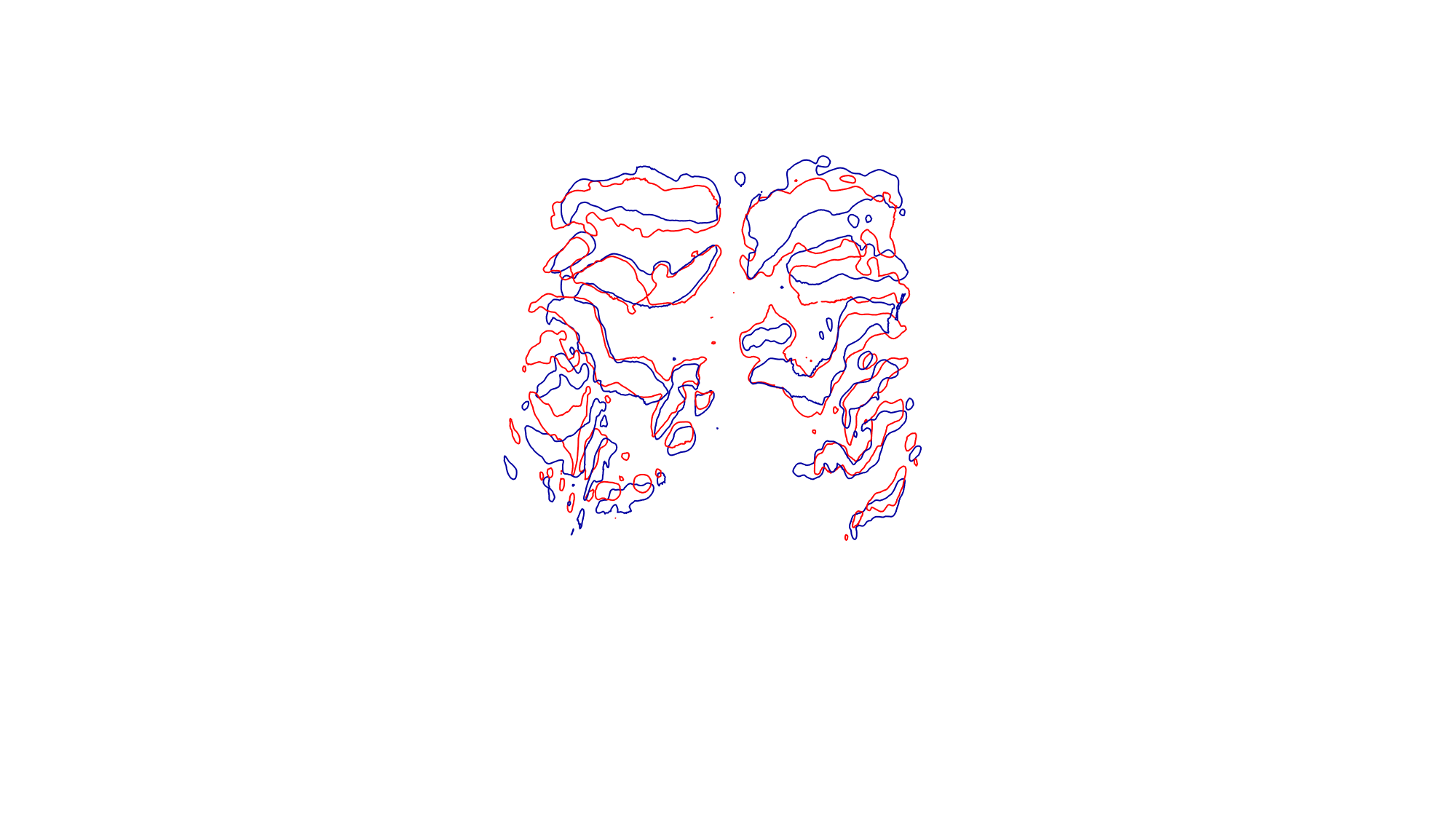 |
| **Ex05 (12 years)** | **Ex06 (18 years)** |
| 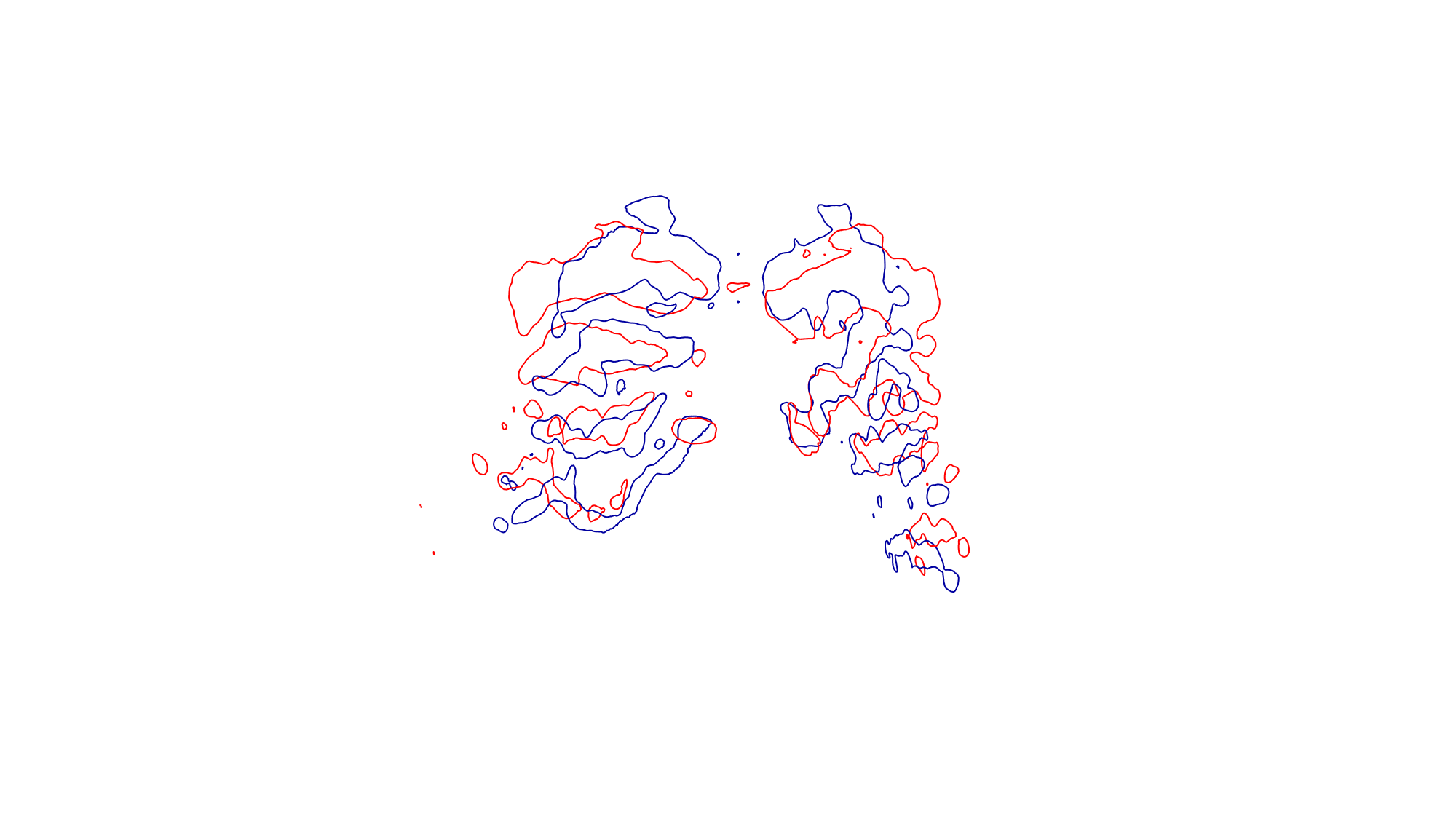 | 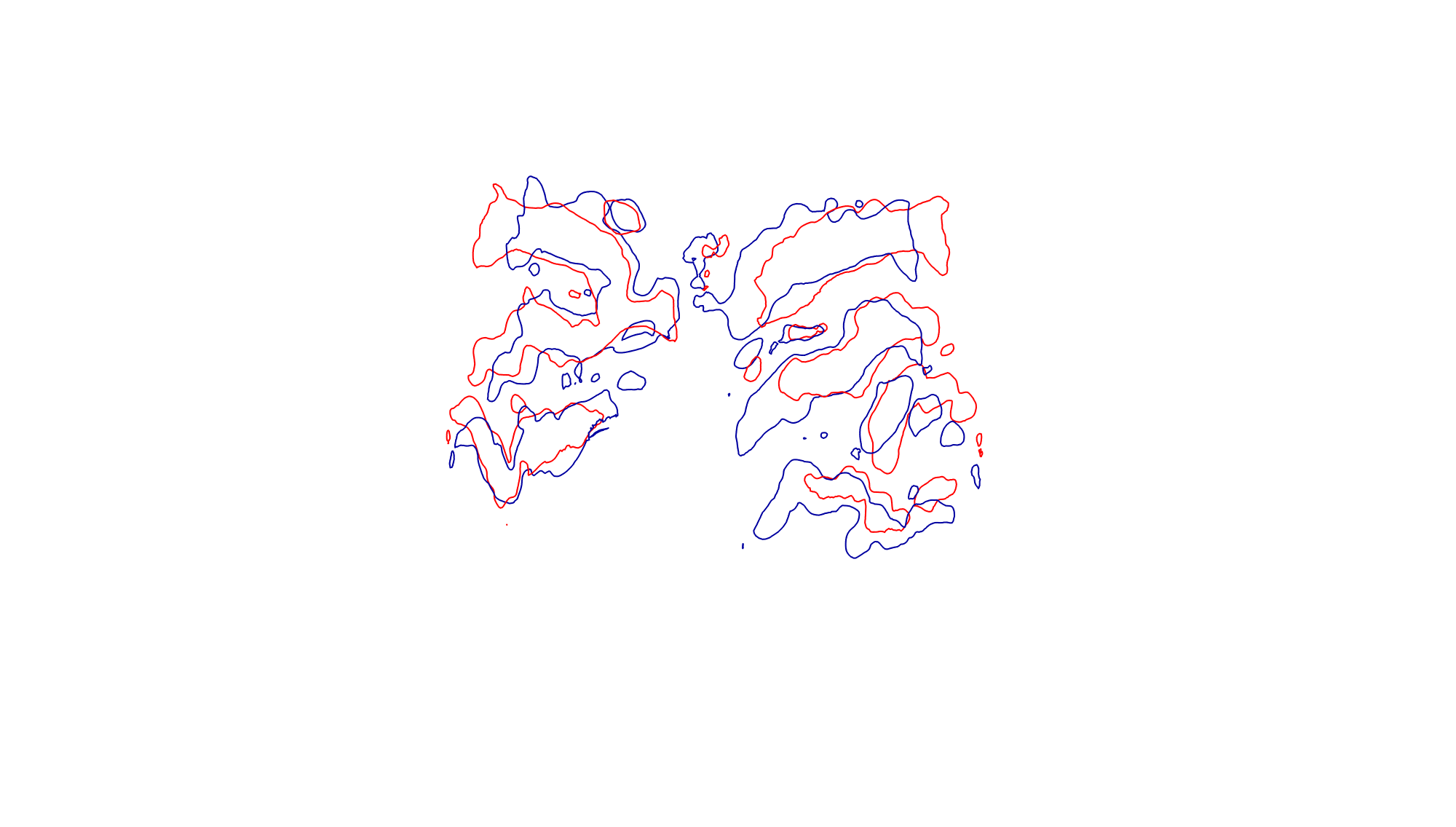 |
| **Ex07 (14 years)** | **Ex08 (13 years)** |
| 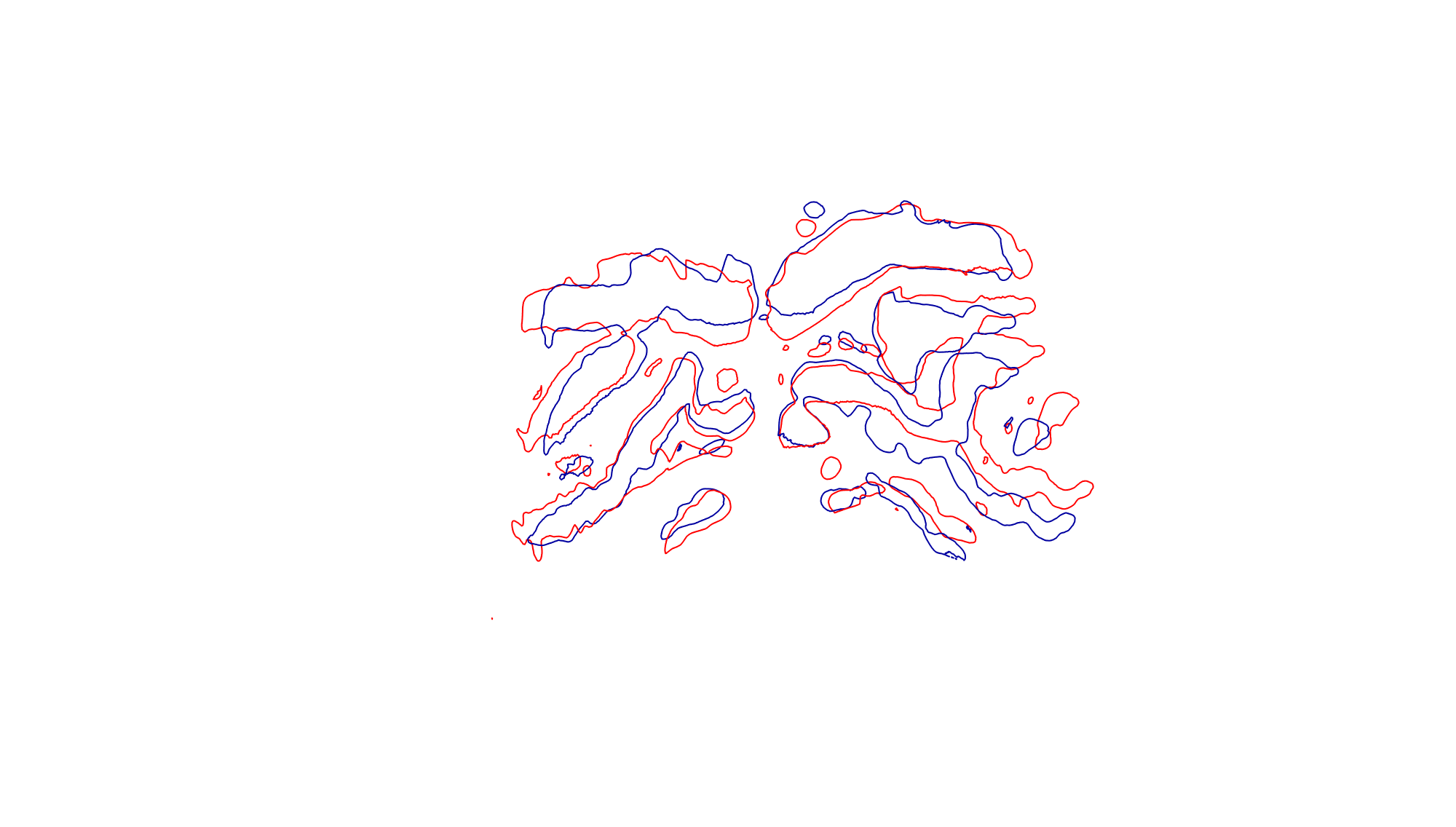 | 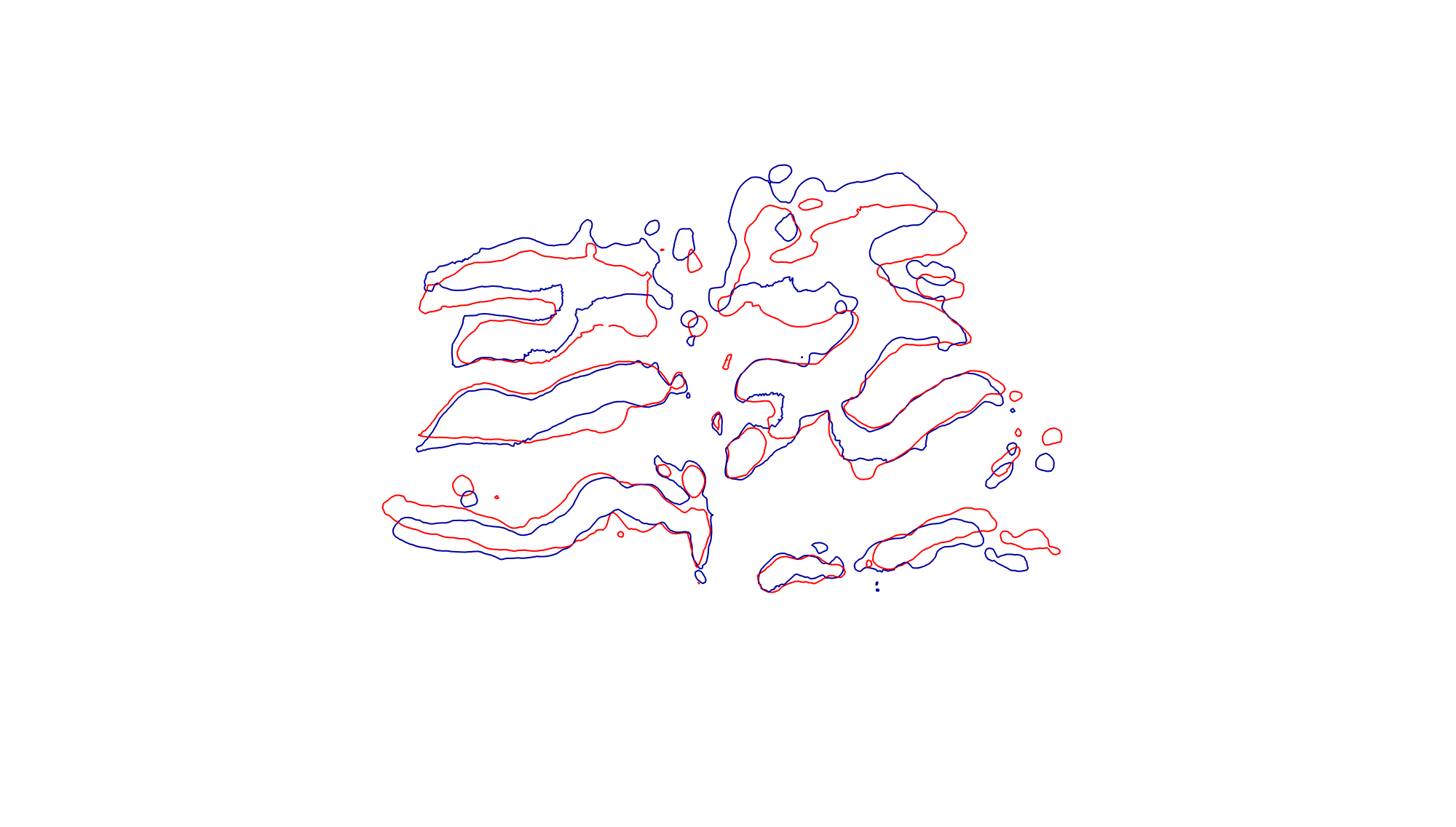 |
| **Ex09 (15 years)** | **Ex10 (11 years)** |
| 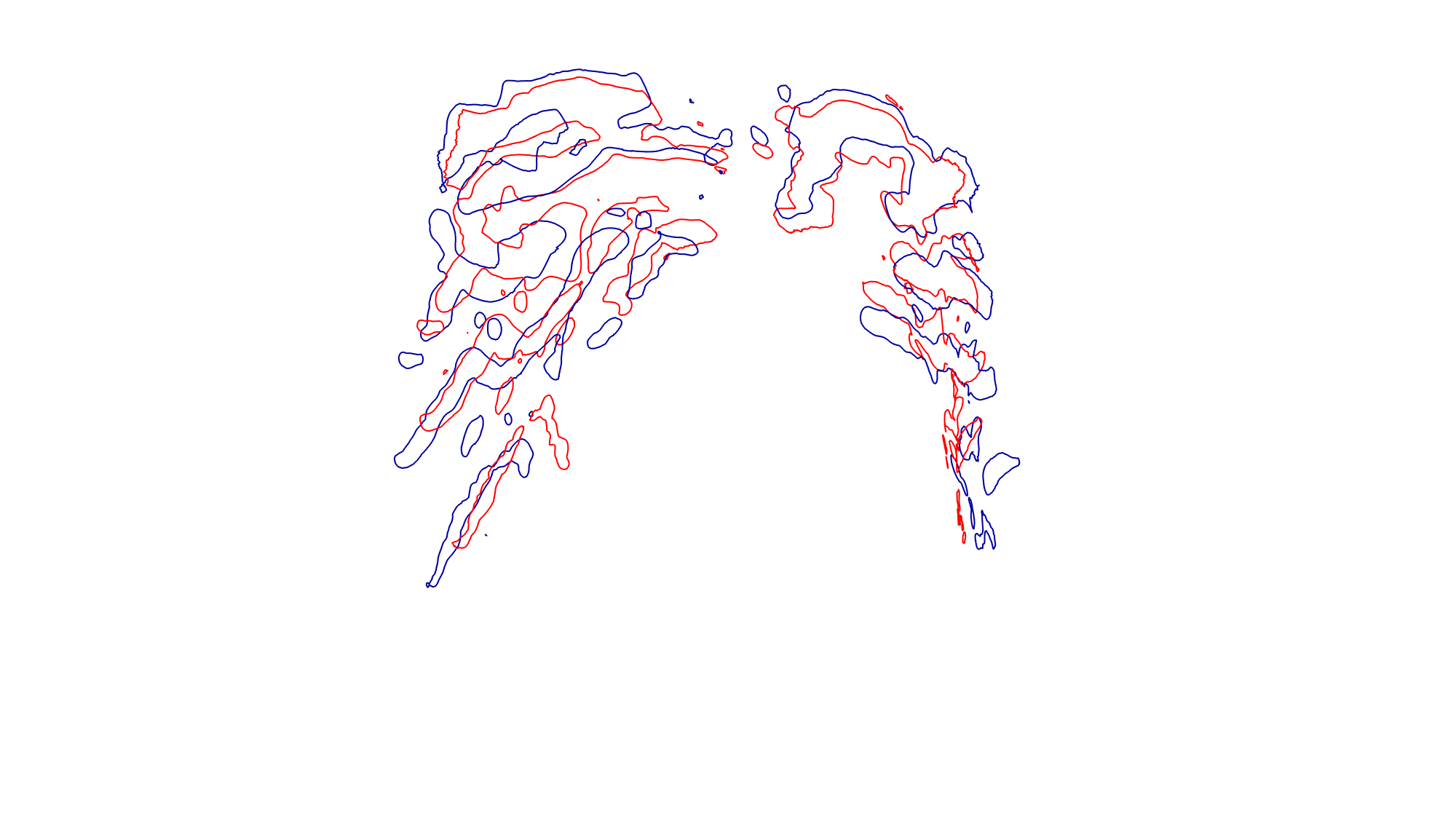 | 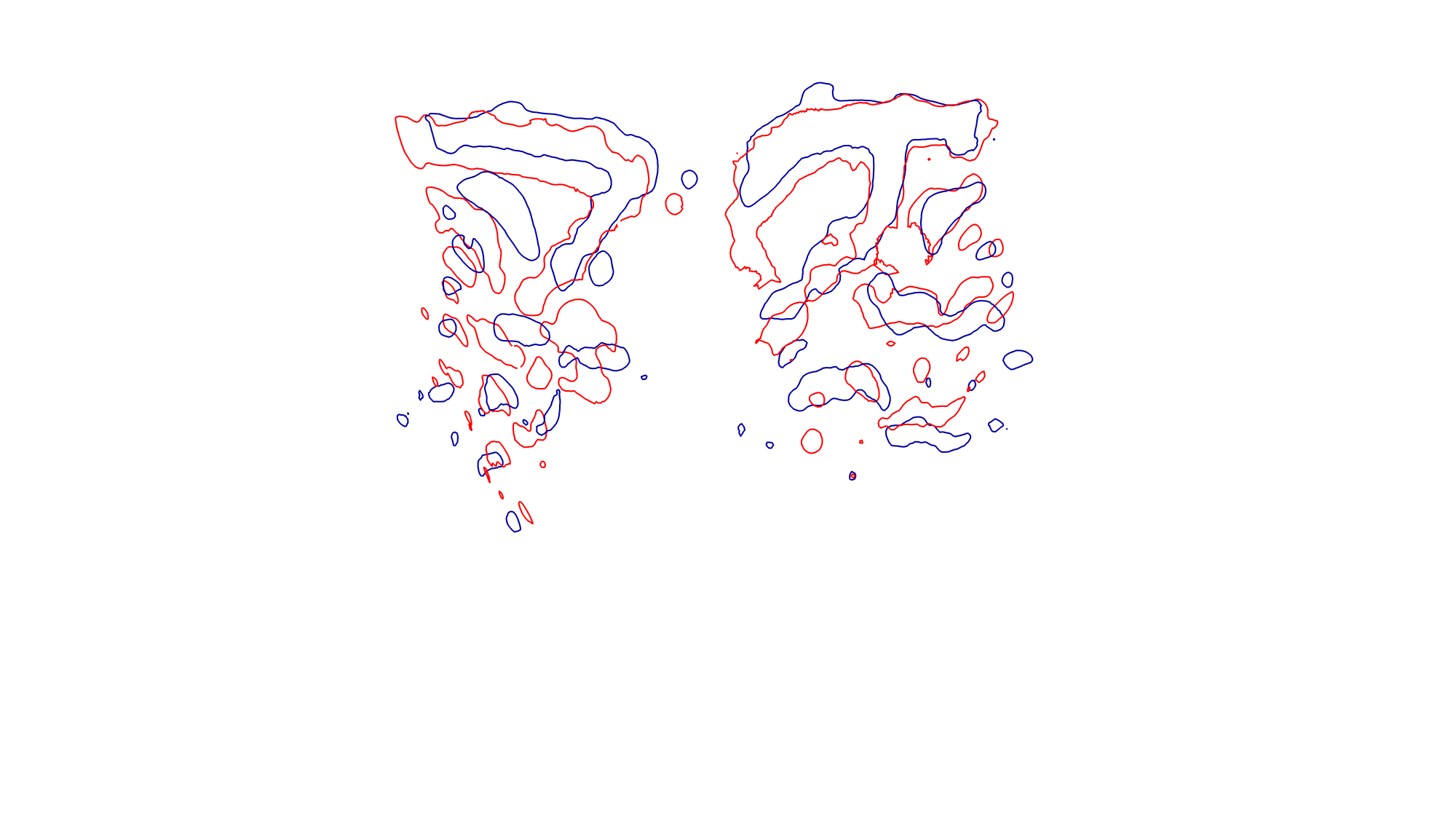 |
| **Ex11 (11 years)** | **Ex12 (14 years)** |
| 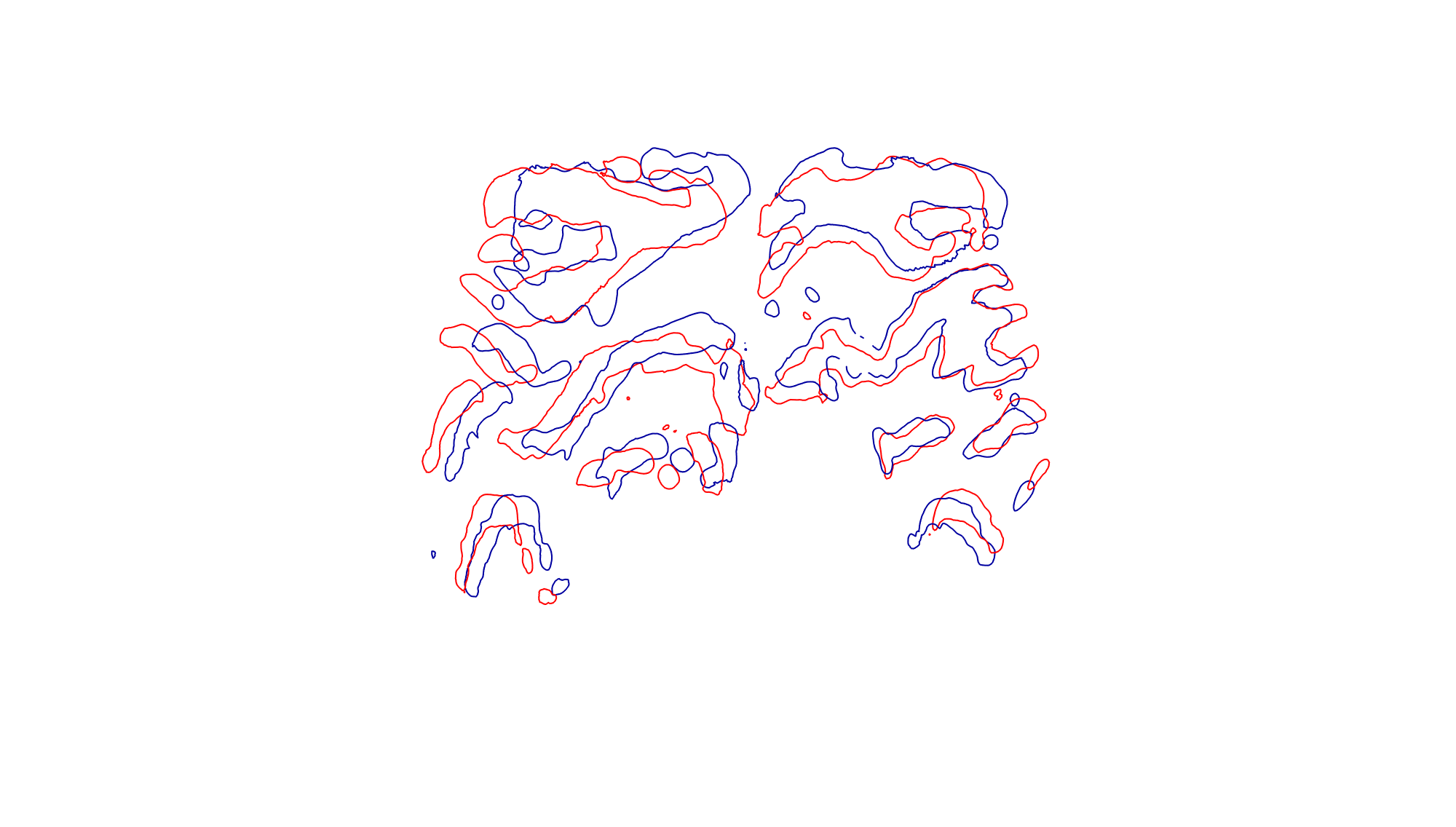 | 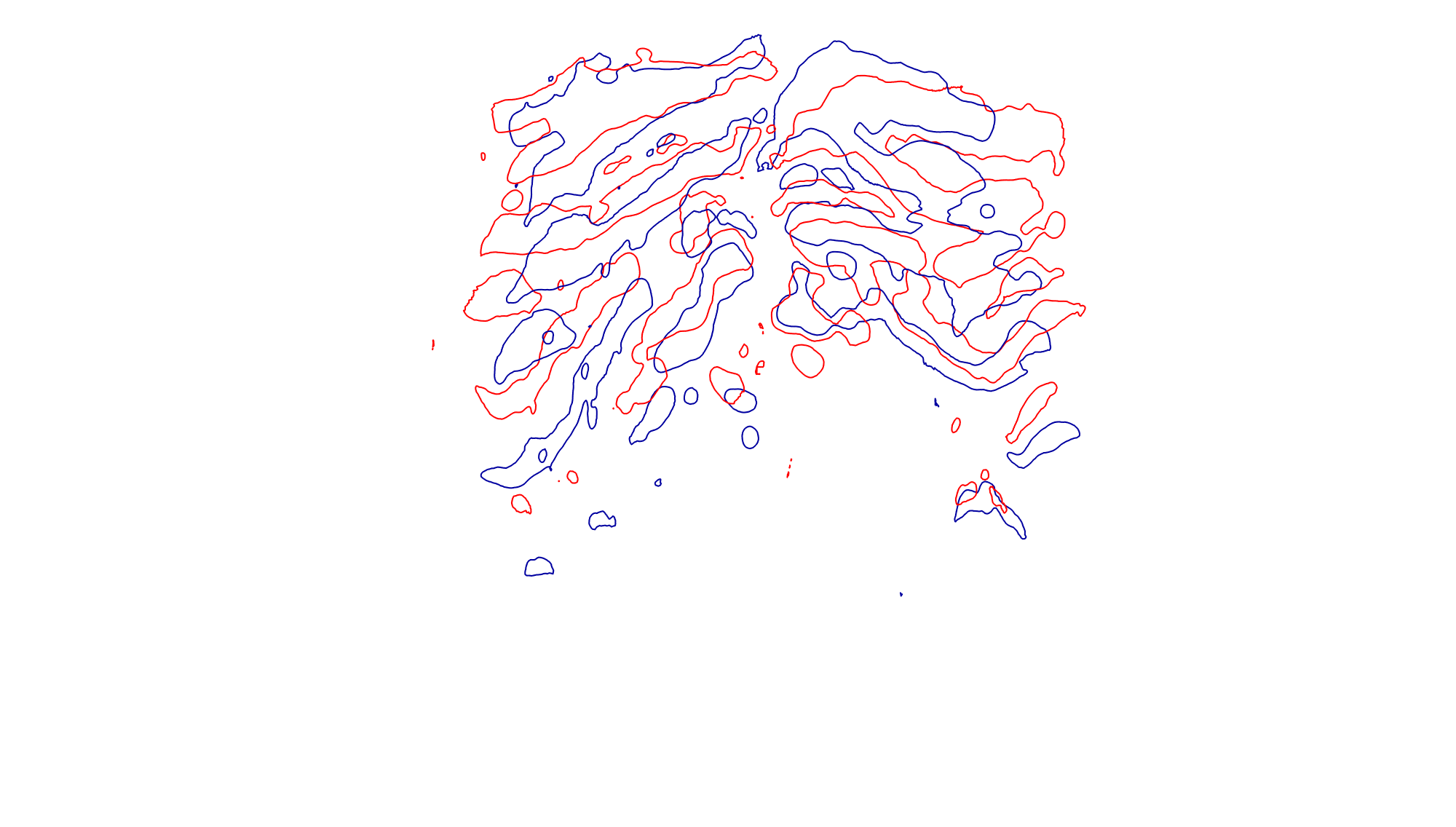 |
| **Ex13 (17 years)** | **Ex14 (13 years)** |
| 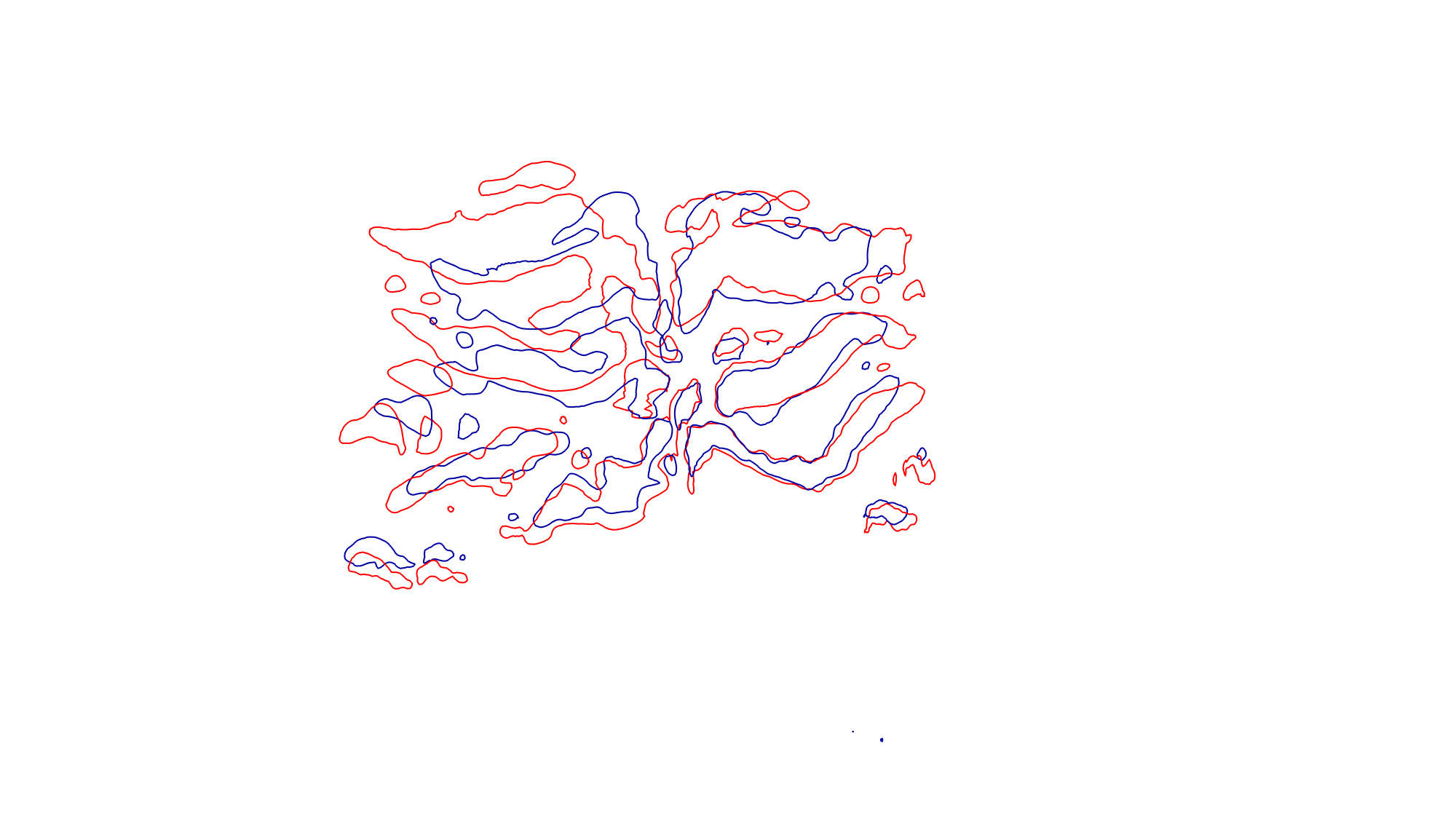 | 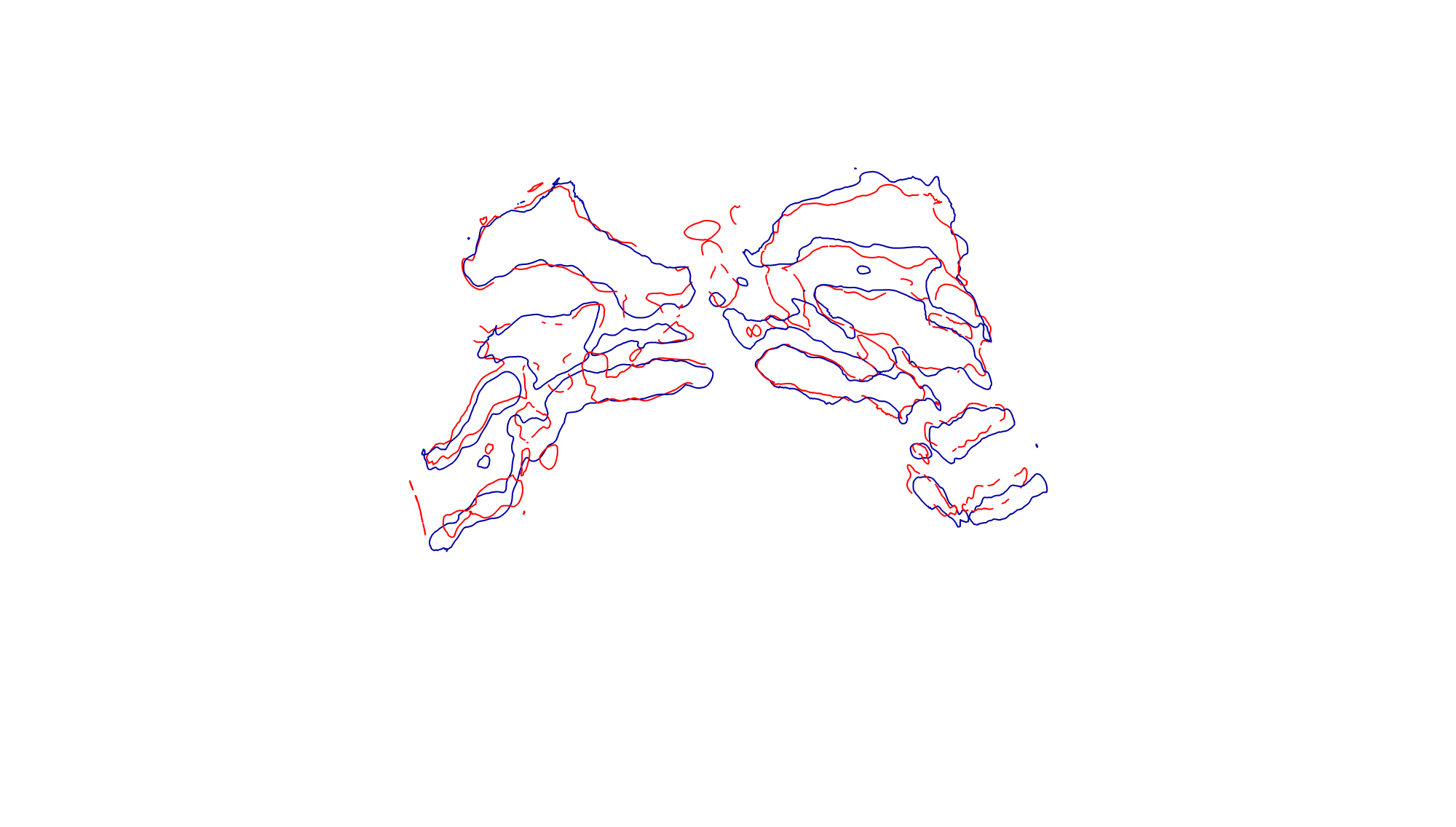 |
| **Ex15 (23 years)** | **Ex16 (13 years)** |
| 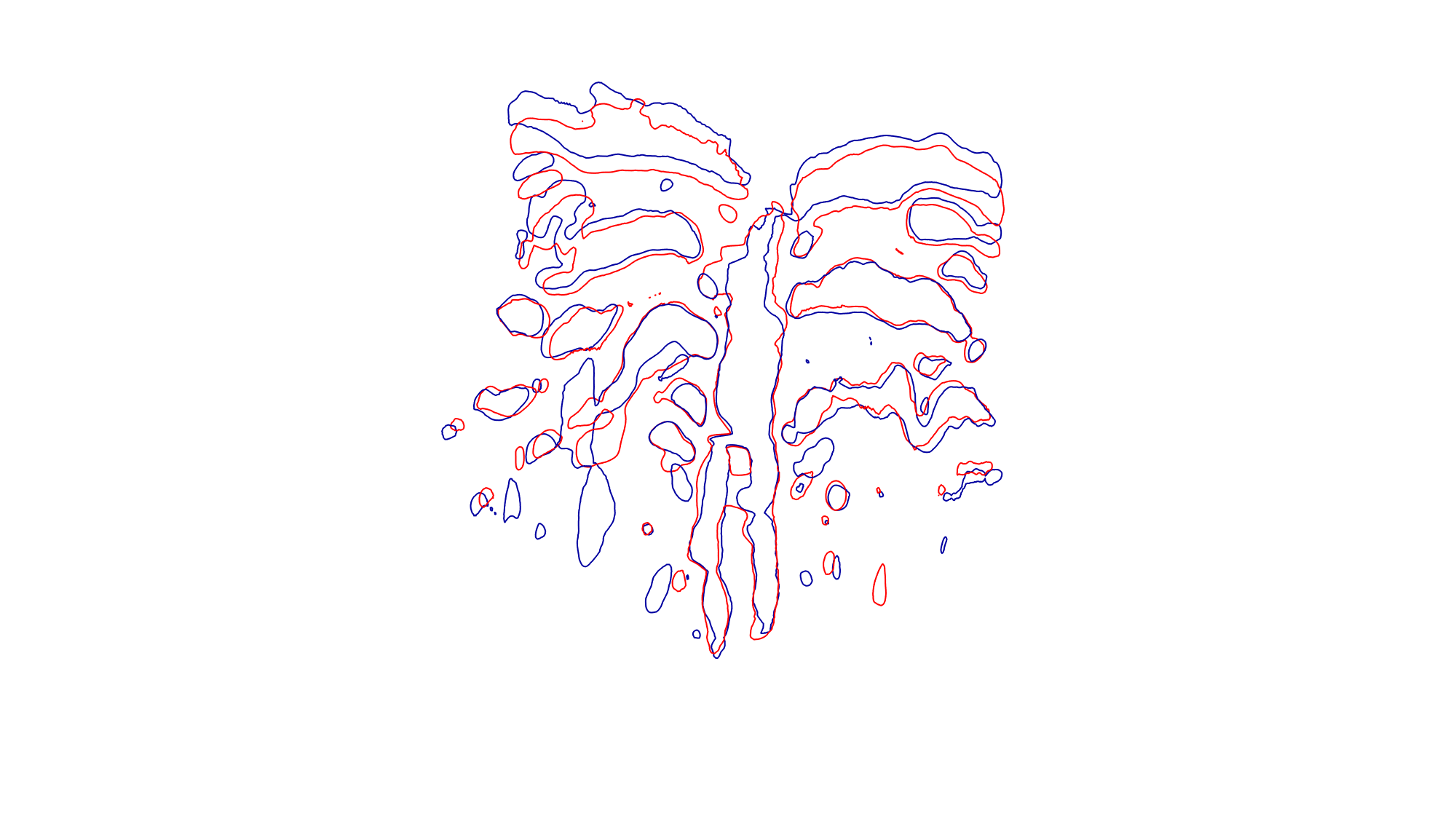 | 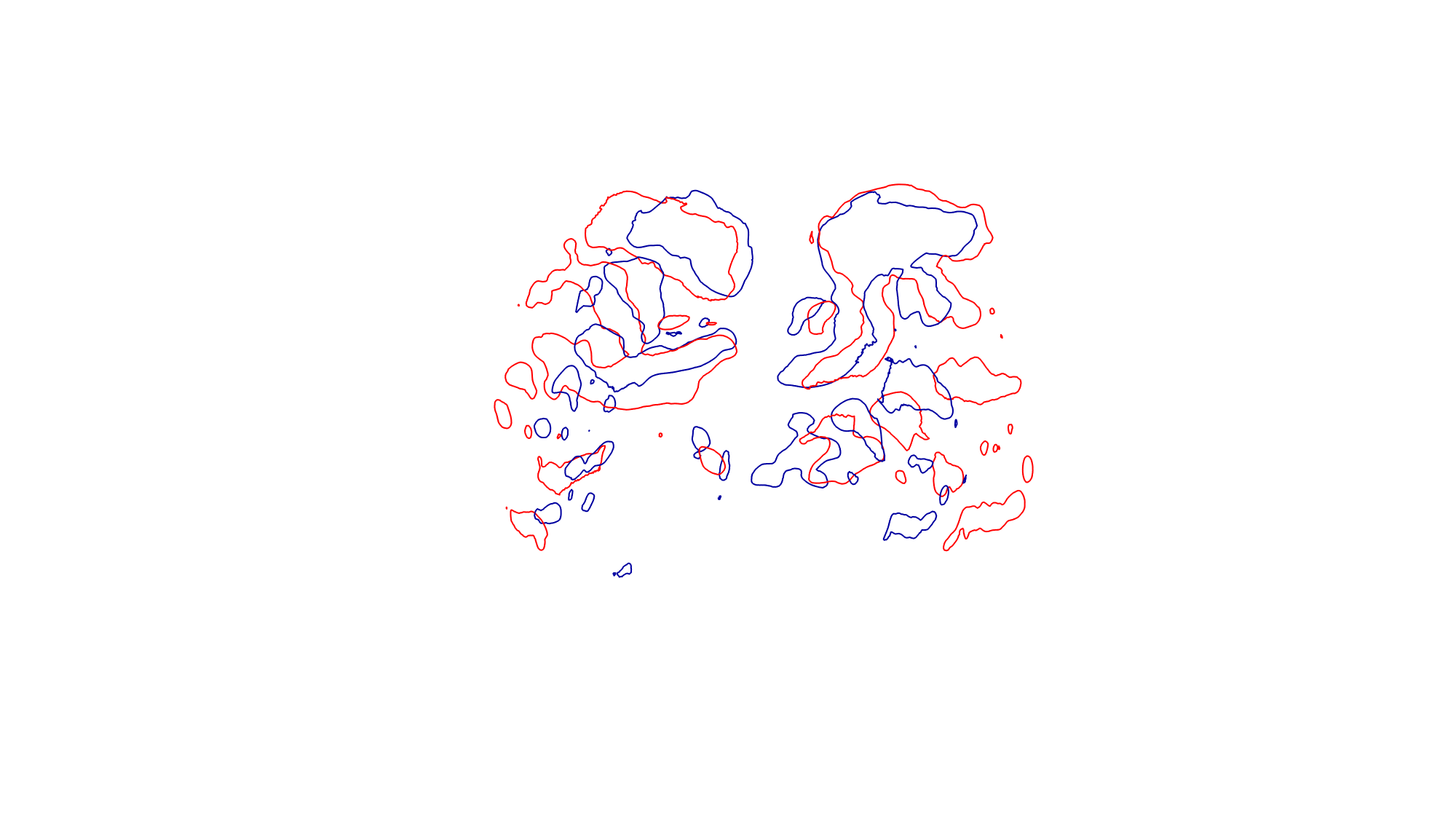 |
| **Ex17 (10 years)** | **Ex18 (11 years)** |
| 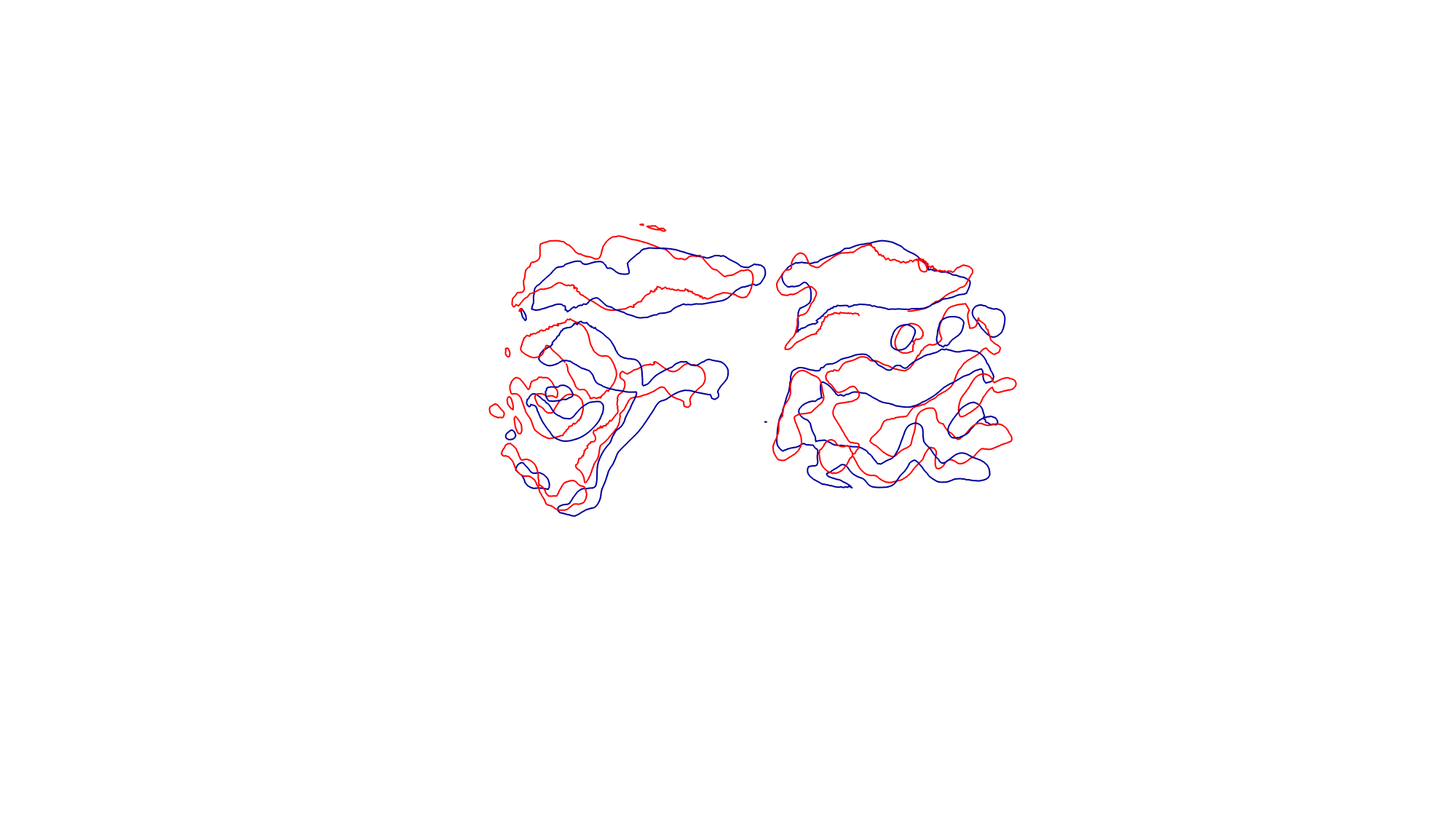 | 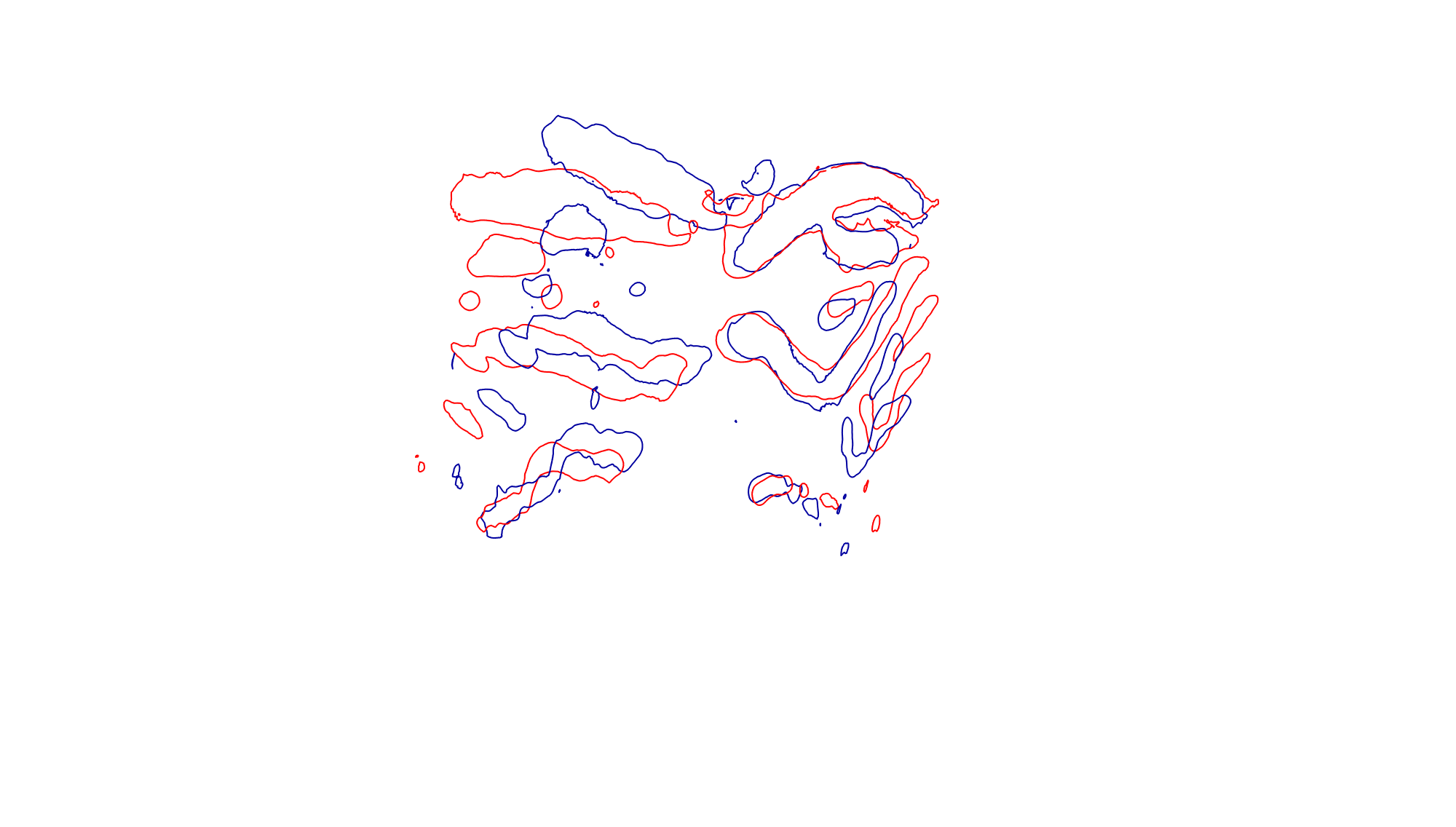 |
| **Ex19 (11 years)** | **Ex20 (10 years)** |
| 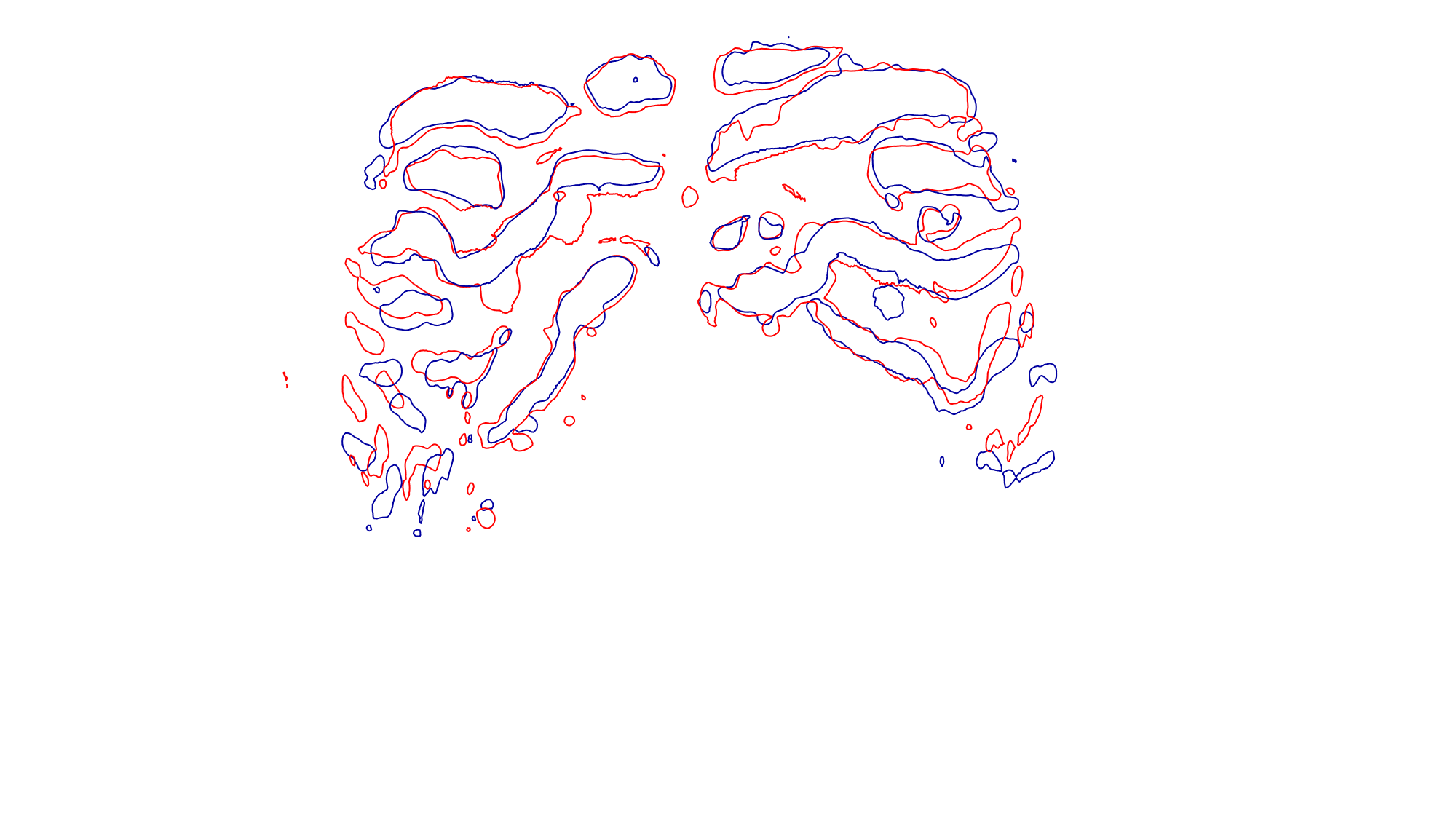 | 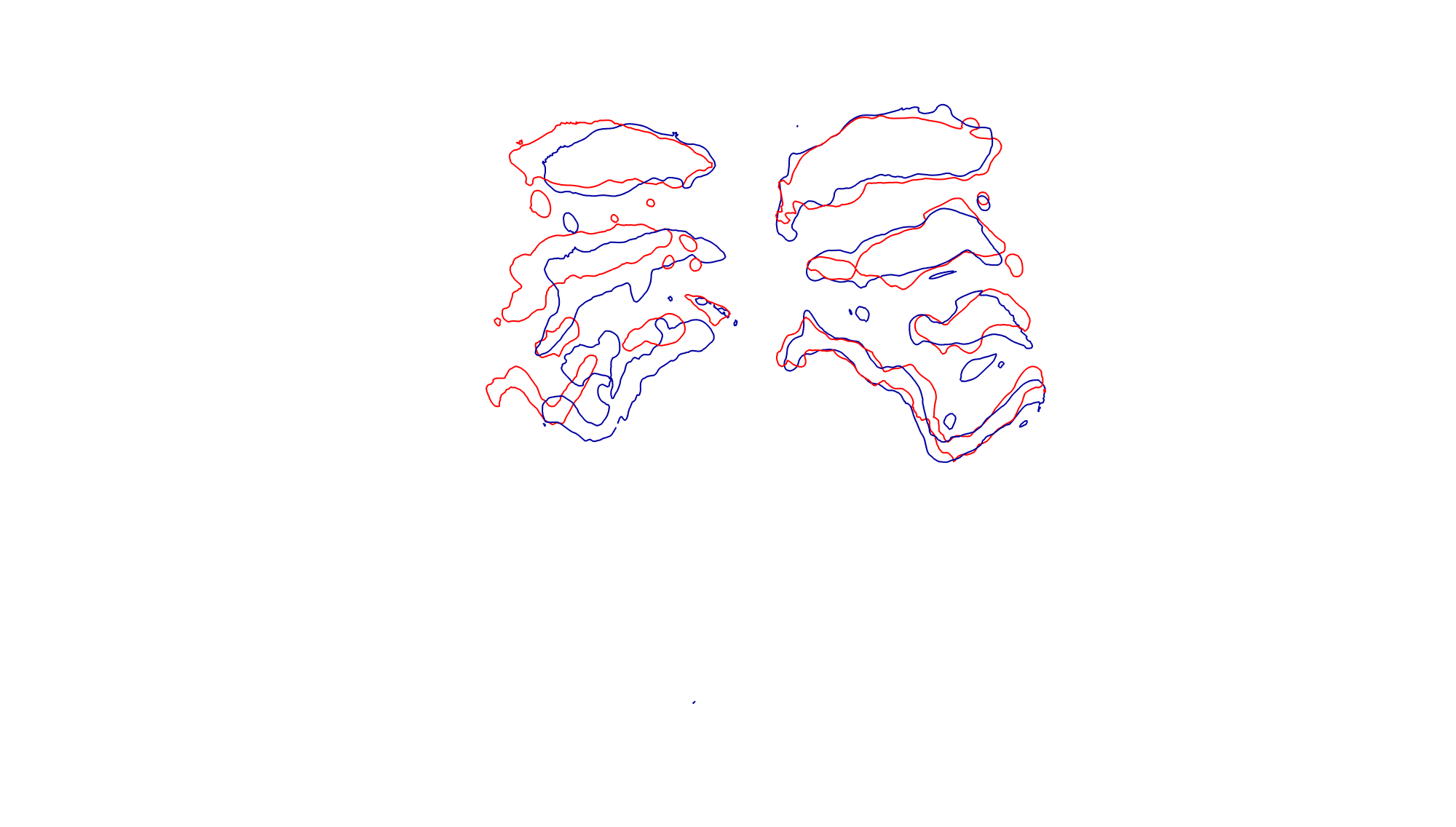 |
| **Ex21 (12 years)** | **Ex22 (12 years)** |
| 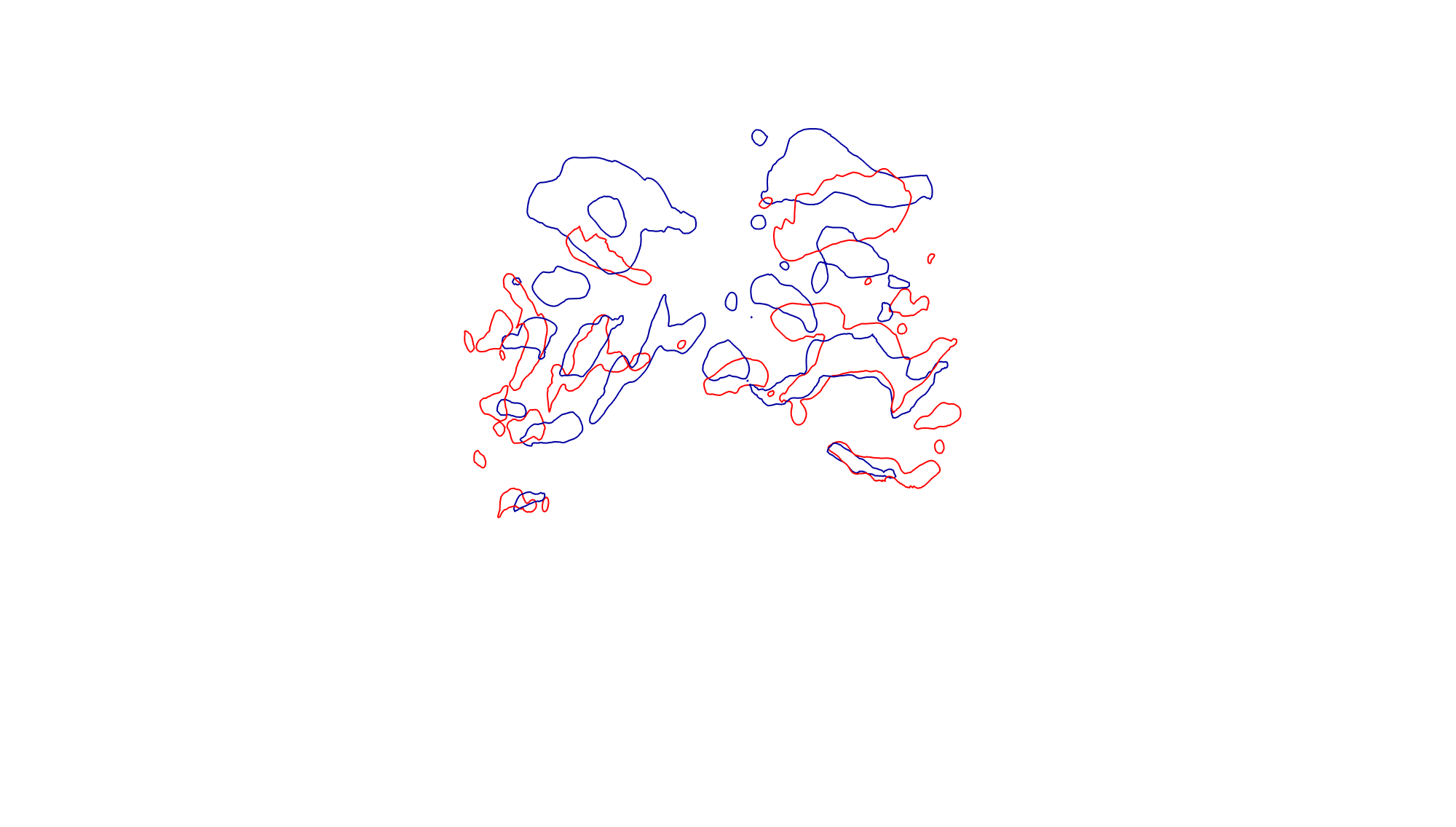 | 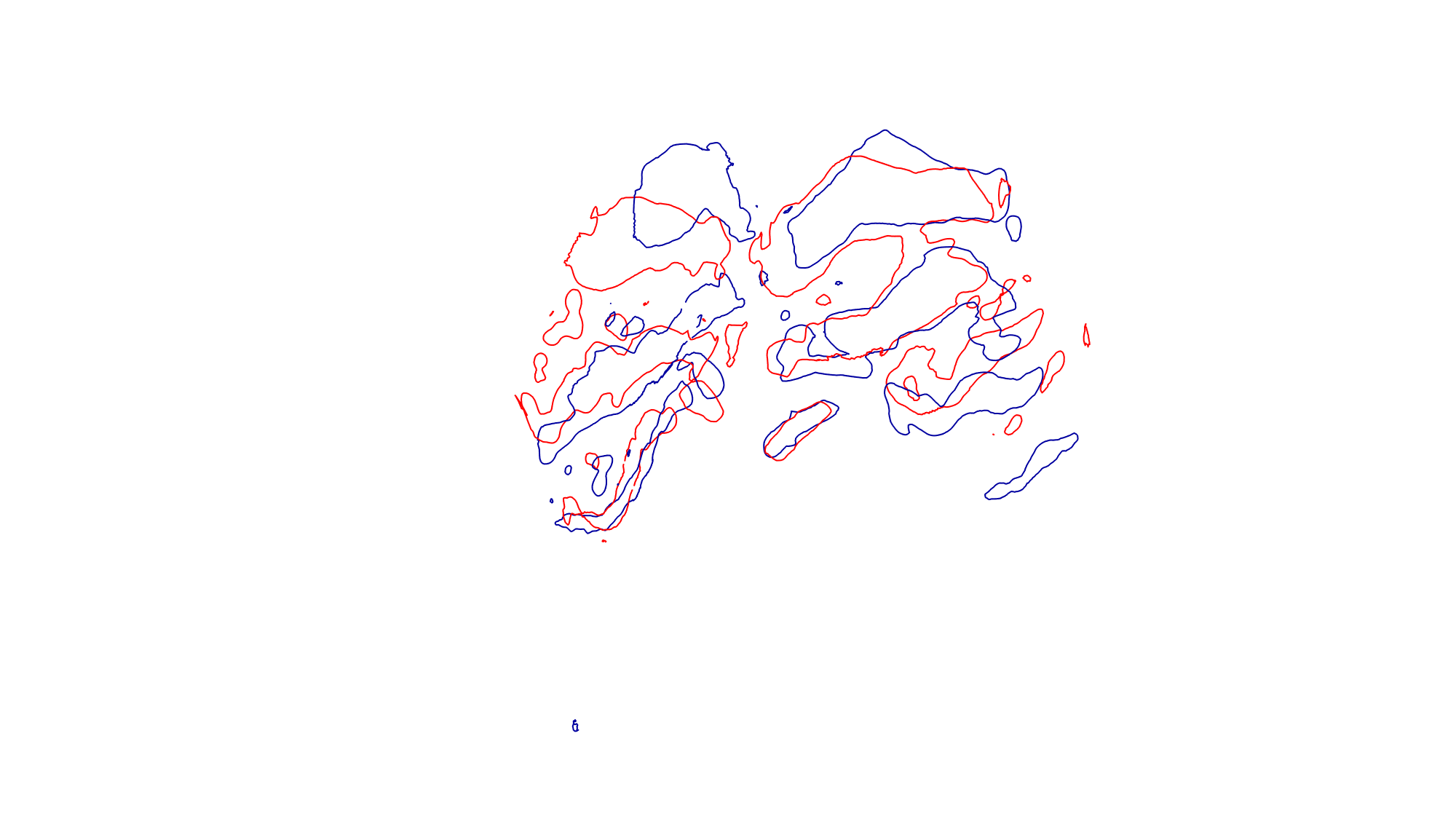 |
| **Ex23 (16 years)** | **Ex24 (18 years)** |
| 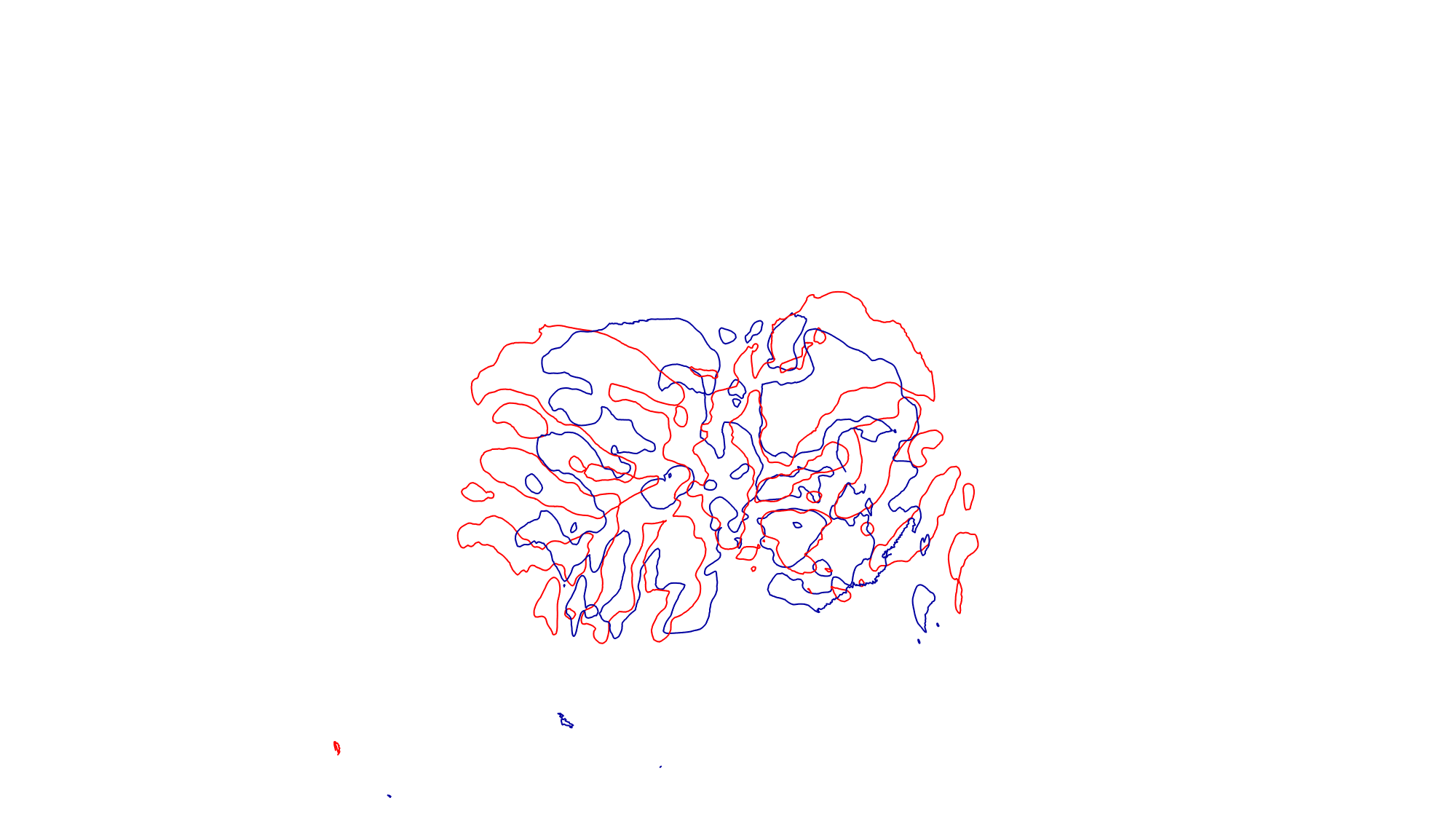 | 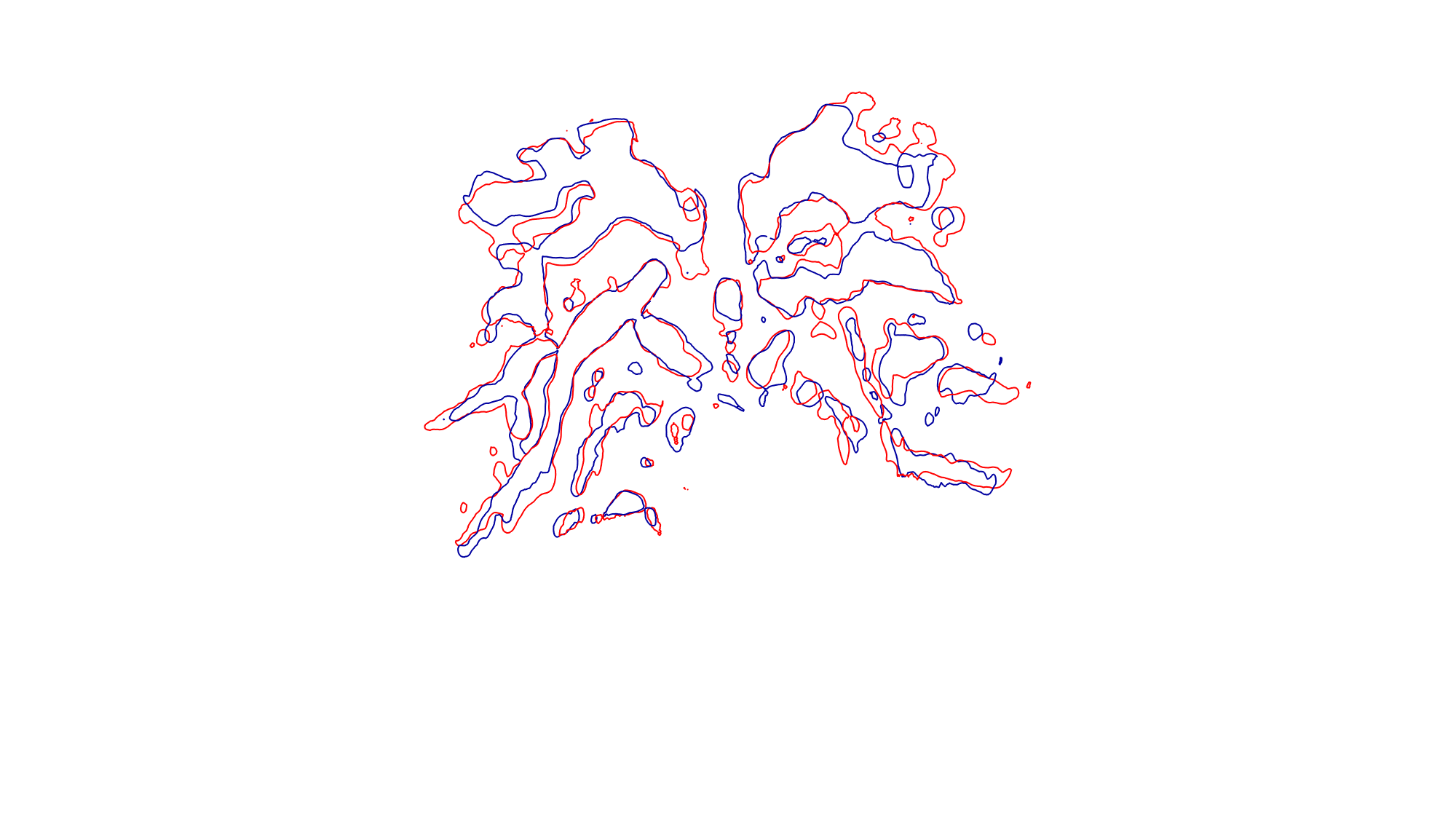 |
| **Ex25 (11 years)** | **Ex26 (10 years)** |
| 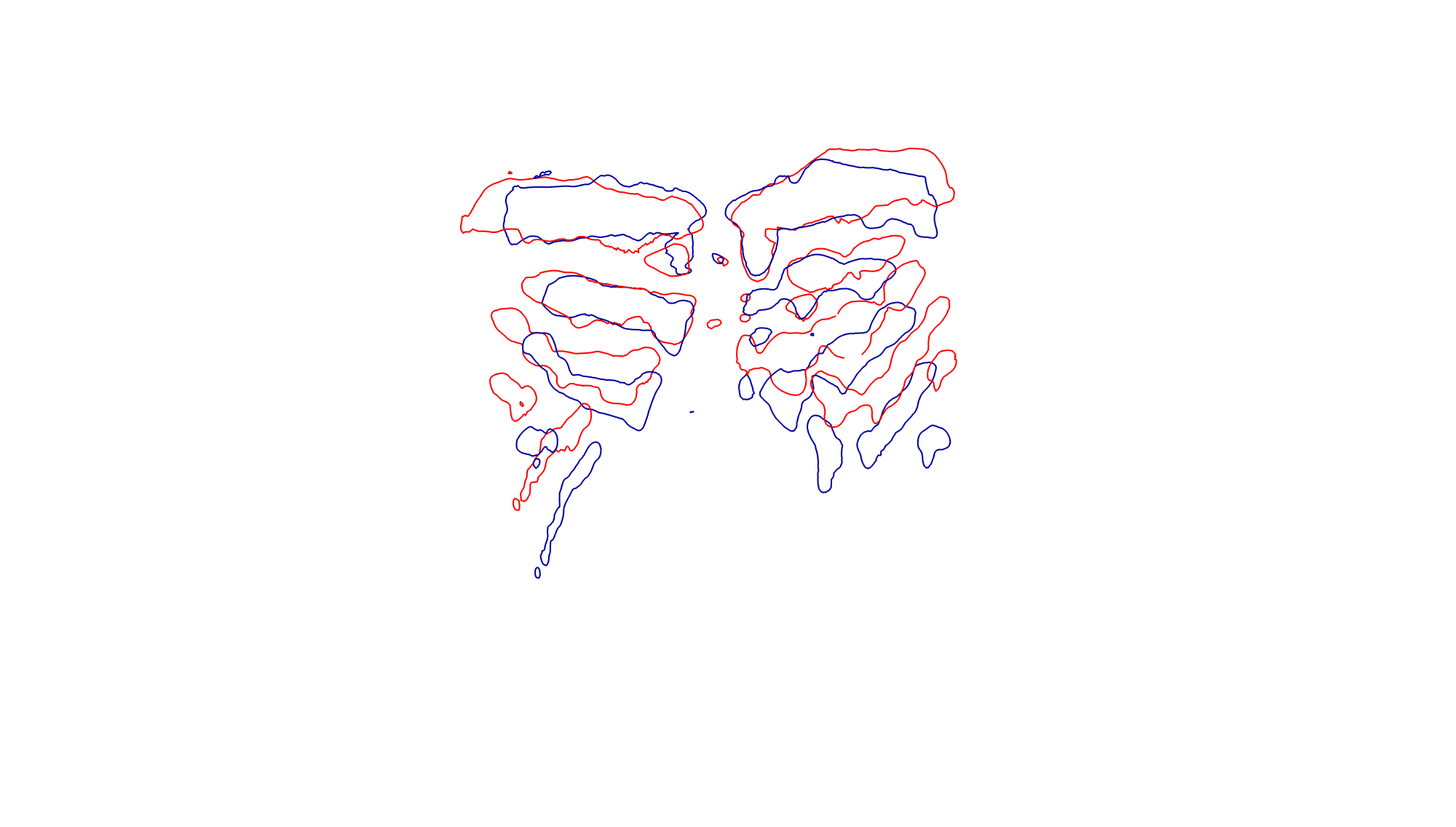 | 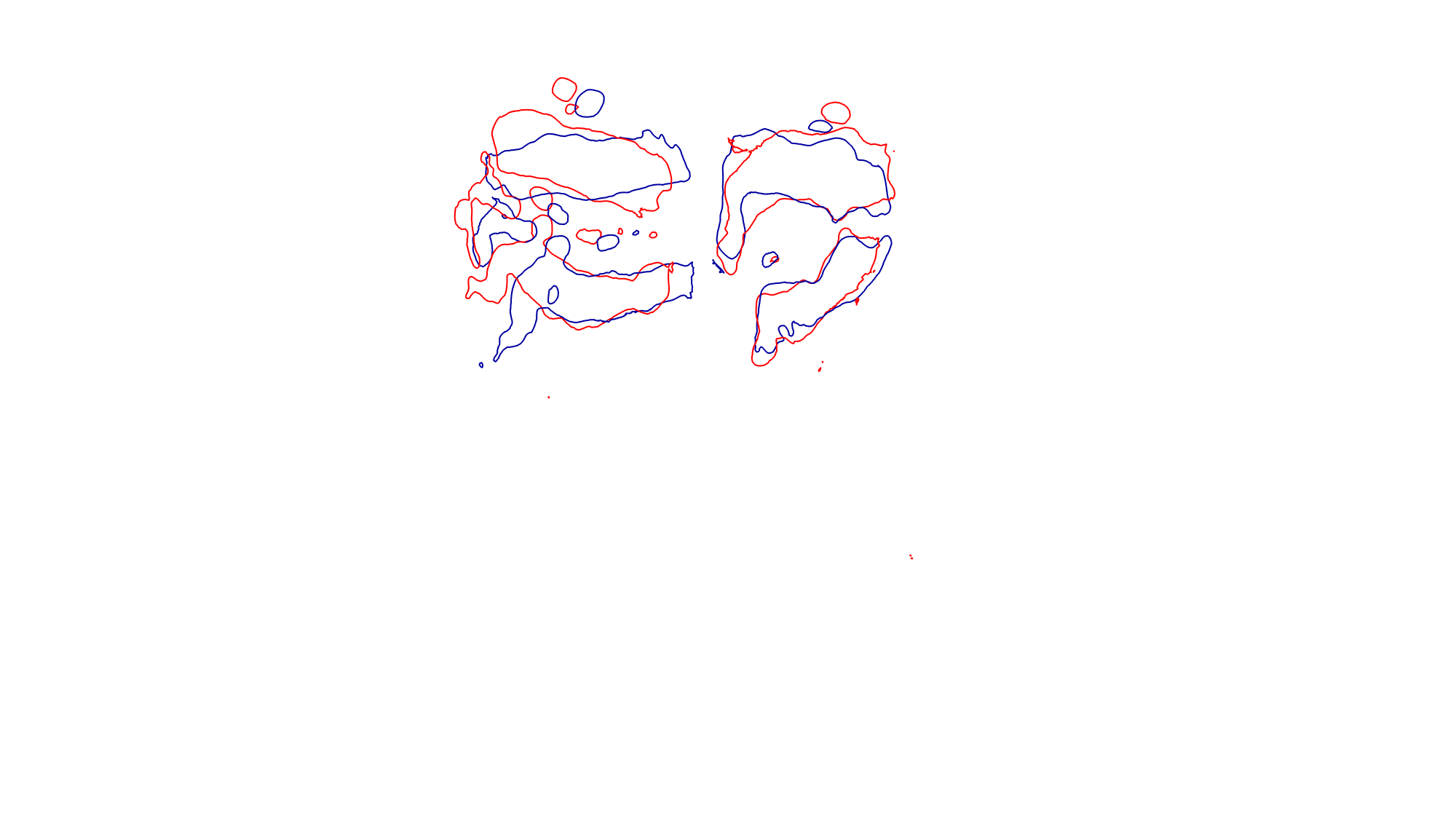 |
| **Ex27 (13 years)** | **Εx28 (11 years)** |
| 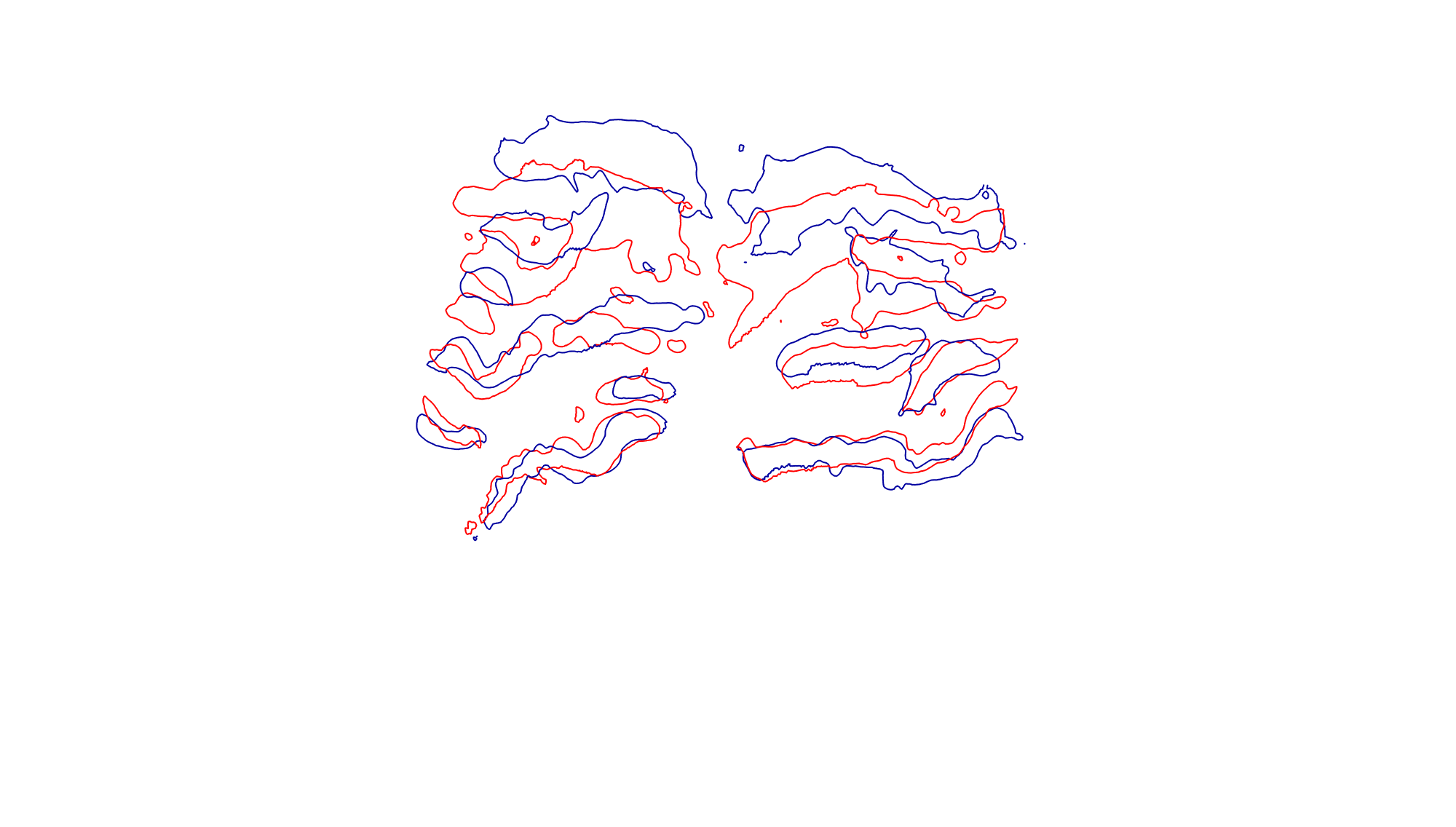 | 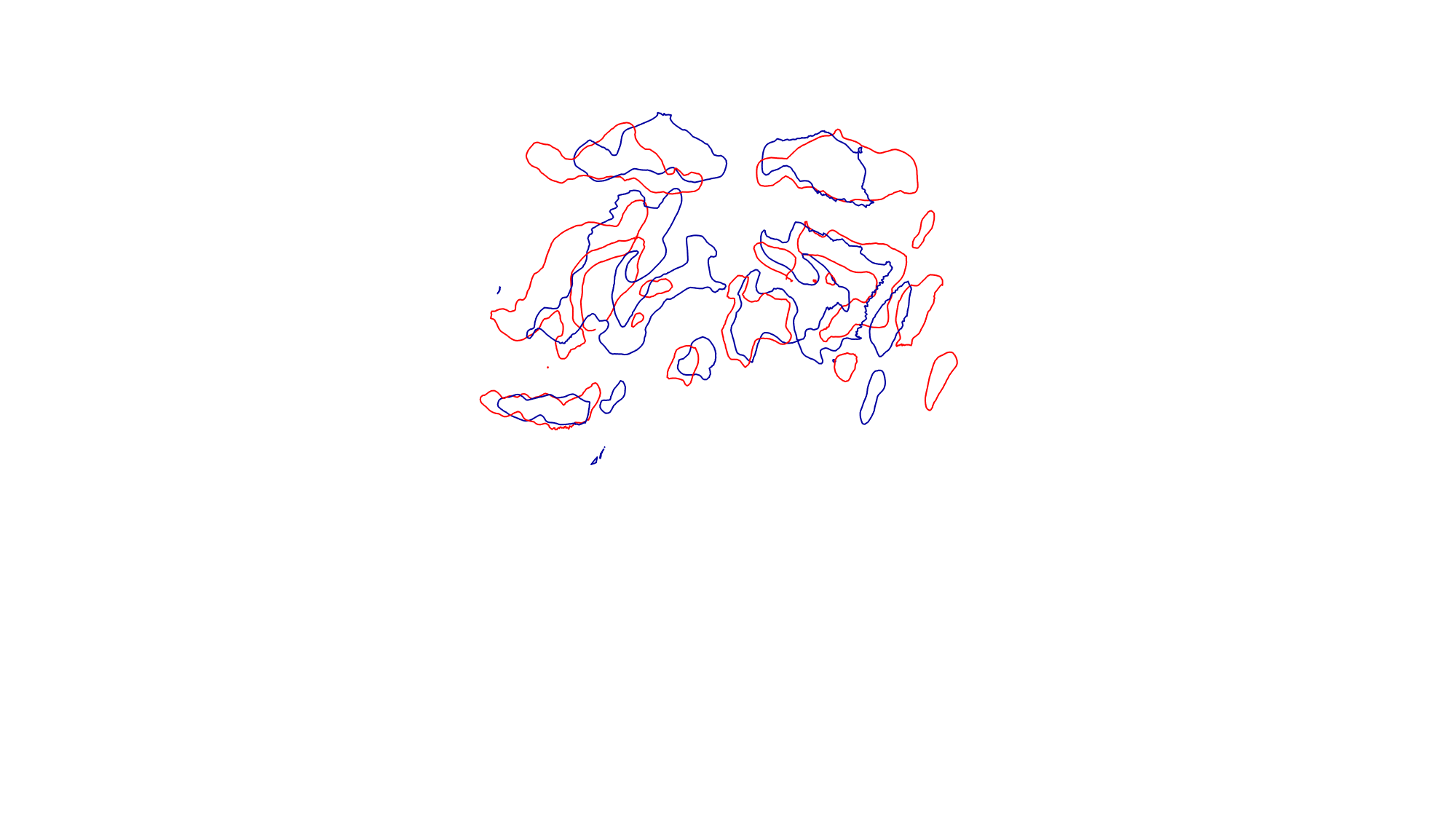 |

**Supplementary figure 2:** Screenshots of 3D best-fit superimposition of pre- (blue) and post-treatment (red) palatal rugae for the non- extraction group with age at the beginning of treatment in parenthesis.

| **Nonex01 (11 years)** | **Nonex02 (17 years)** |
| --- | --- |
| 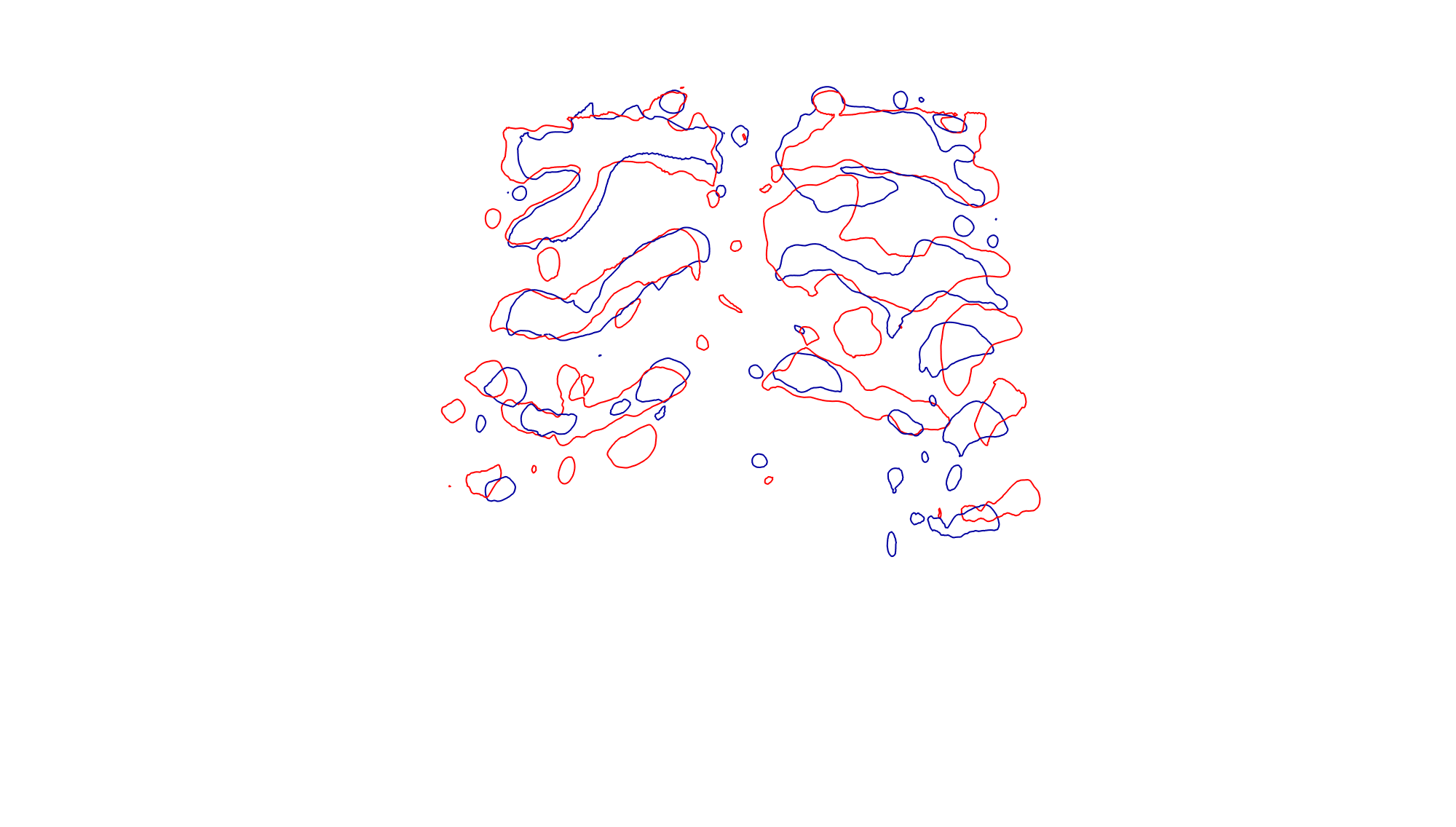 | 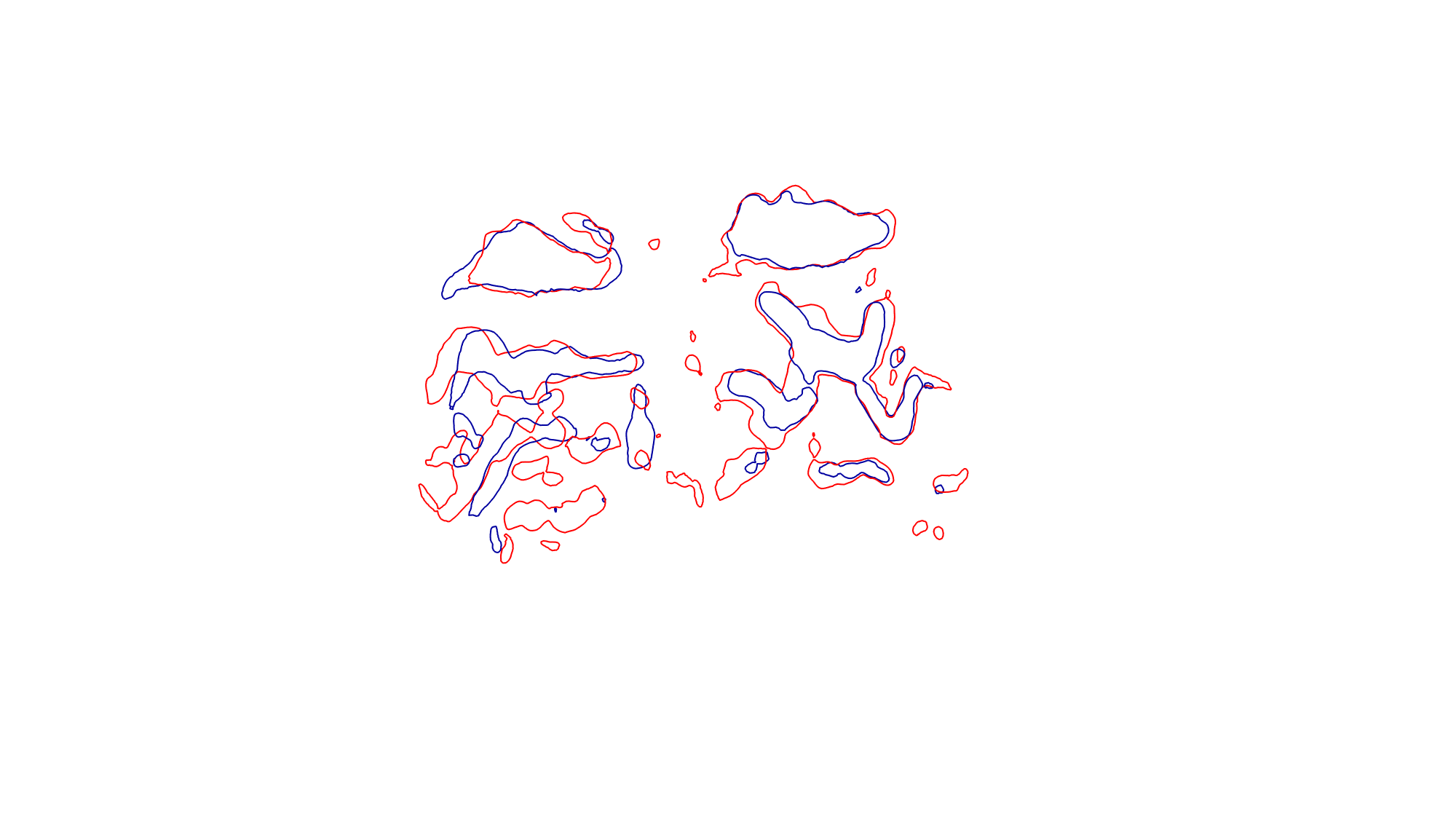 |
| **Nonex03 (20 years)** | **Nonex04 (15 years)** |
| 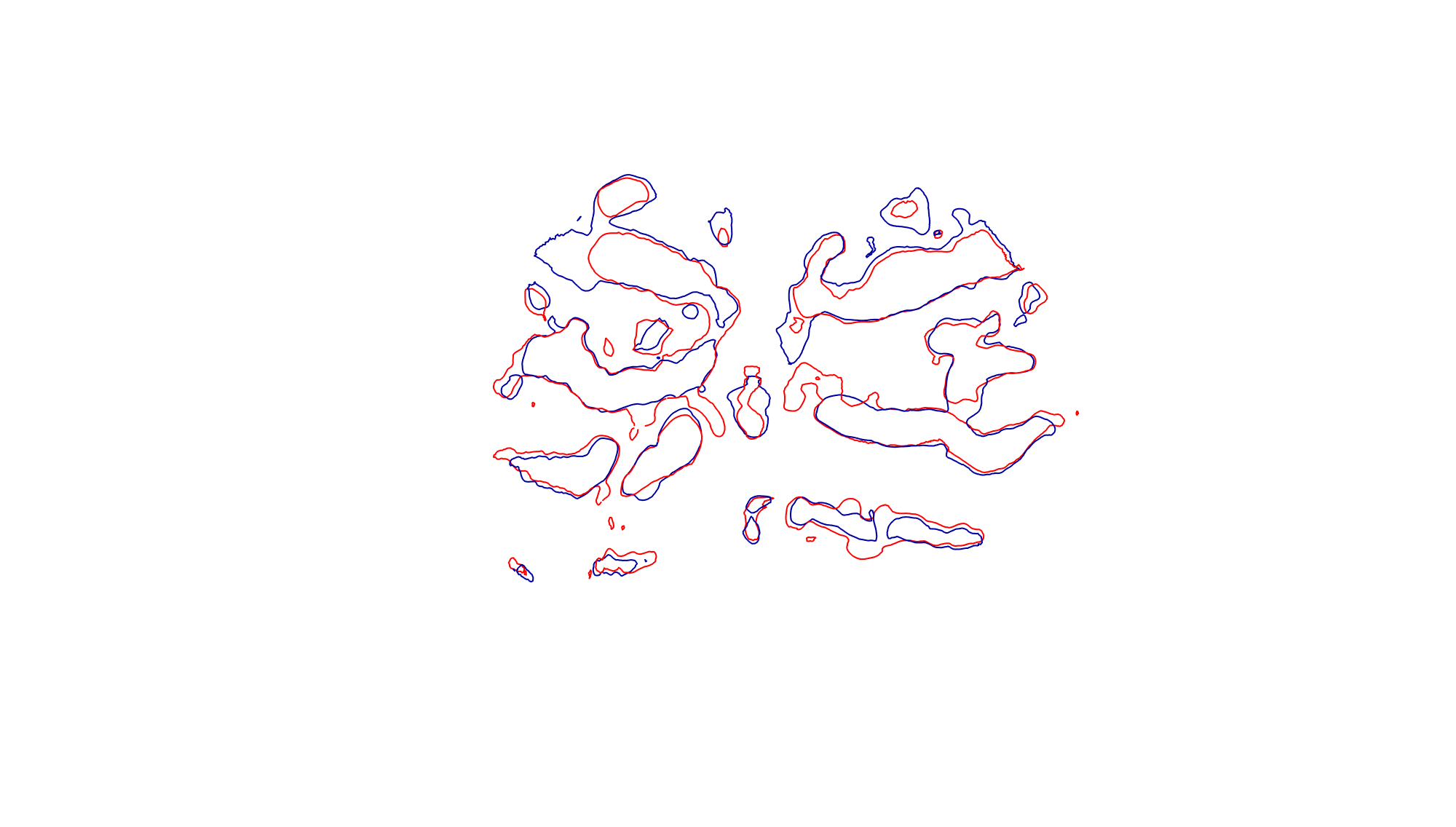 | 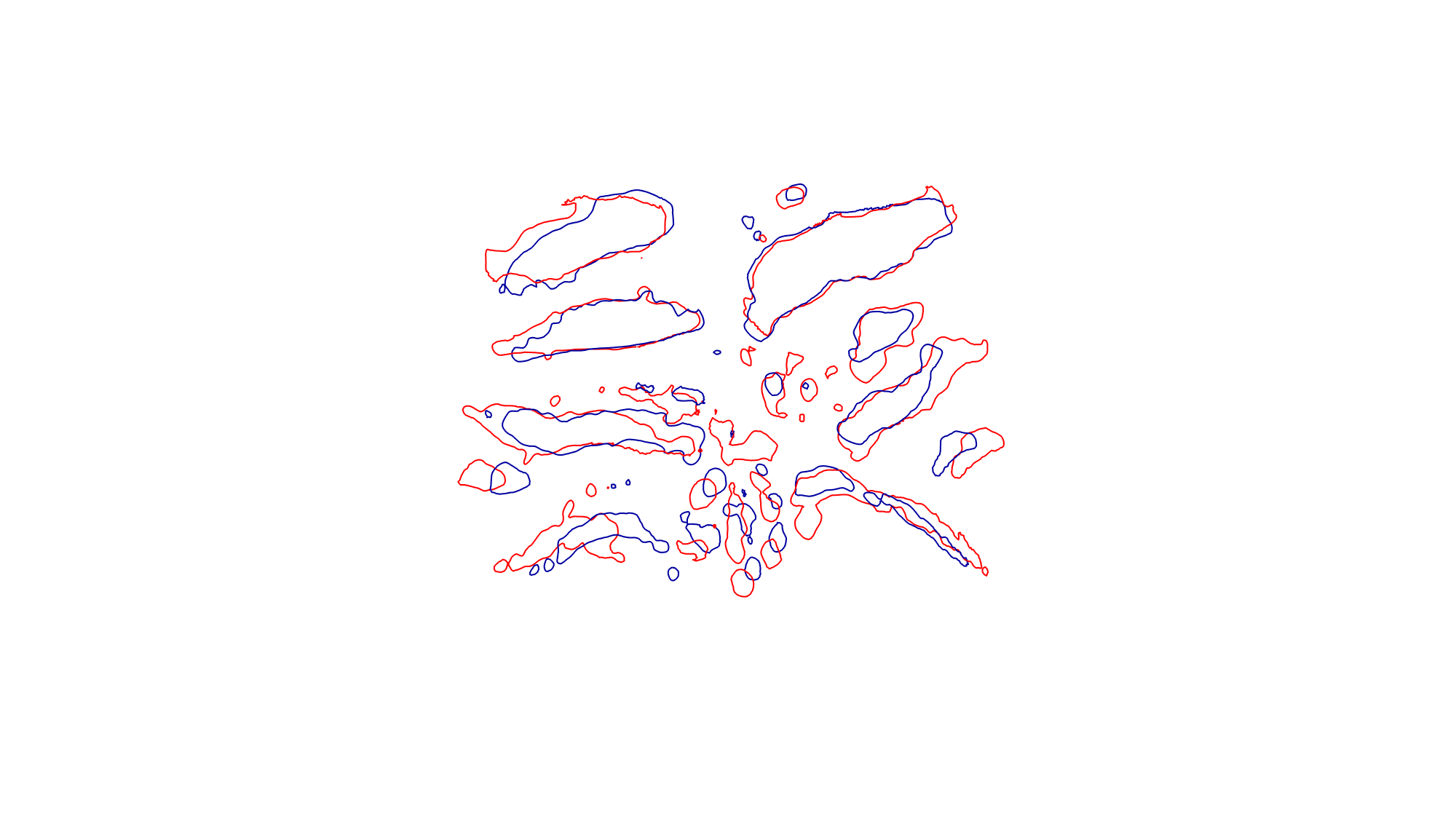 |
| **Nonex05 (29 years)** | **Nonex06 (10 years)** |
| 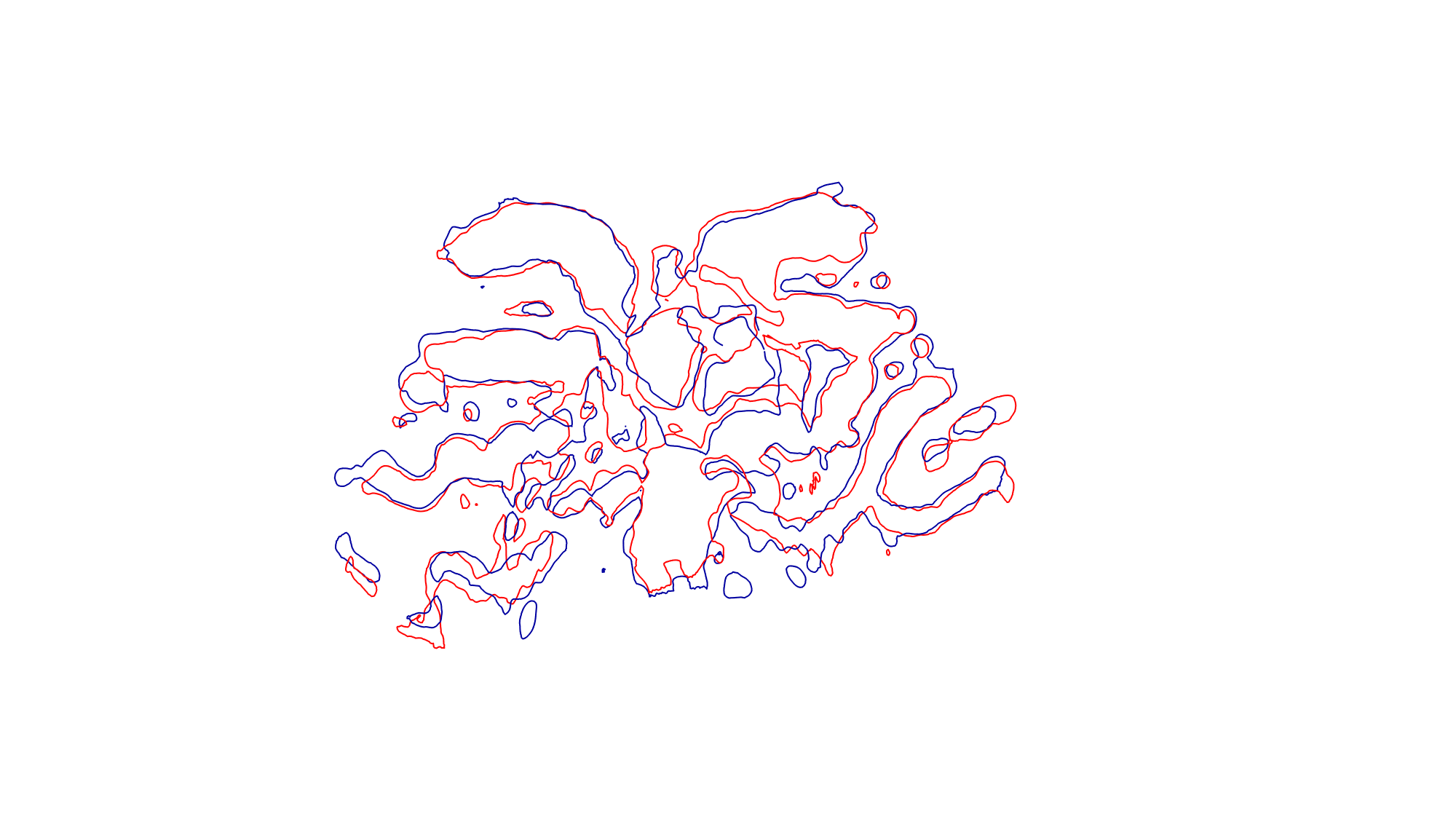 | 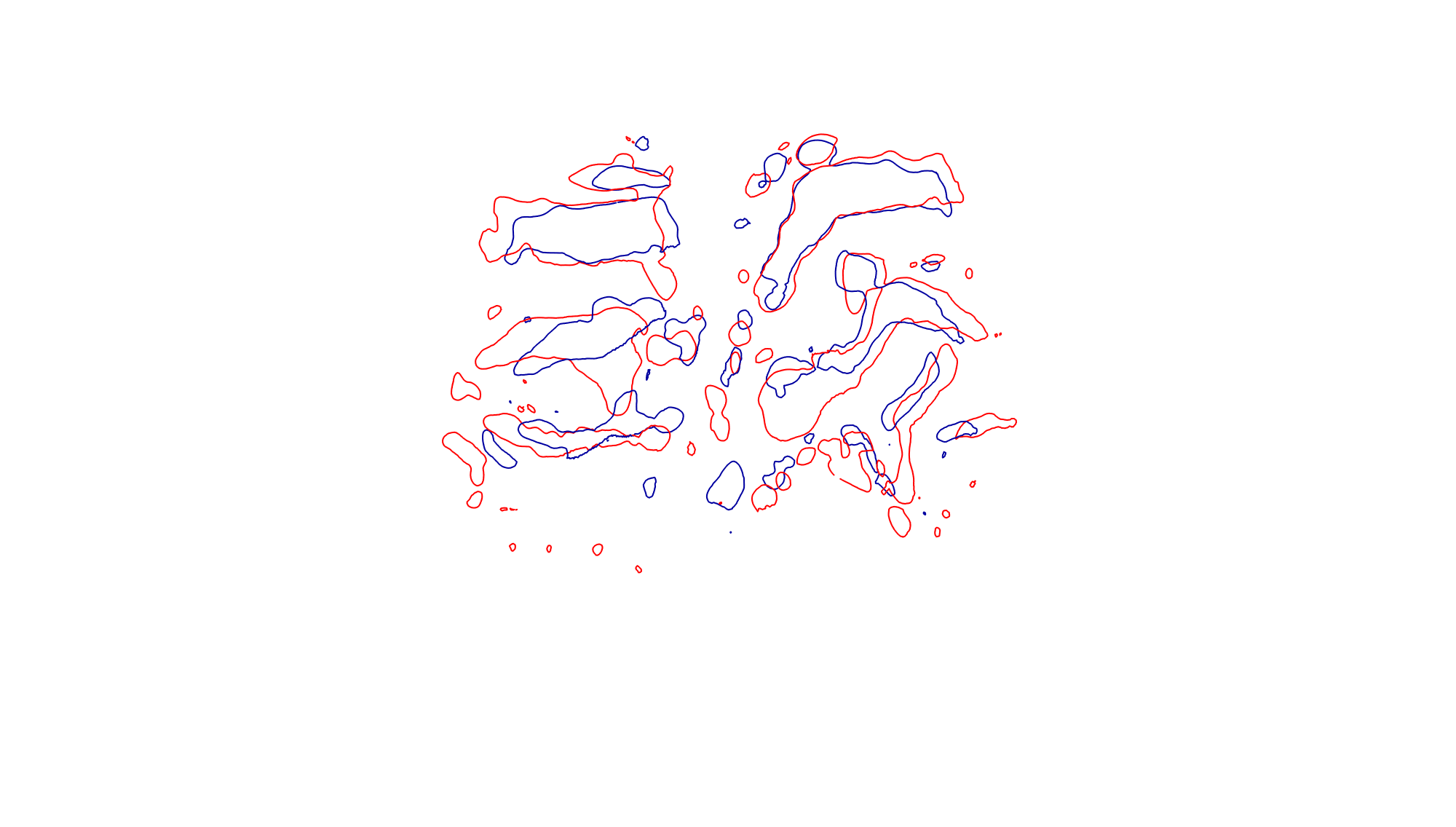 |
| **Nonex07 (14 years)** | **Nonex08 (23 years)** |
| 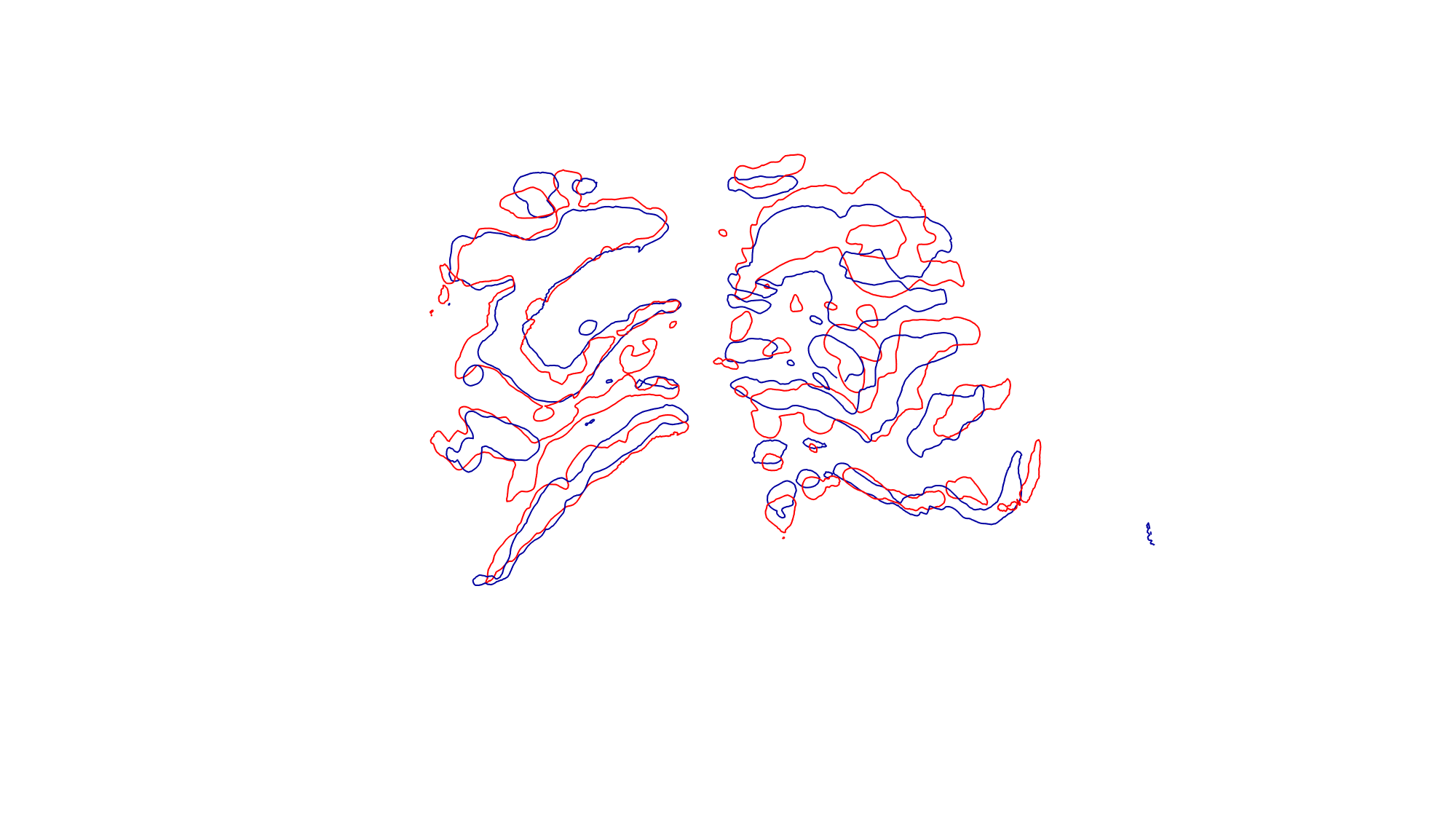 | 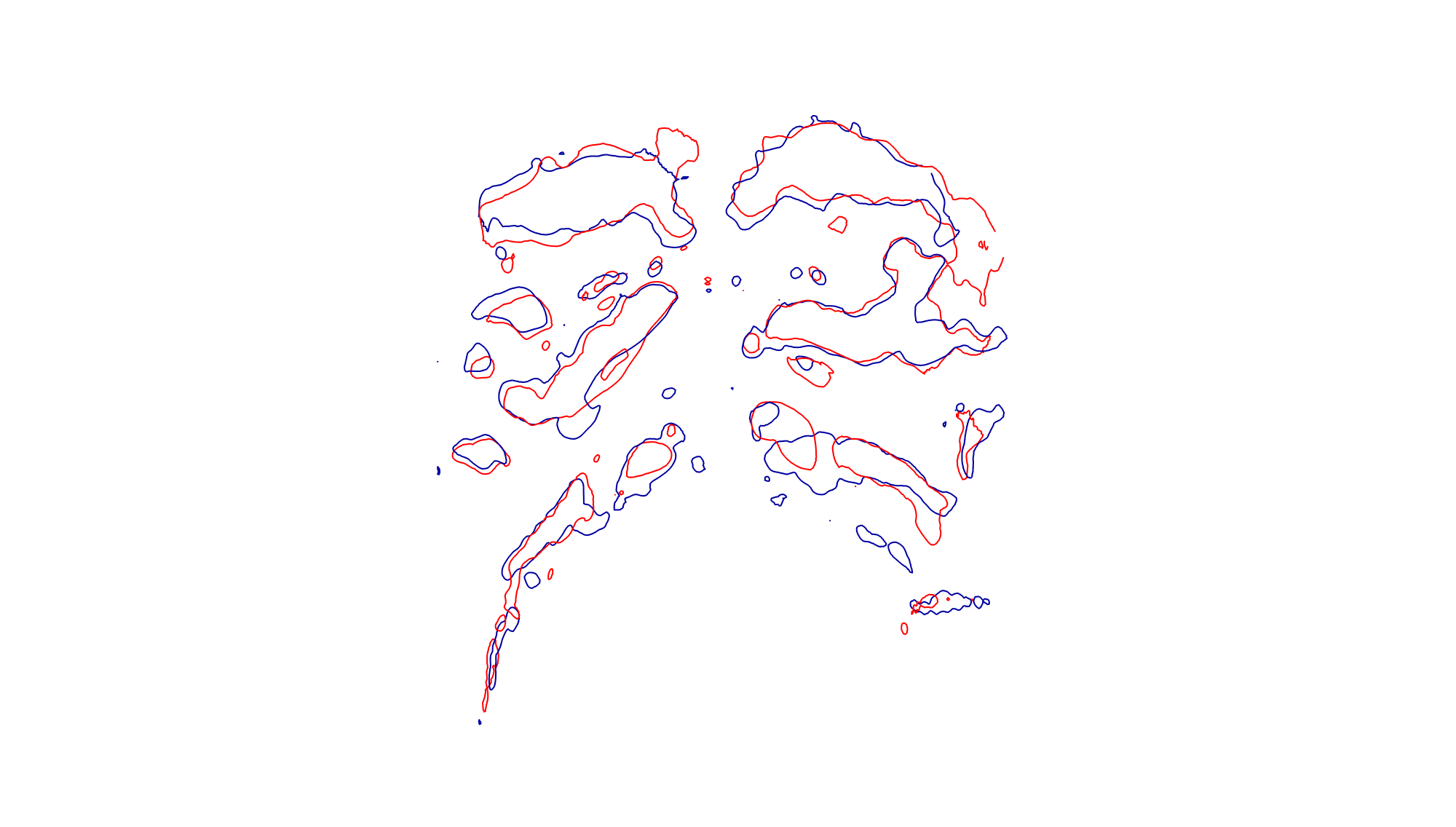 |
| **Nonex09 (10 years)** | **Nonex10 (11 years)** |
| 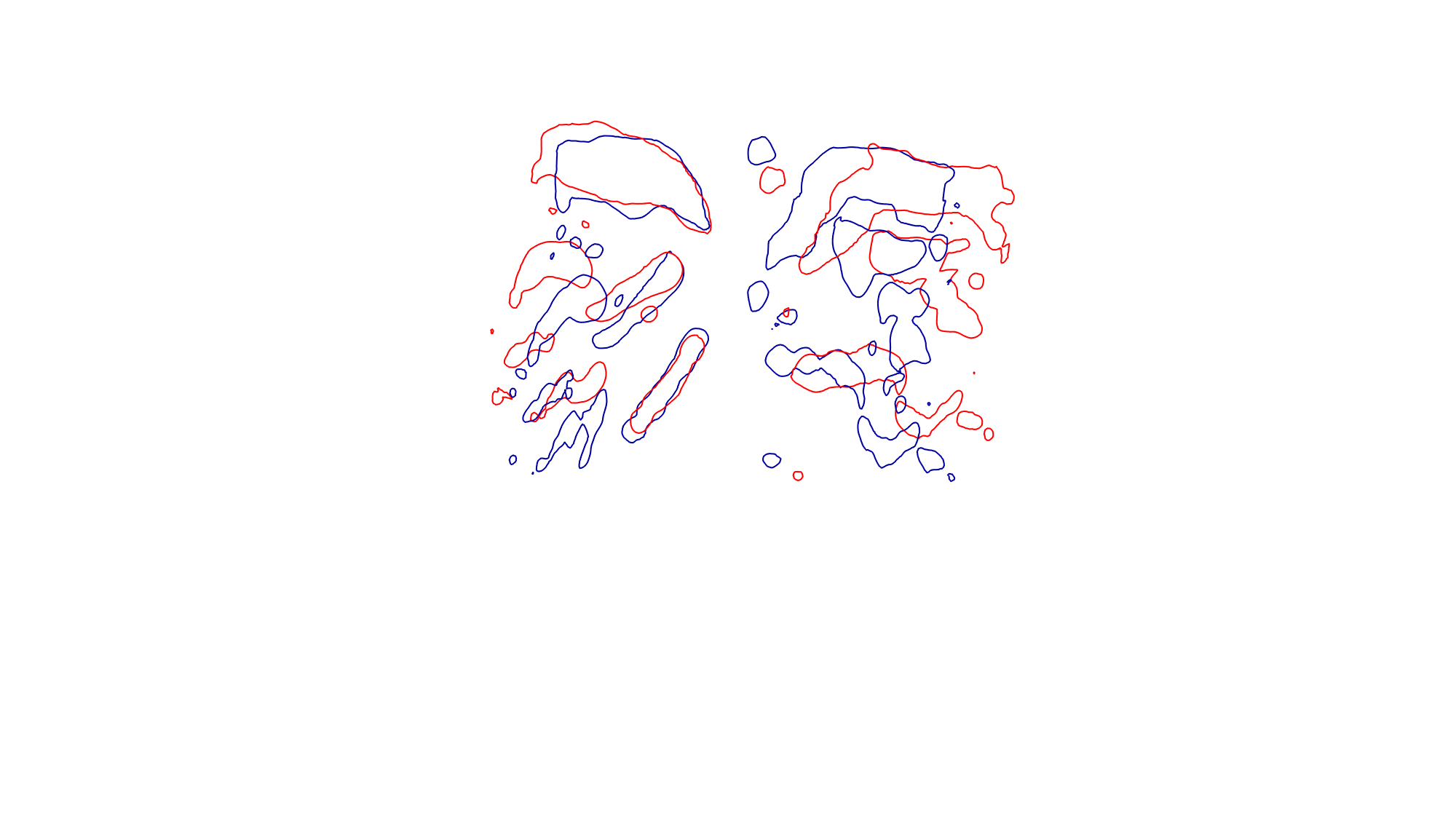 | 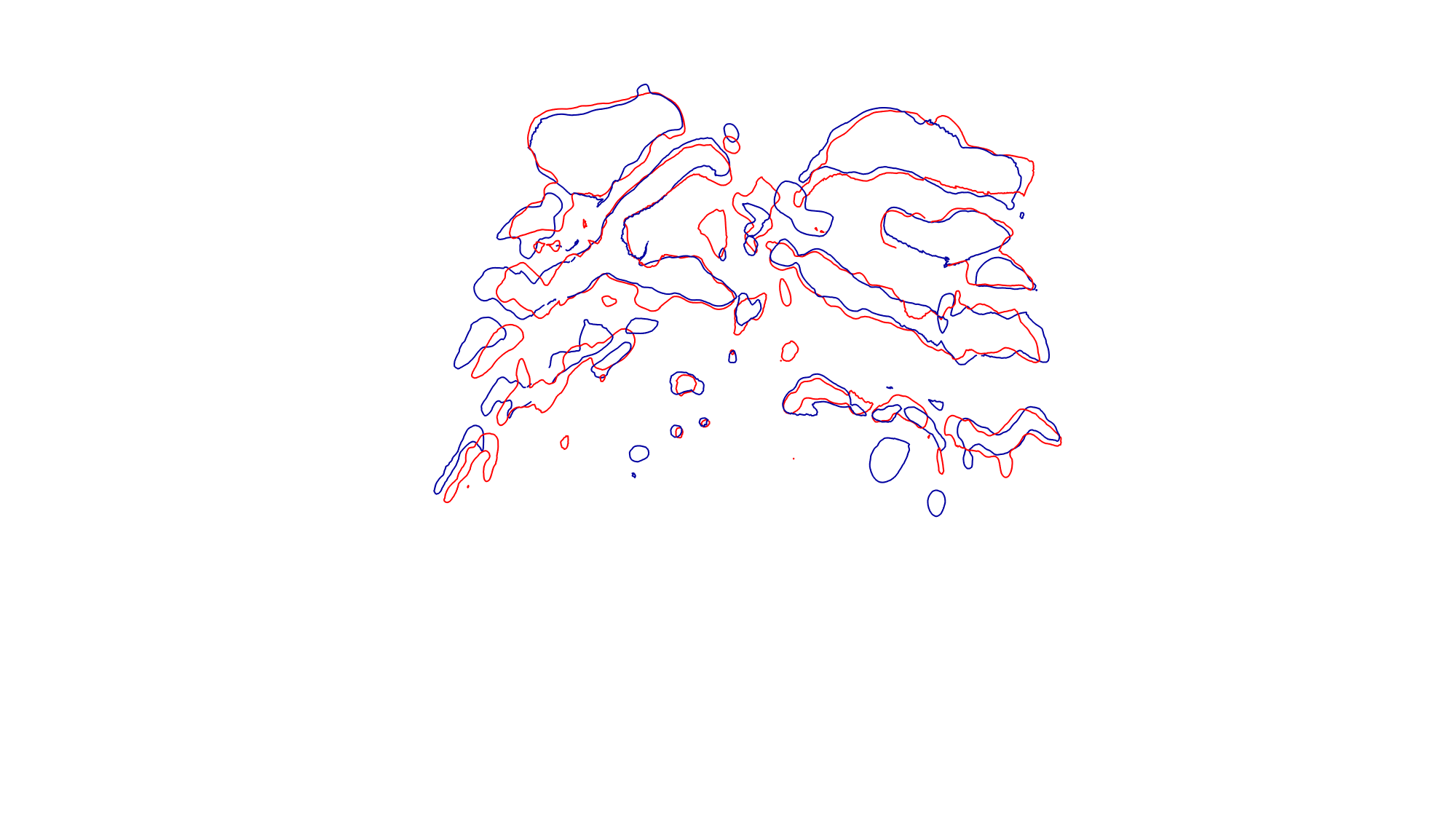 |
| **Nonex11 (15 years)** | **Nonex12 (20 years)** |
| 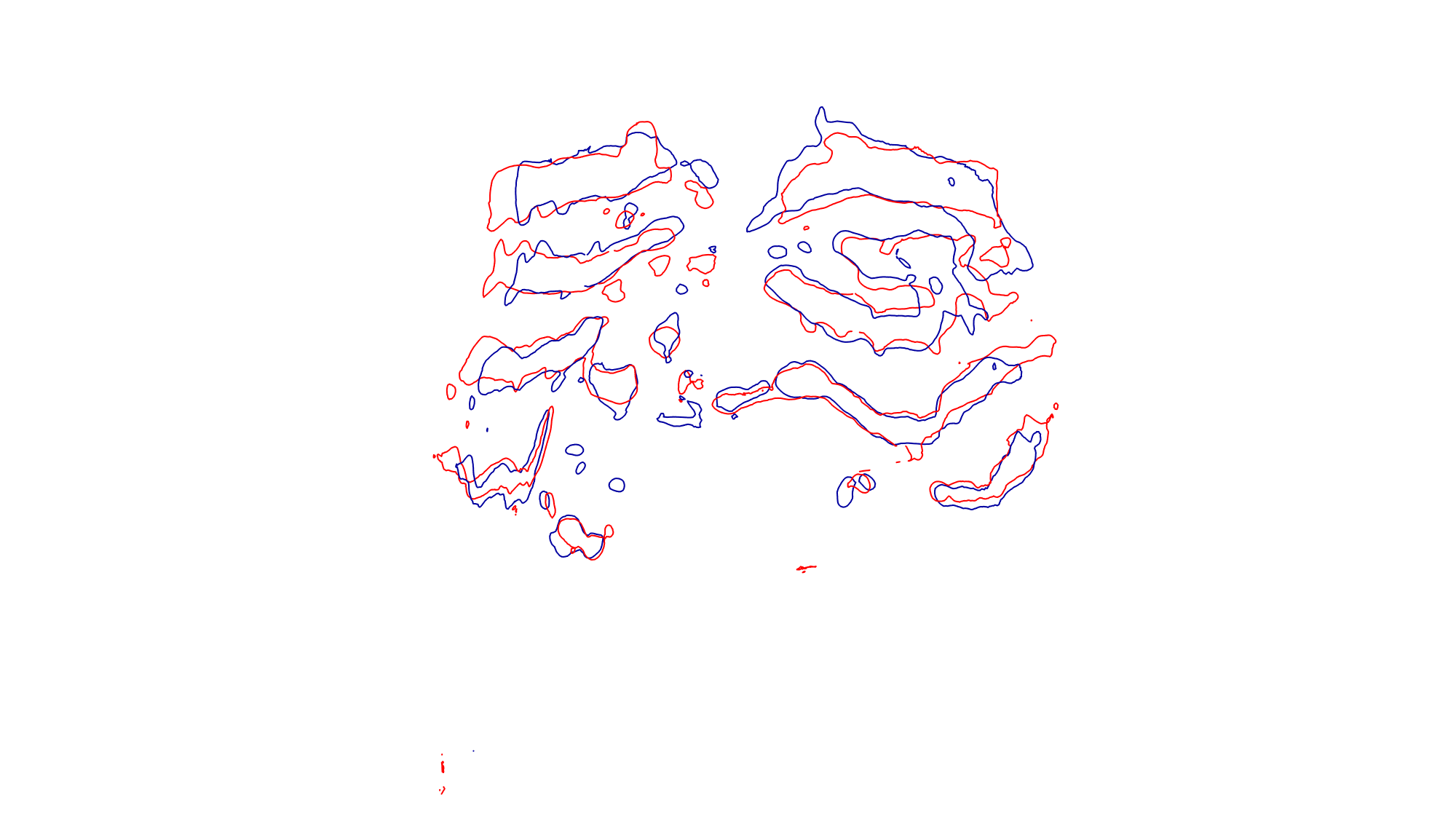 | 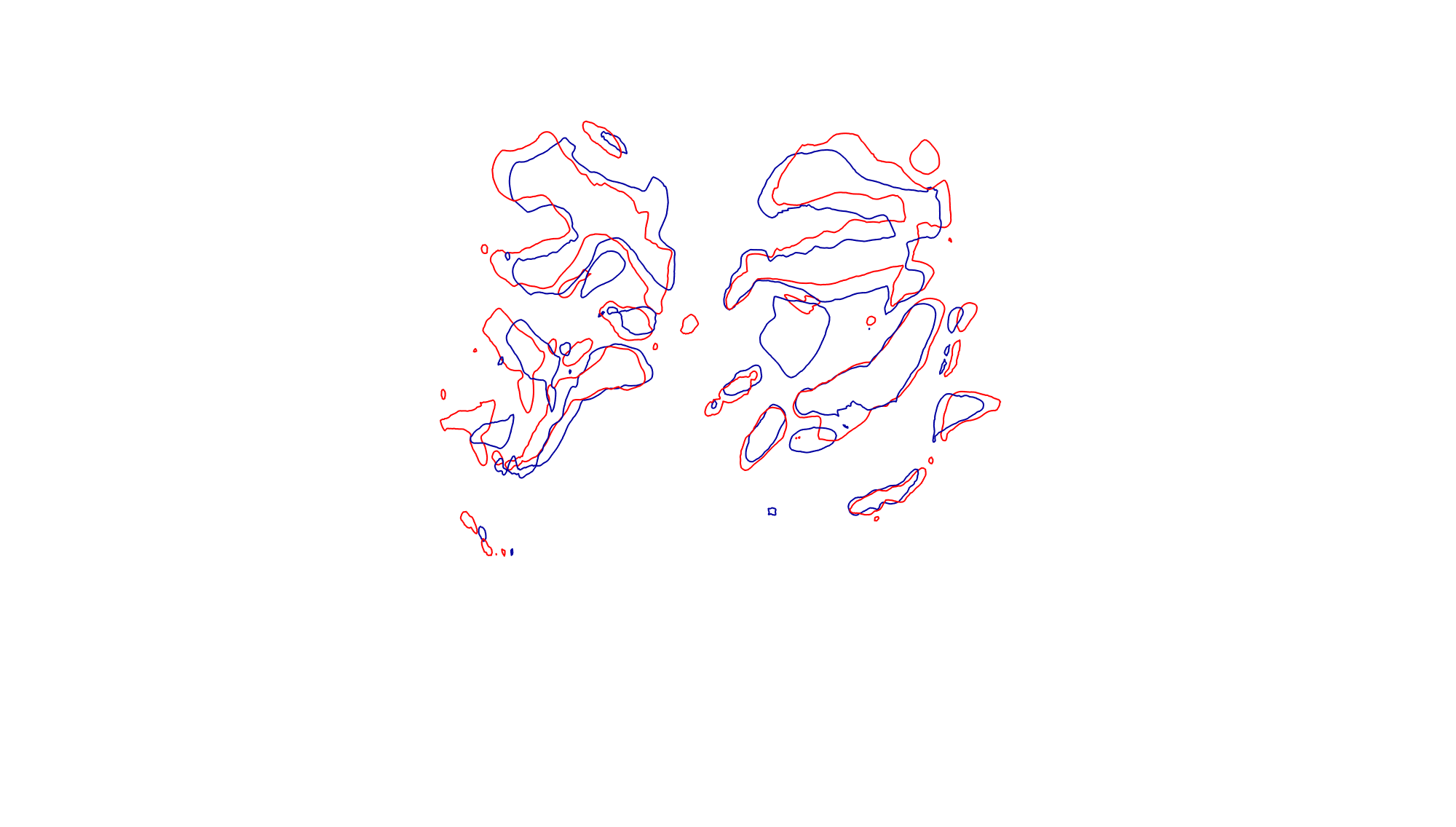 |
| **Nonex13 (12 years)** | **Nonex14 (12 years)** |
| 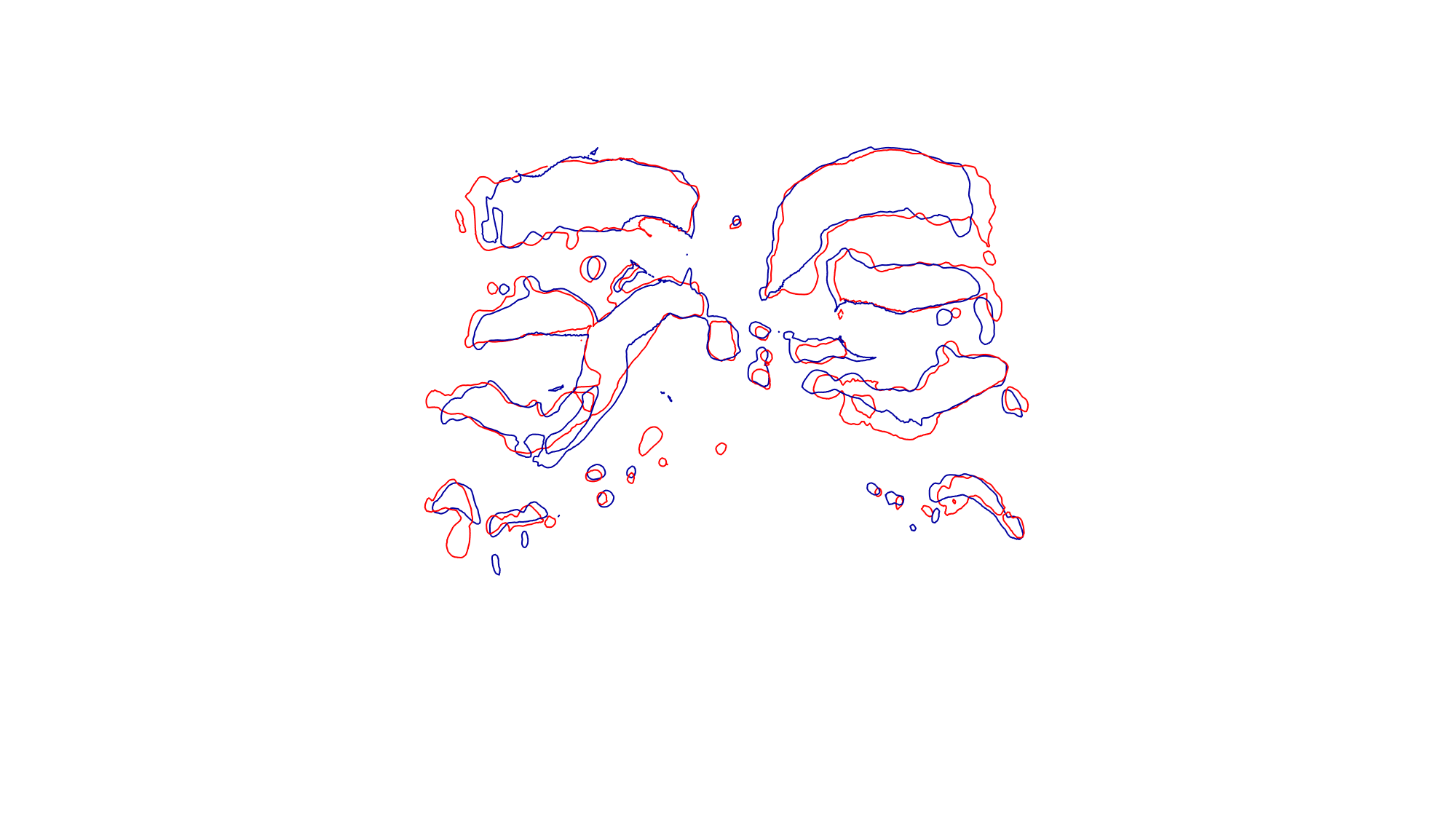 | 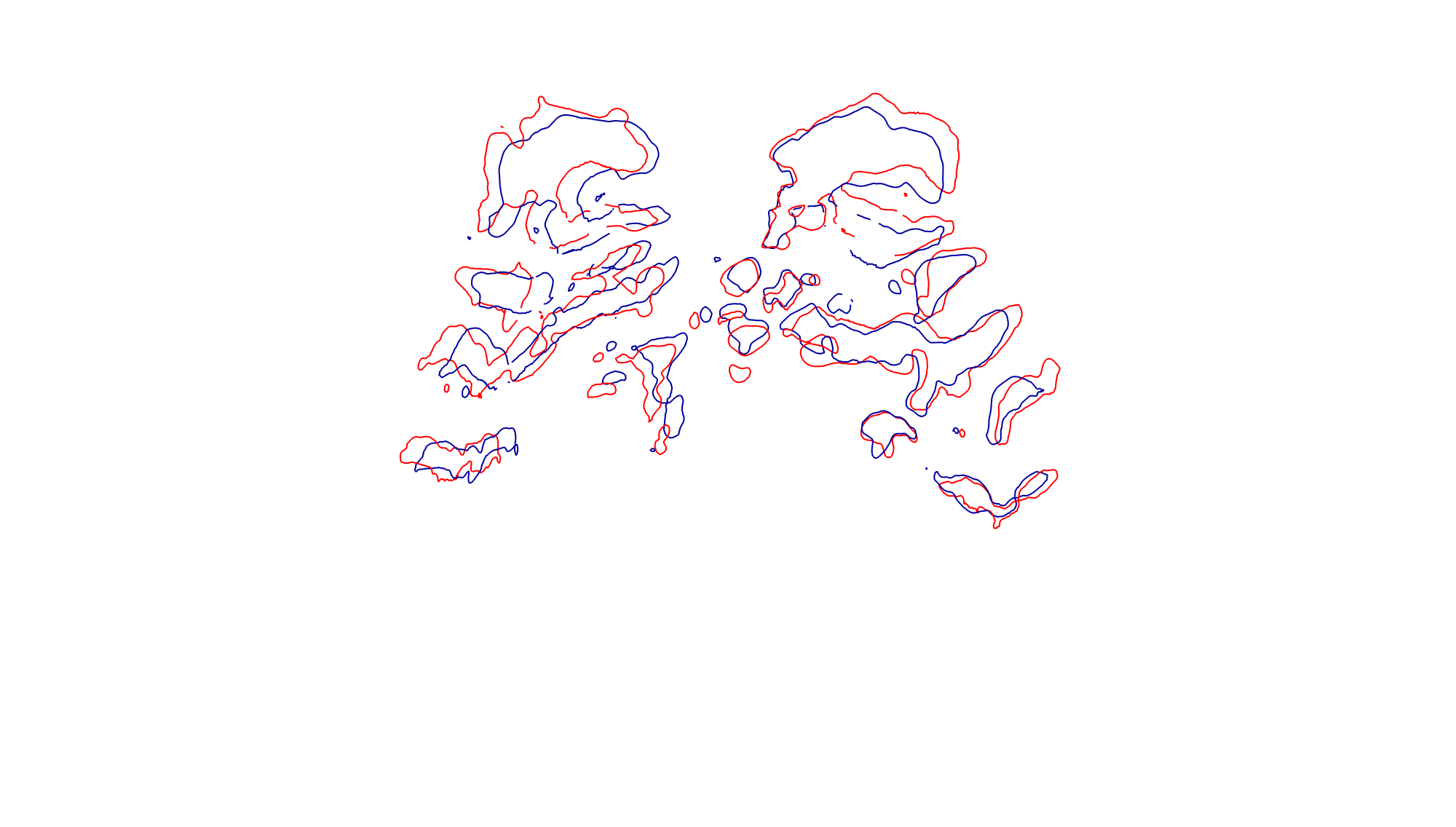 |
| **Nonex15 (11 years)** | **Nonex16 (14 years)** |
| 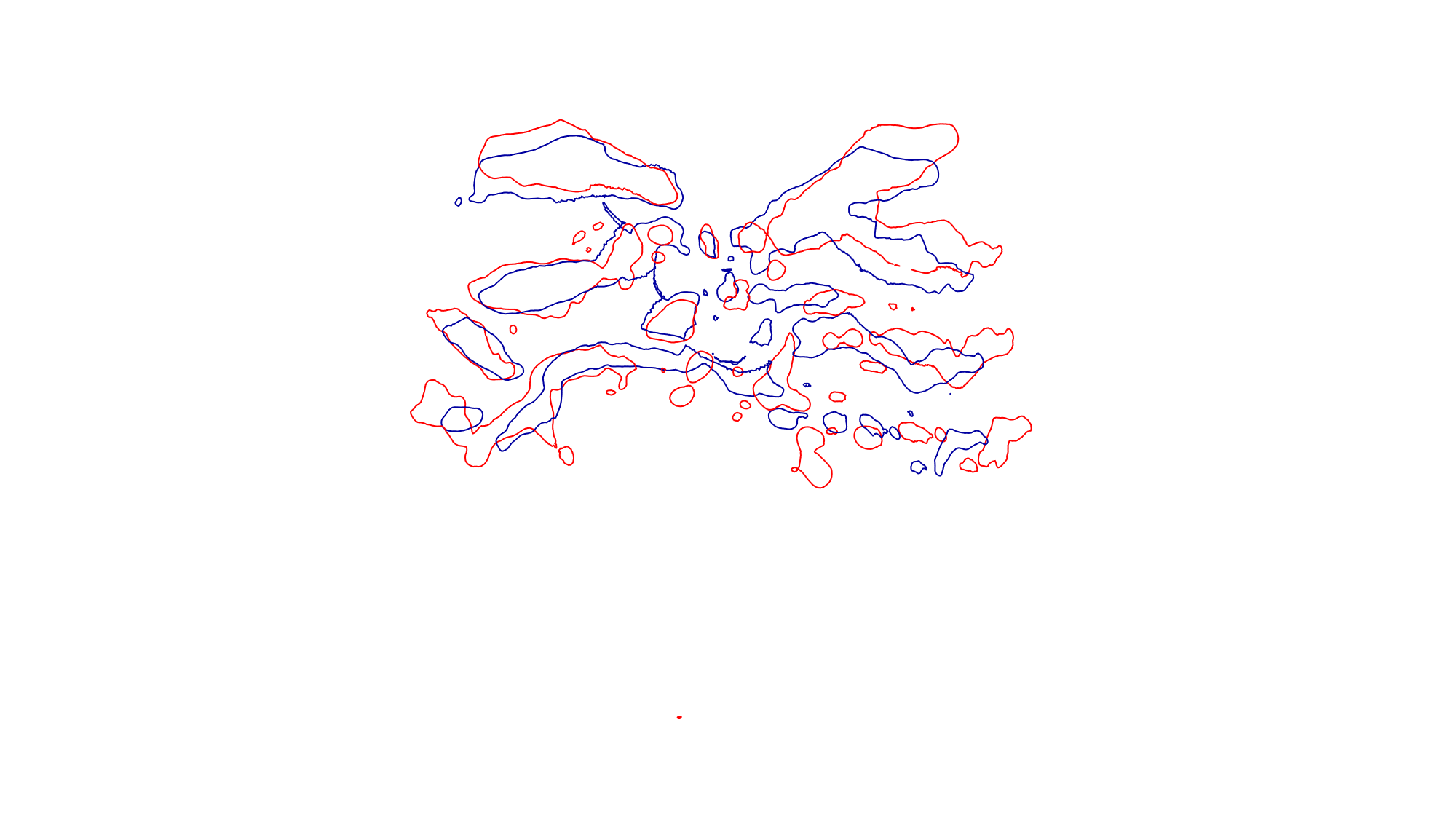 | 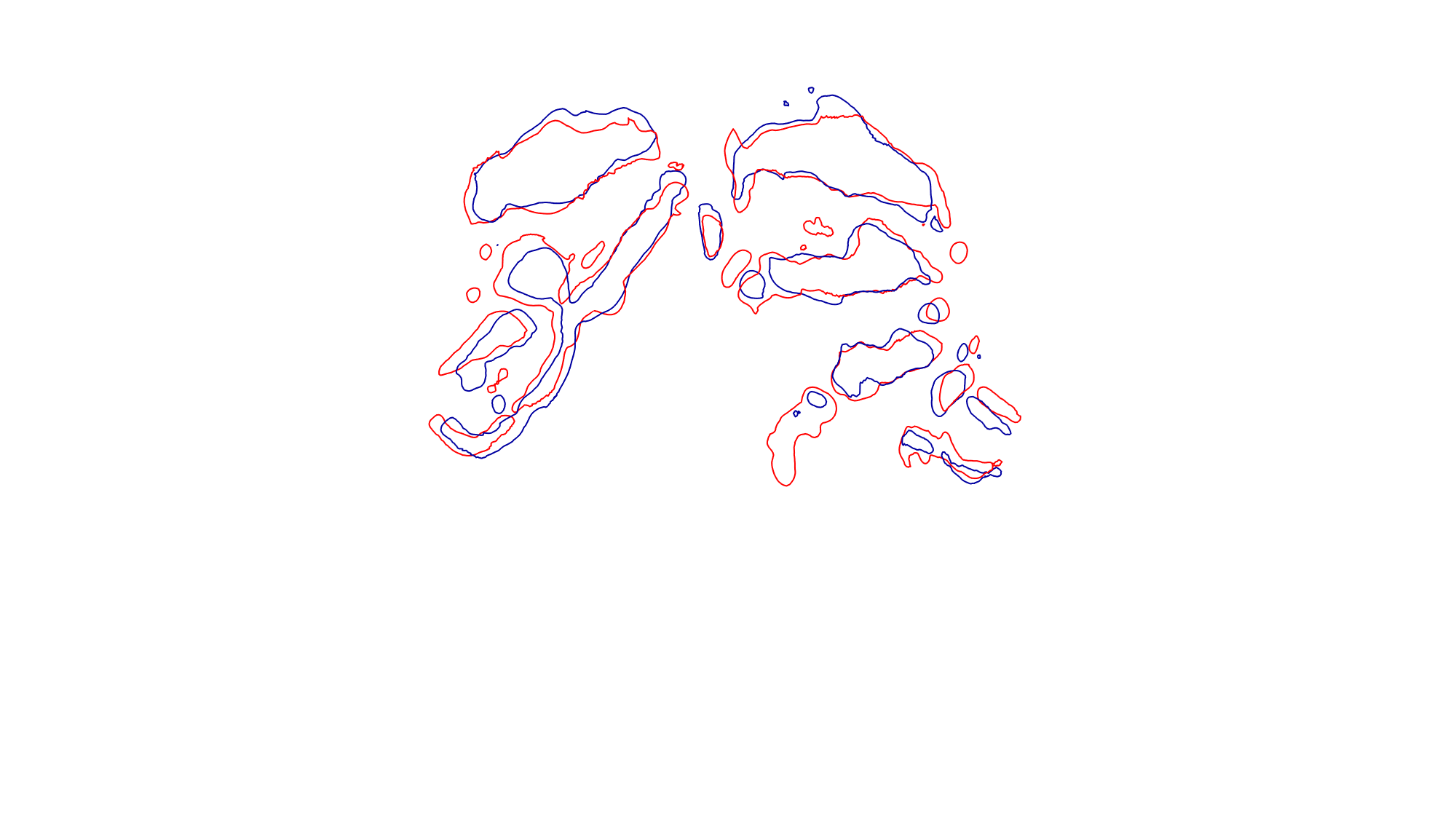 |
| **Nonex17 (15 years)** | **Nonex18 (13 years)** |
| 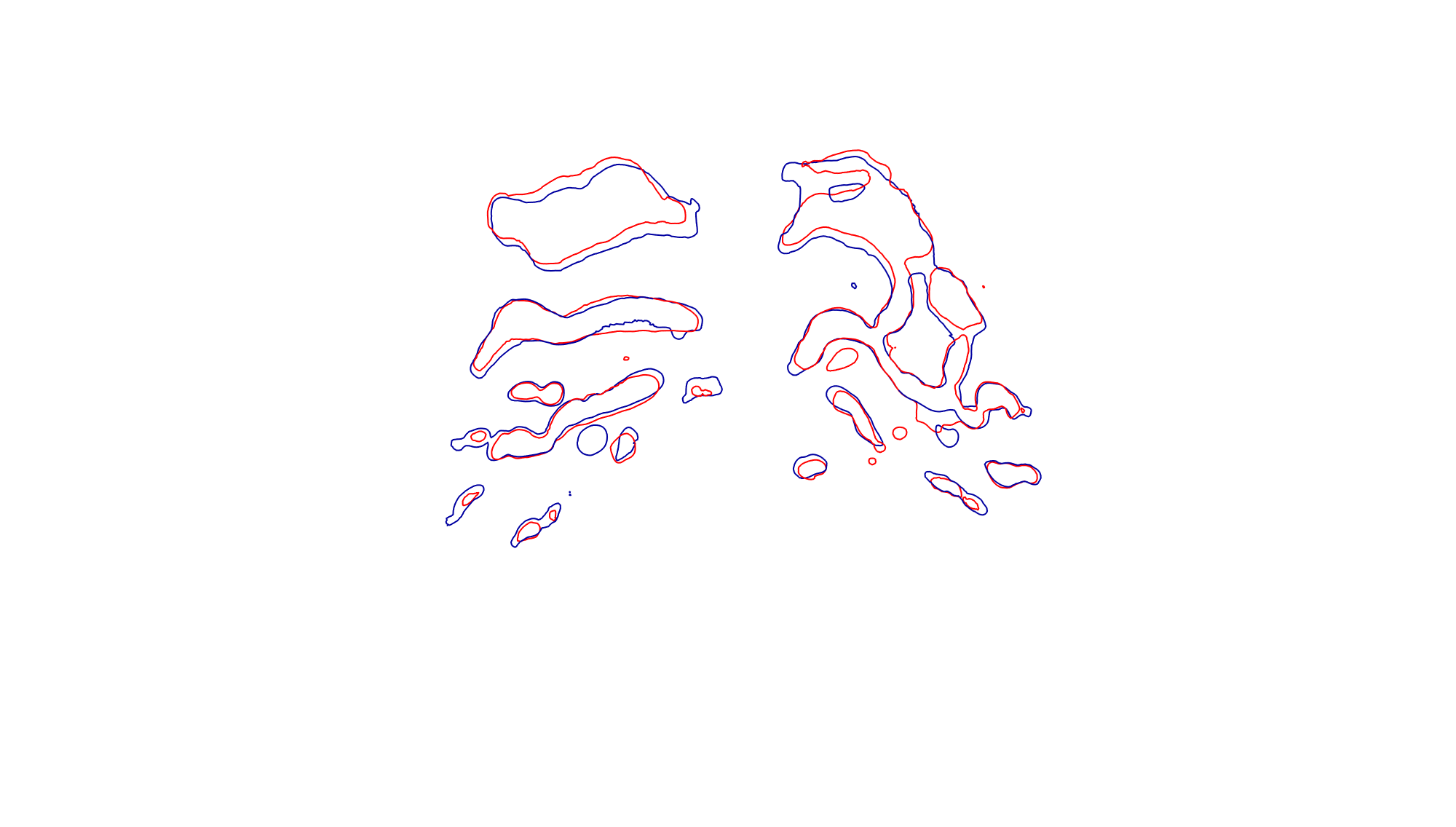 | 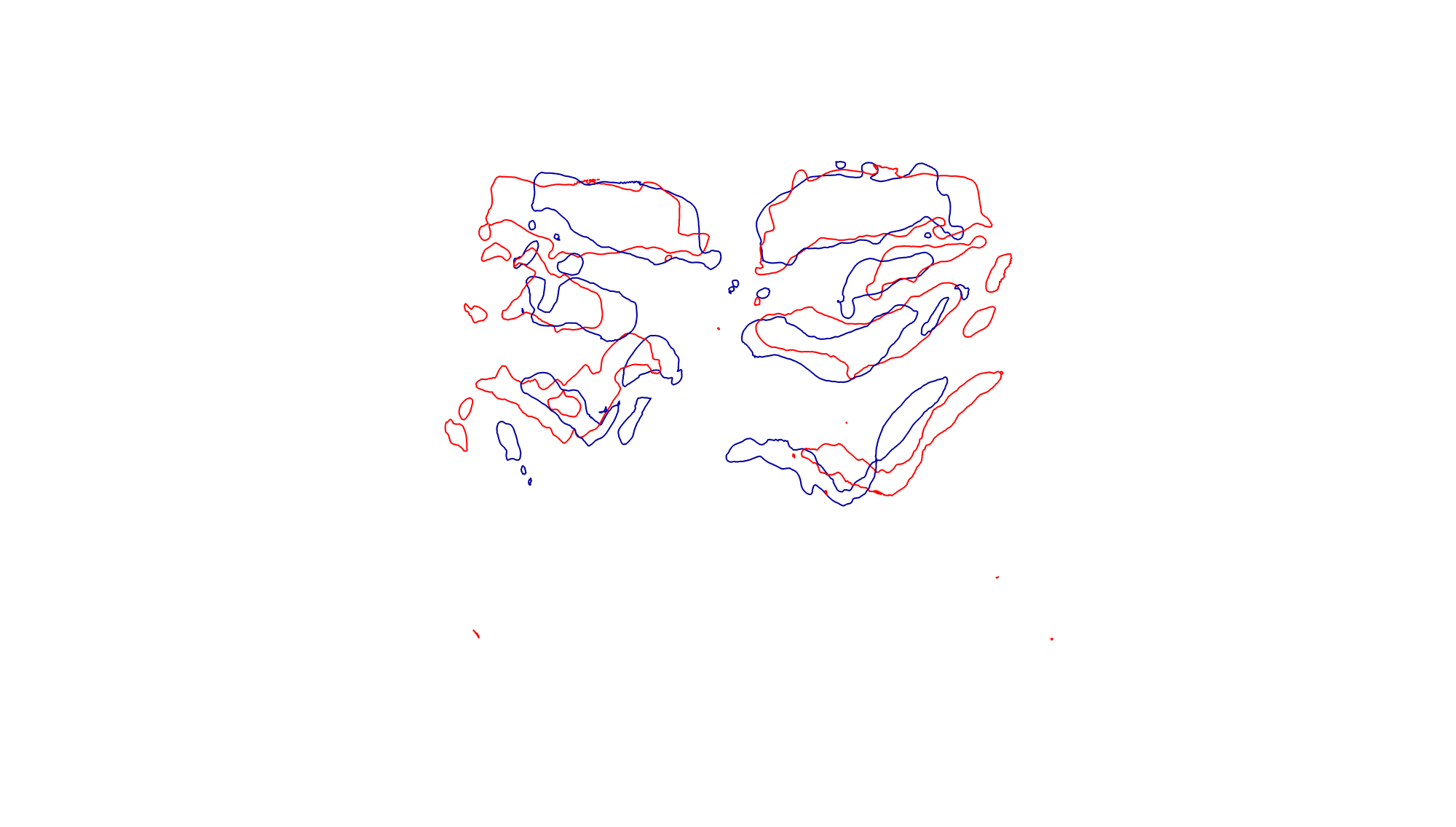 |
| **Nonex19 (12 years)** | **Nonex20 (21 years)** |
| 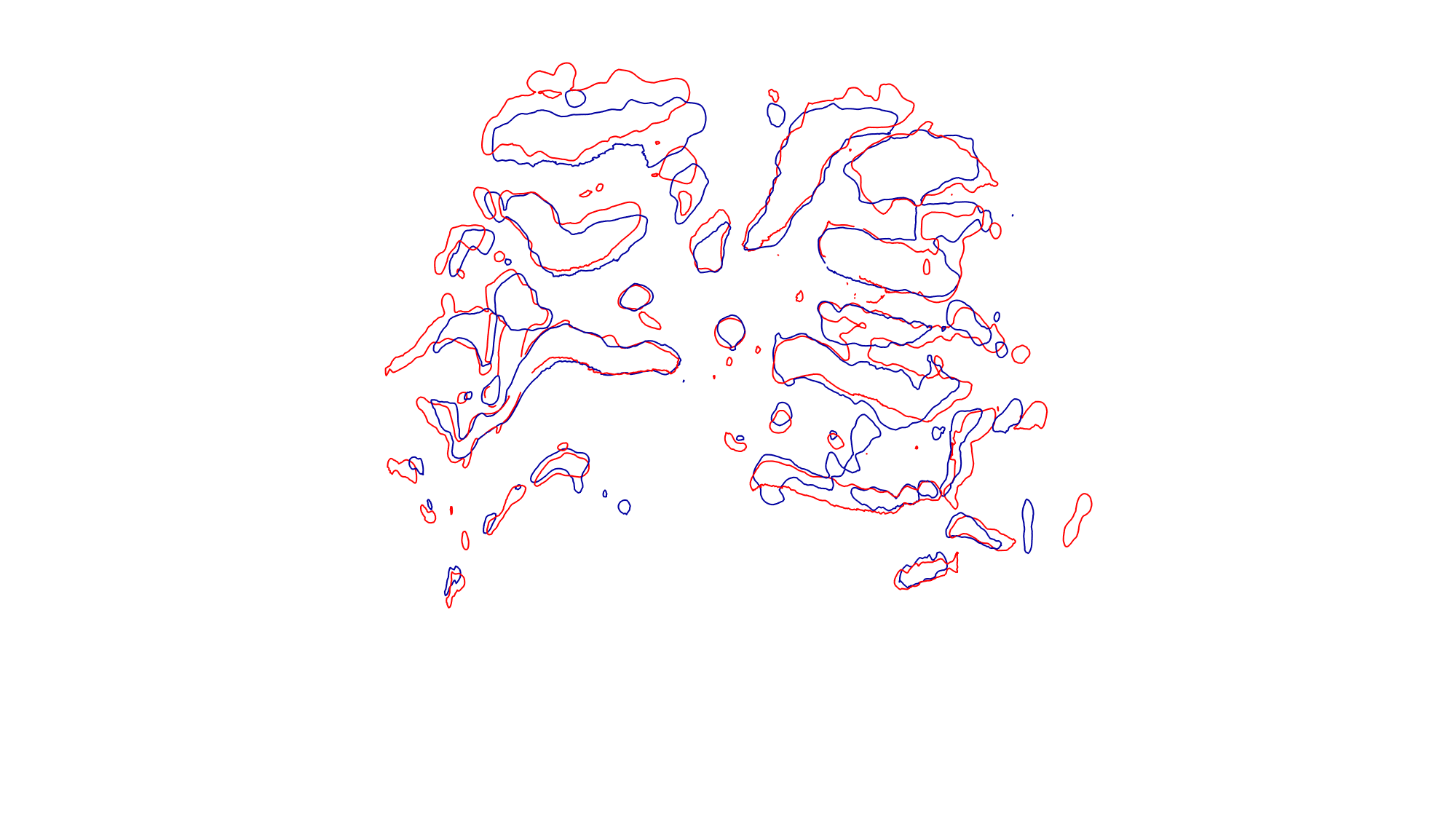 | 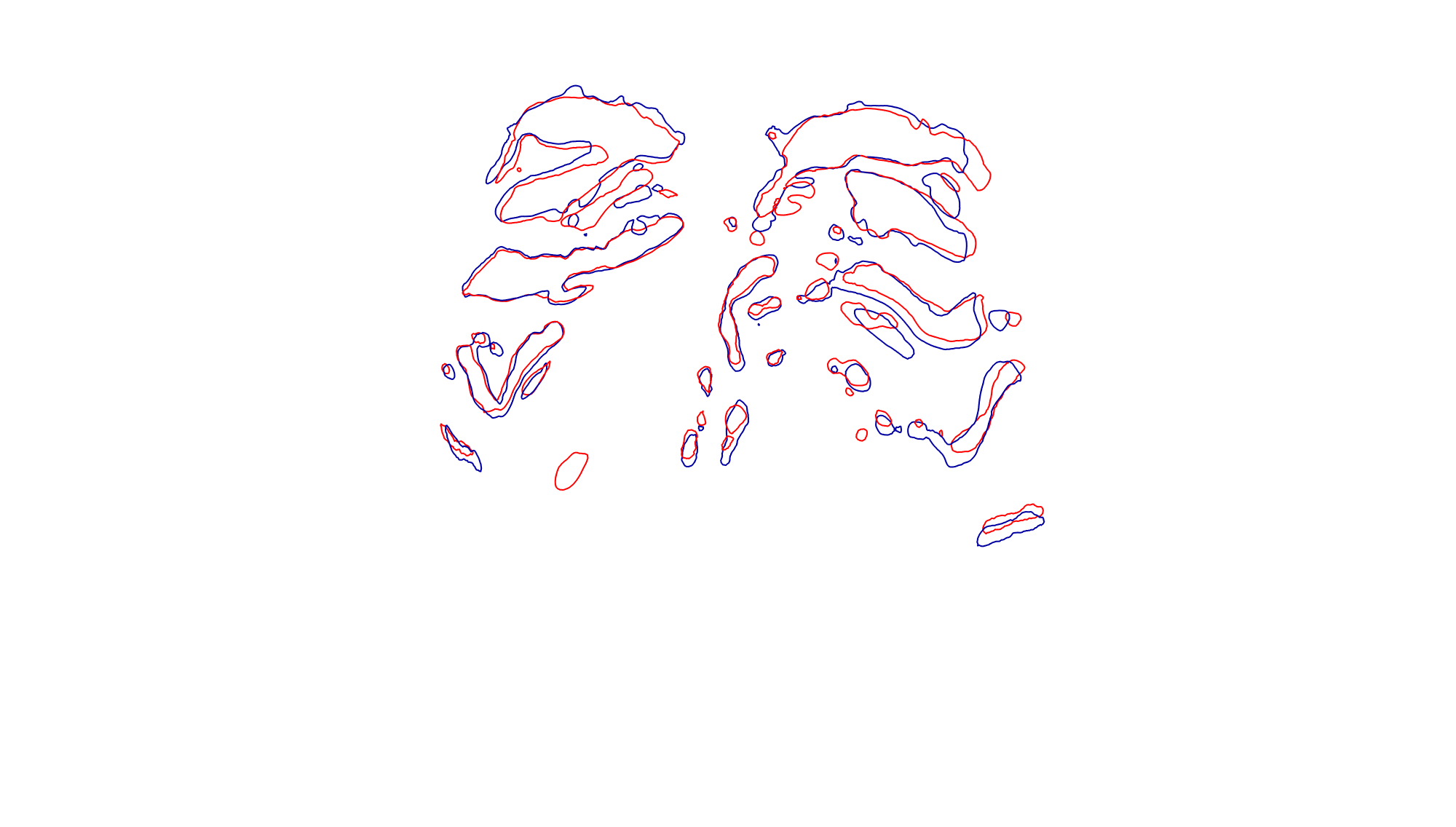 |
| **Nonex21 (11 years)** | **Nonex22 (10 years)** |
| 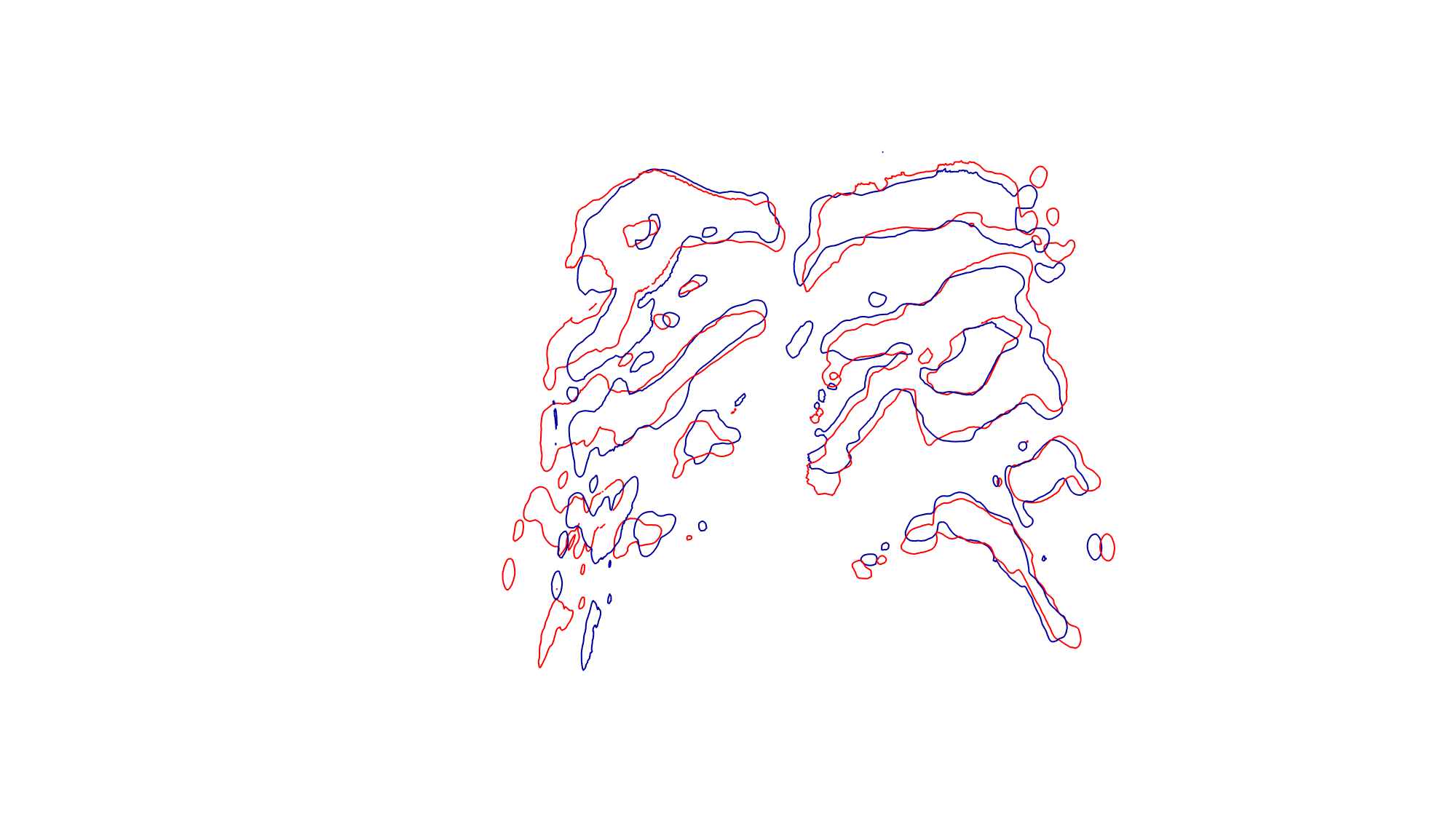 | 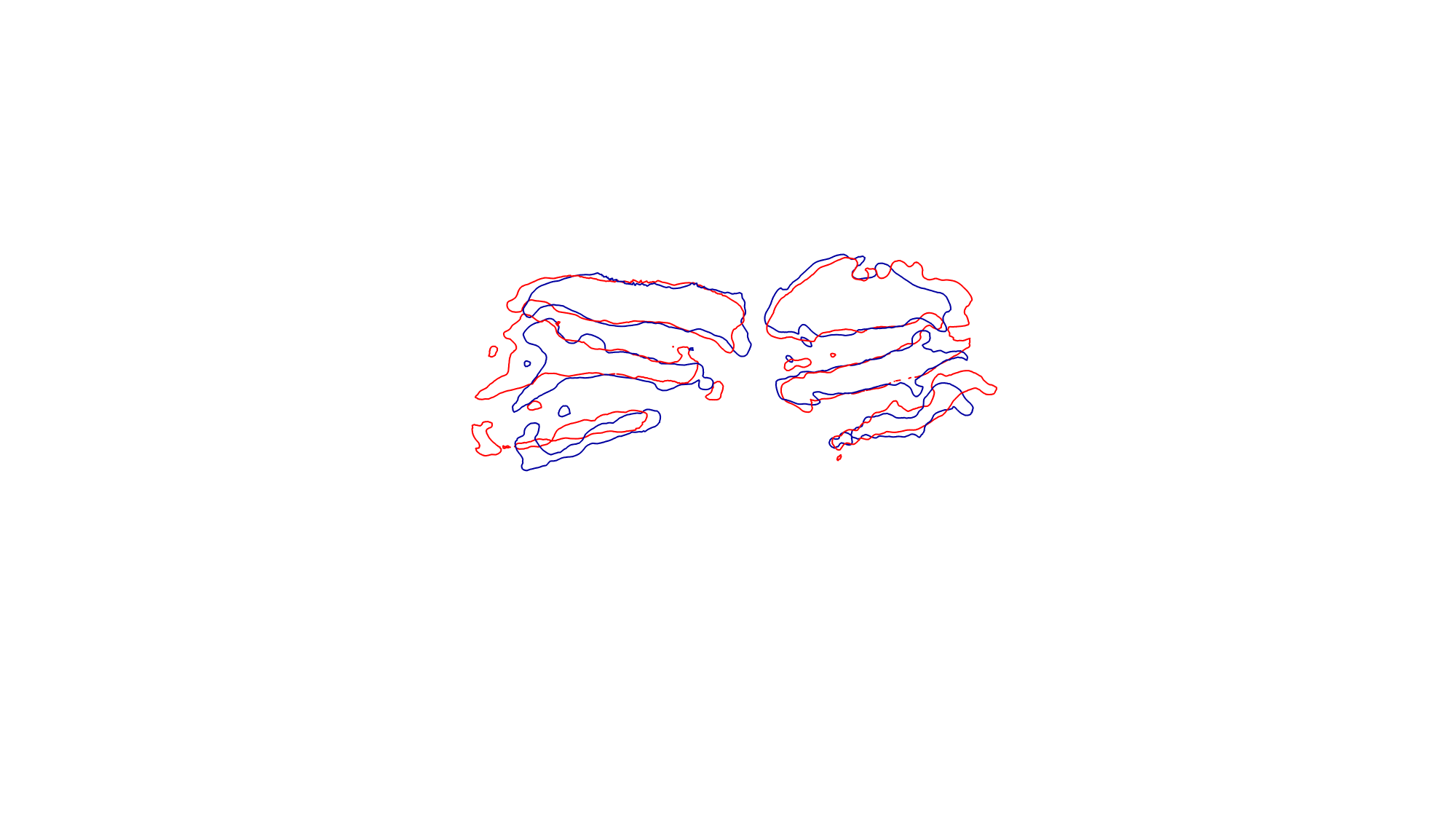 |
| **Nonex23 (11 years)** | **Nonex24 (11 years)** |
| 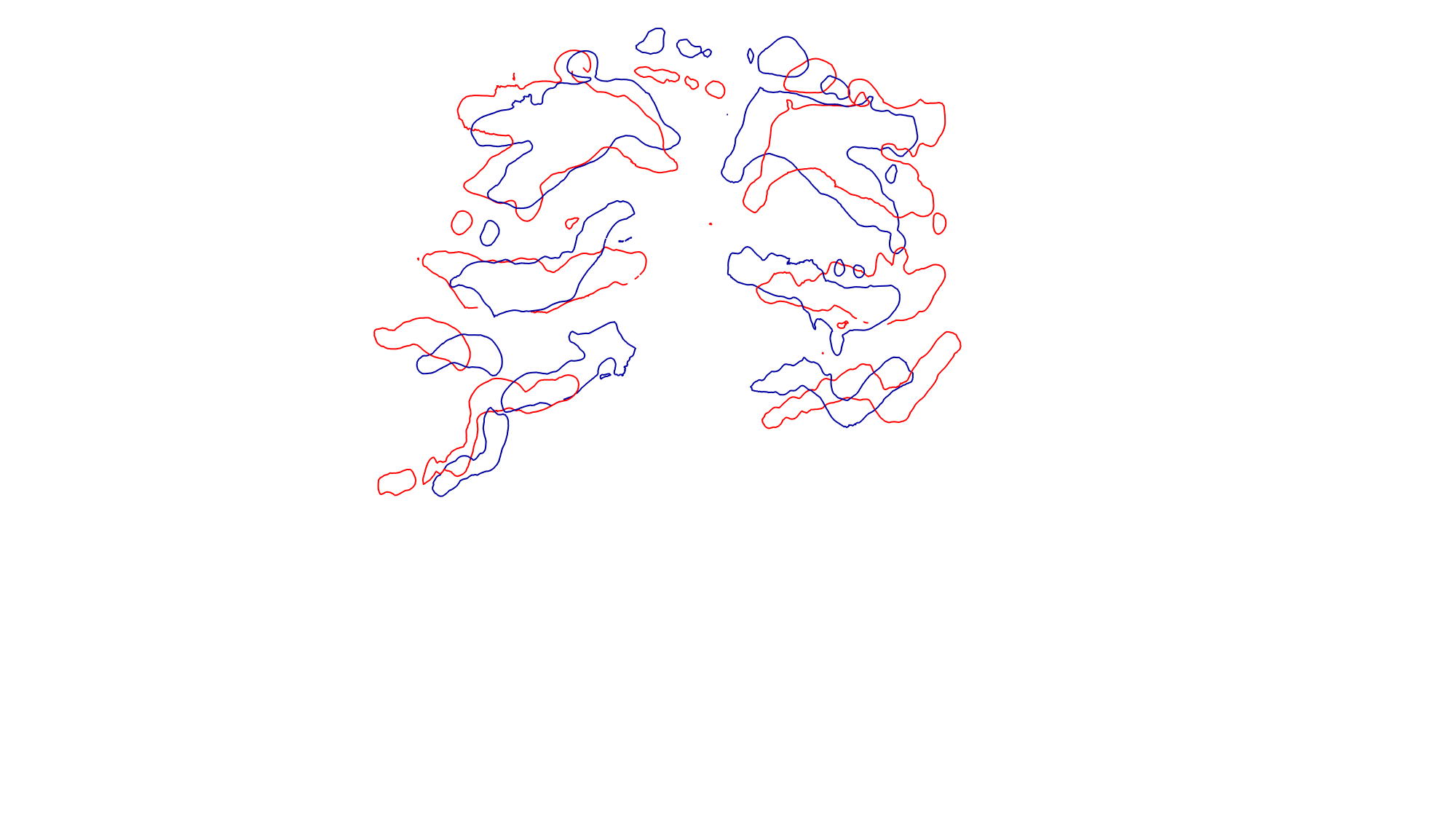 | 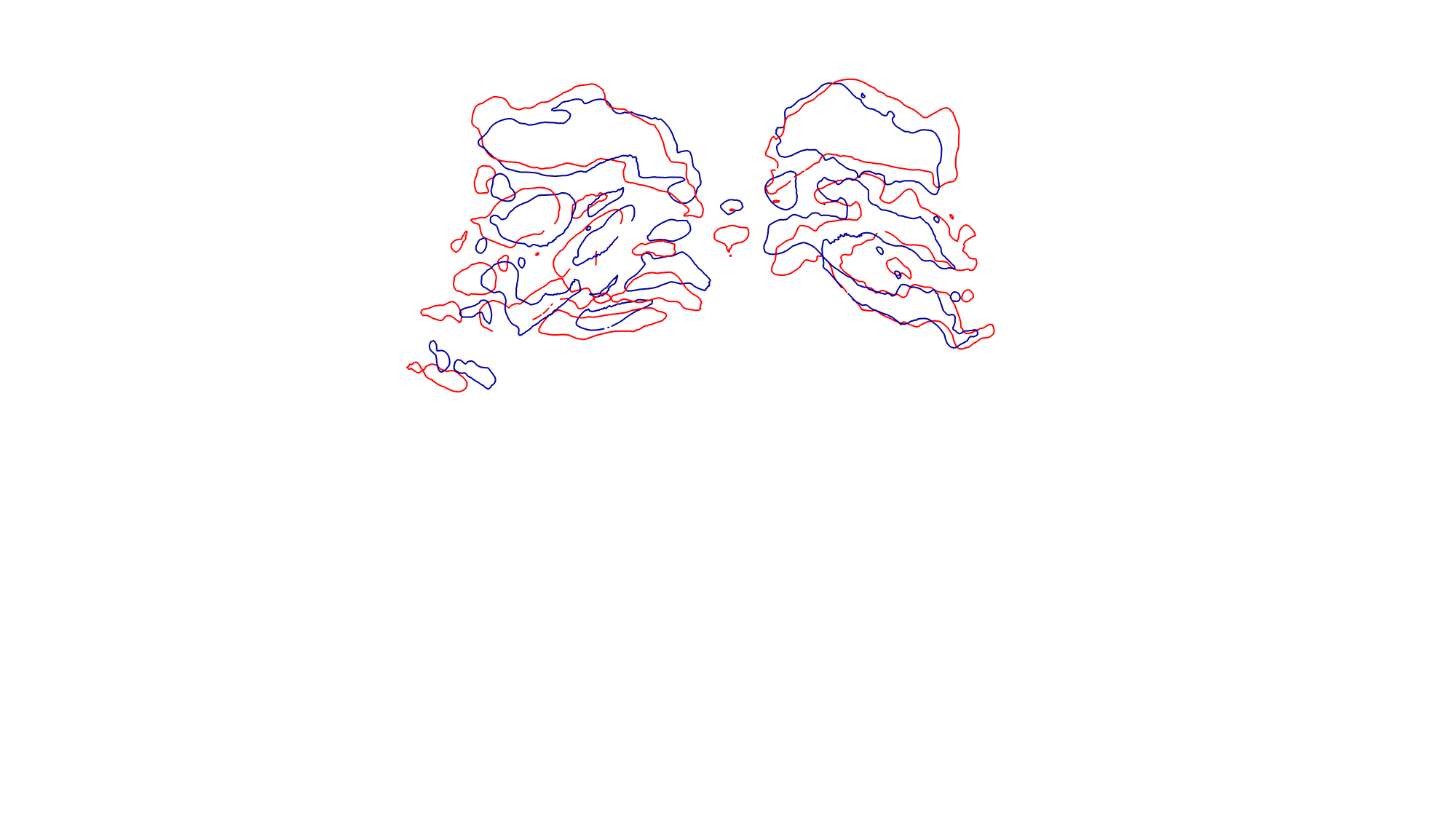 |
| **Nonex25 (19 years)** | **Nonex26 (16 years)** |
| 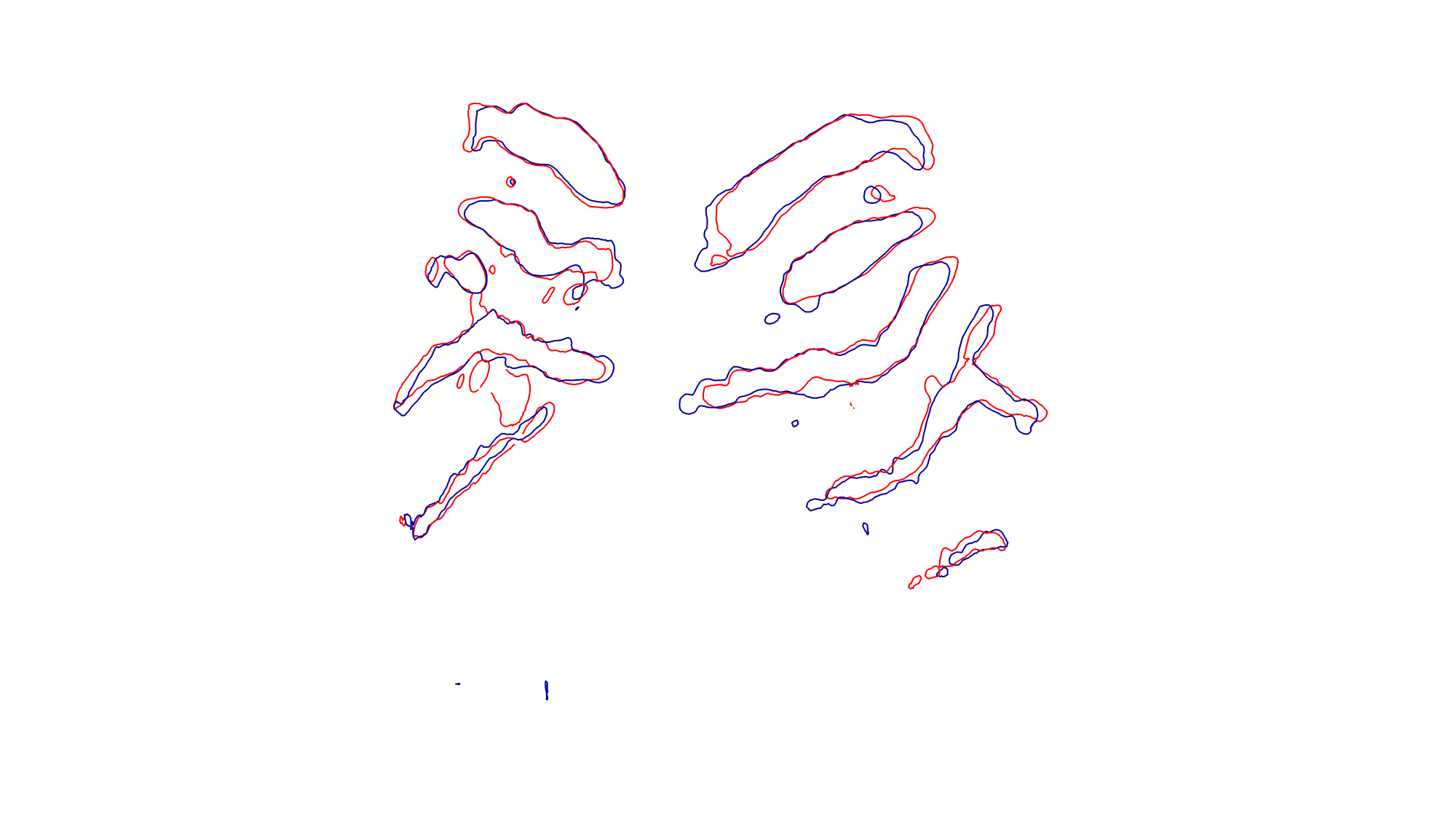 | 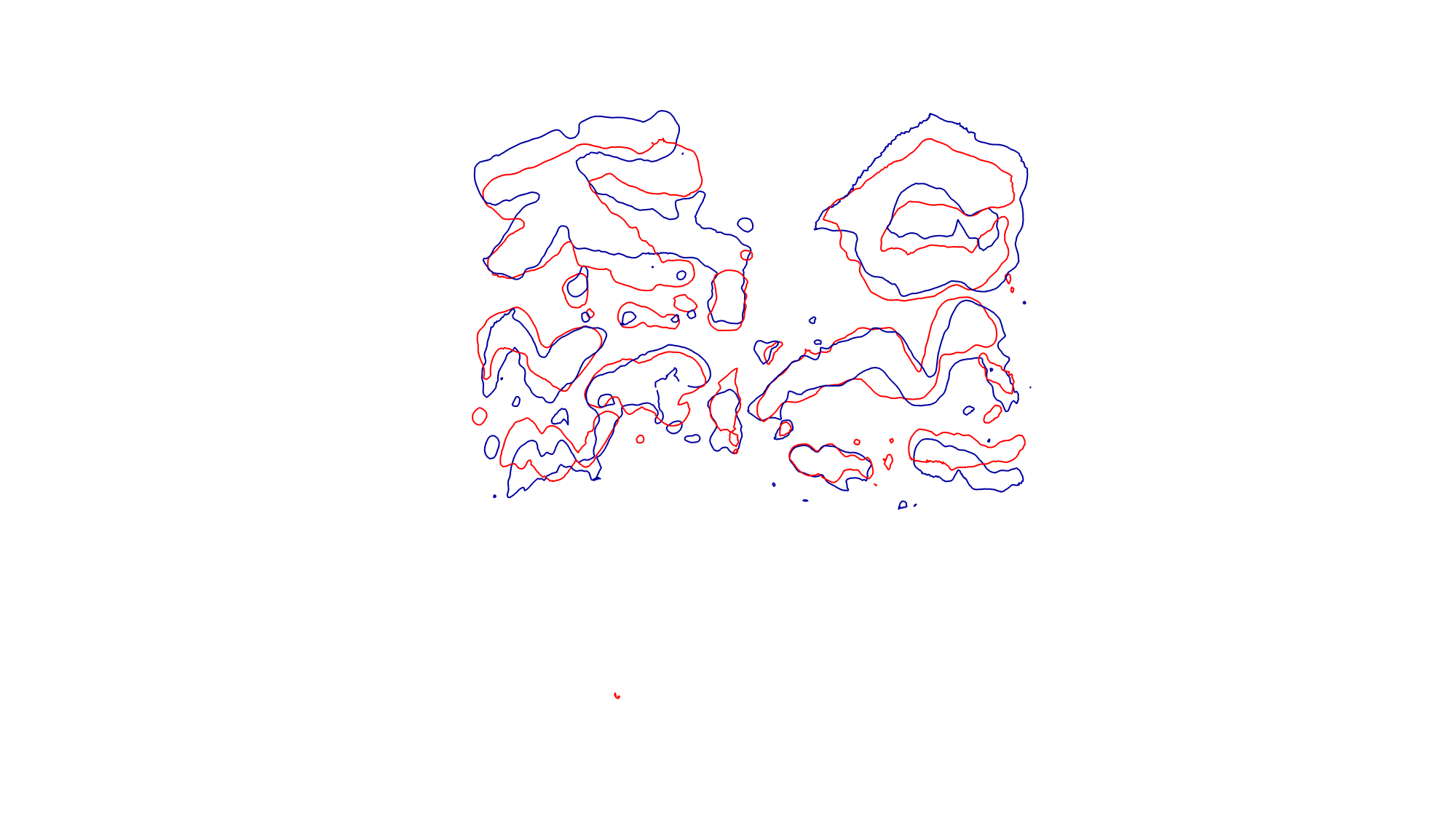 |
| **Nonex27 (11 years)** | **Nonex28 (13 years)** |
| 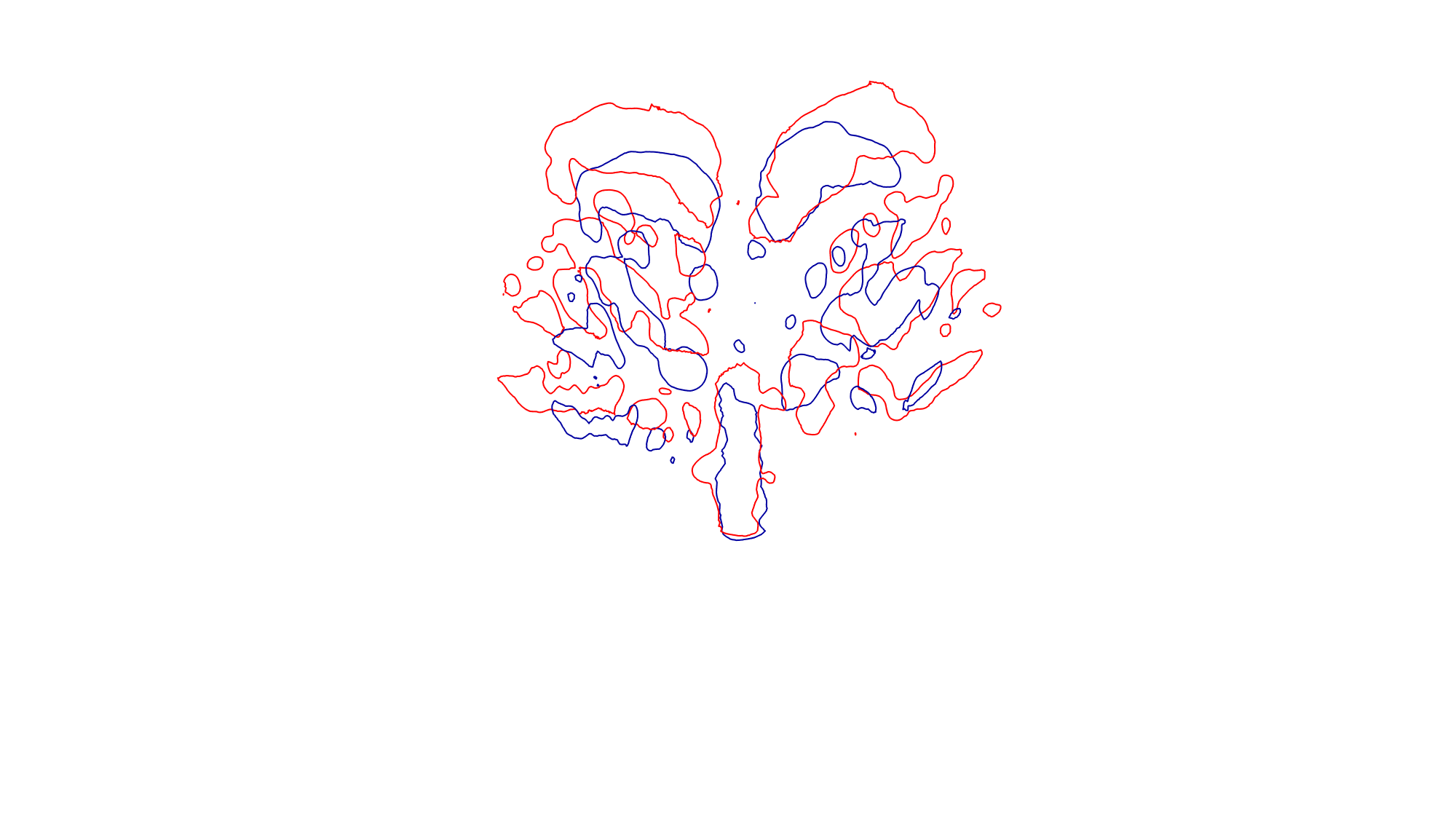 | 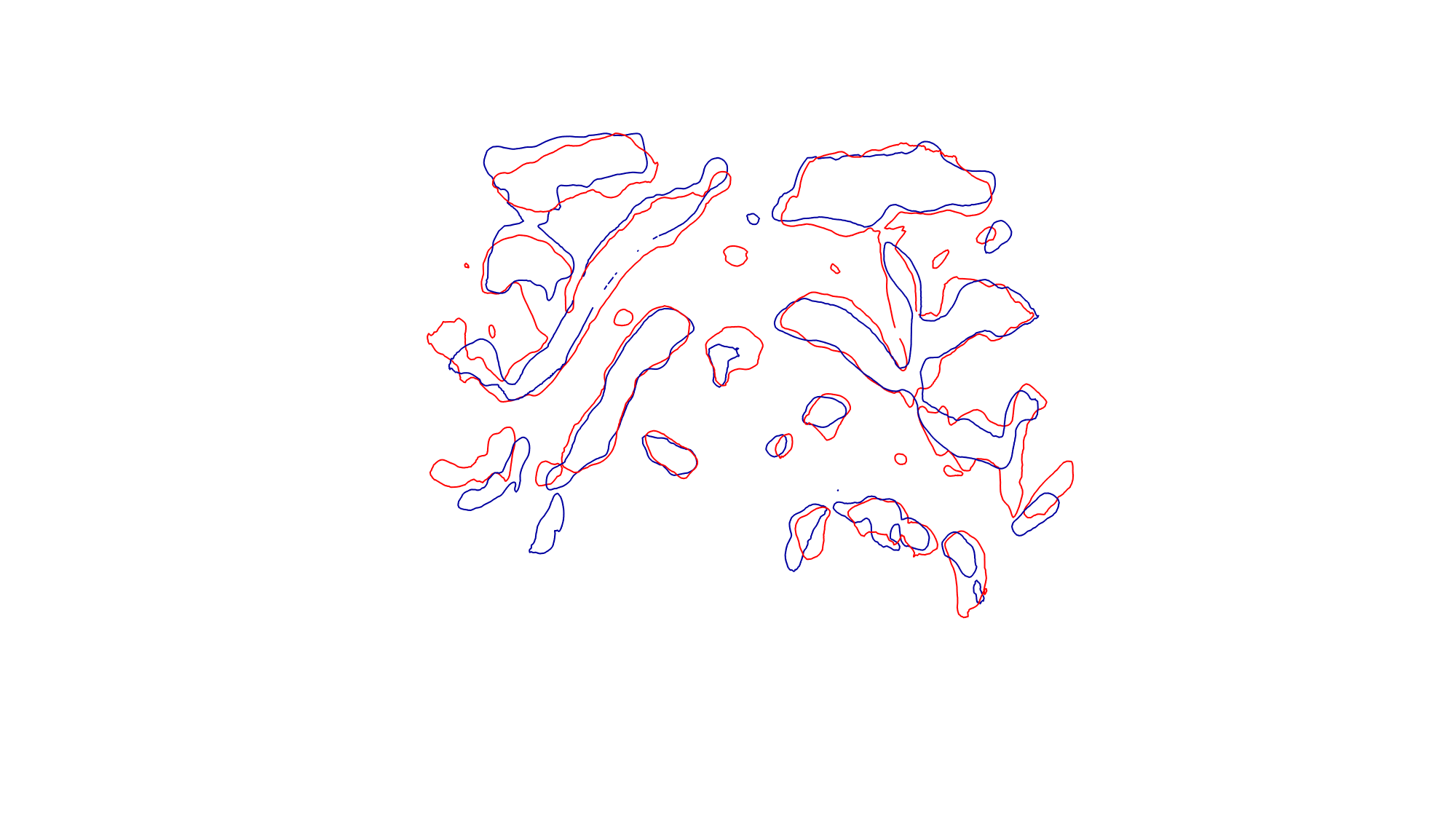 |
| **Nonex29 (29 years)** | **Nonex30 (14 years)** |
| 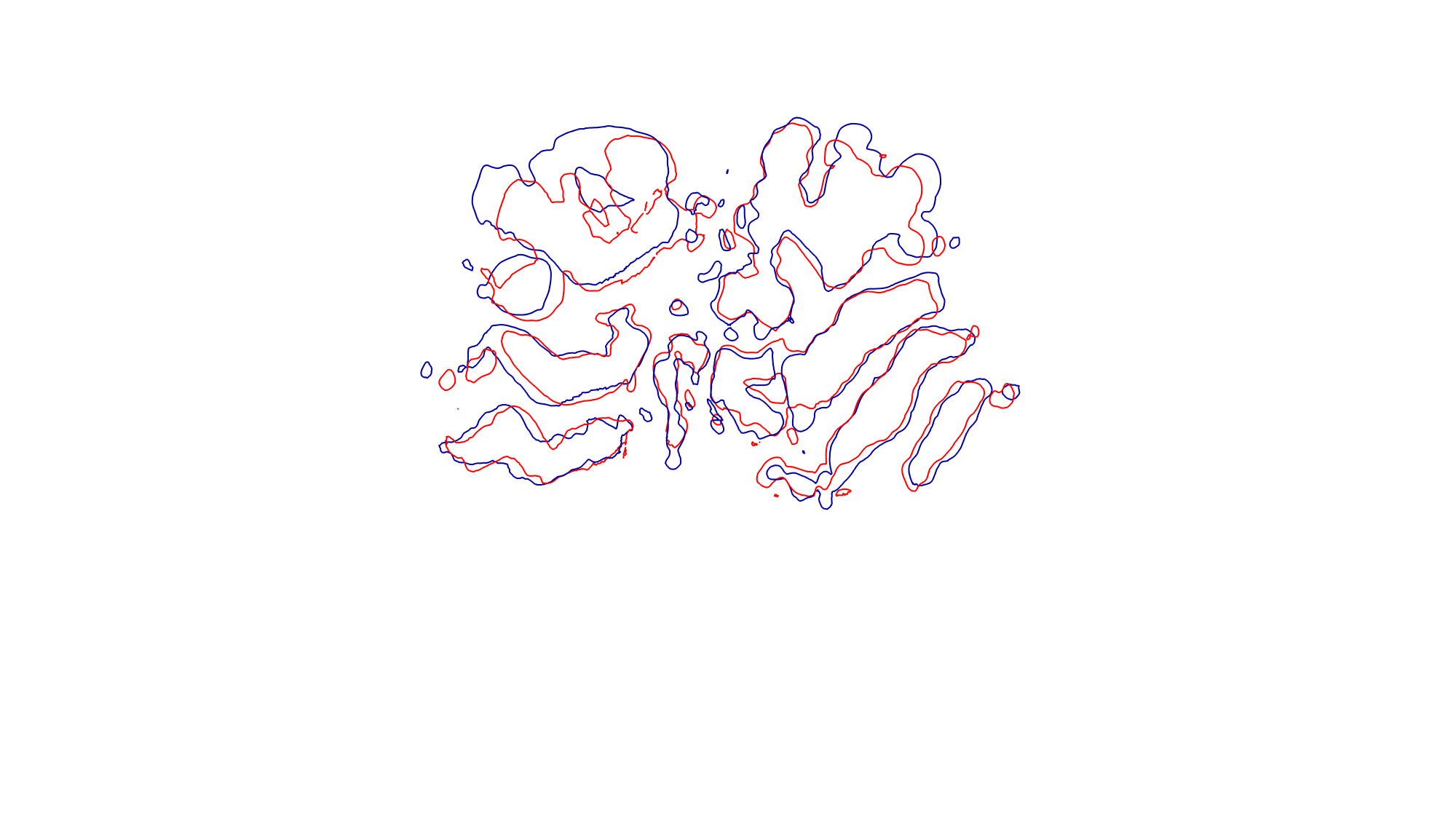 | 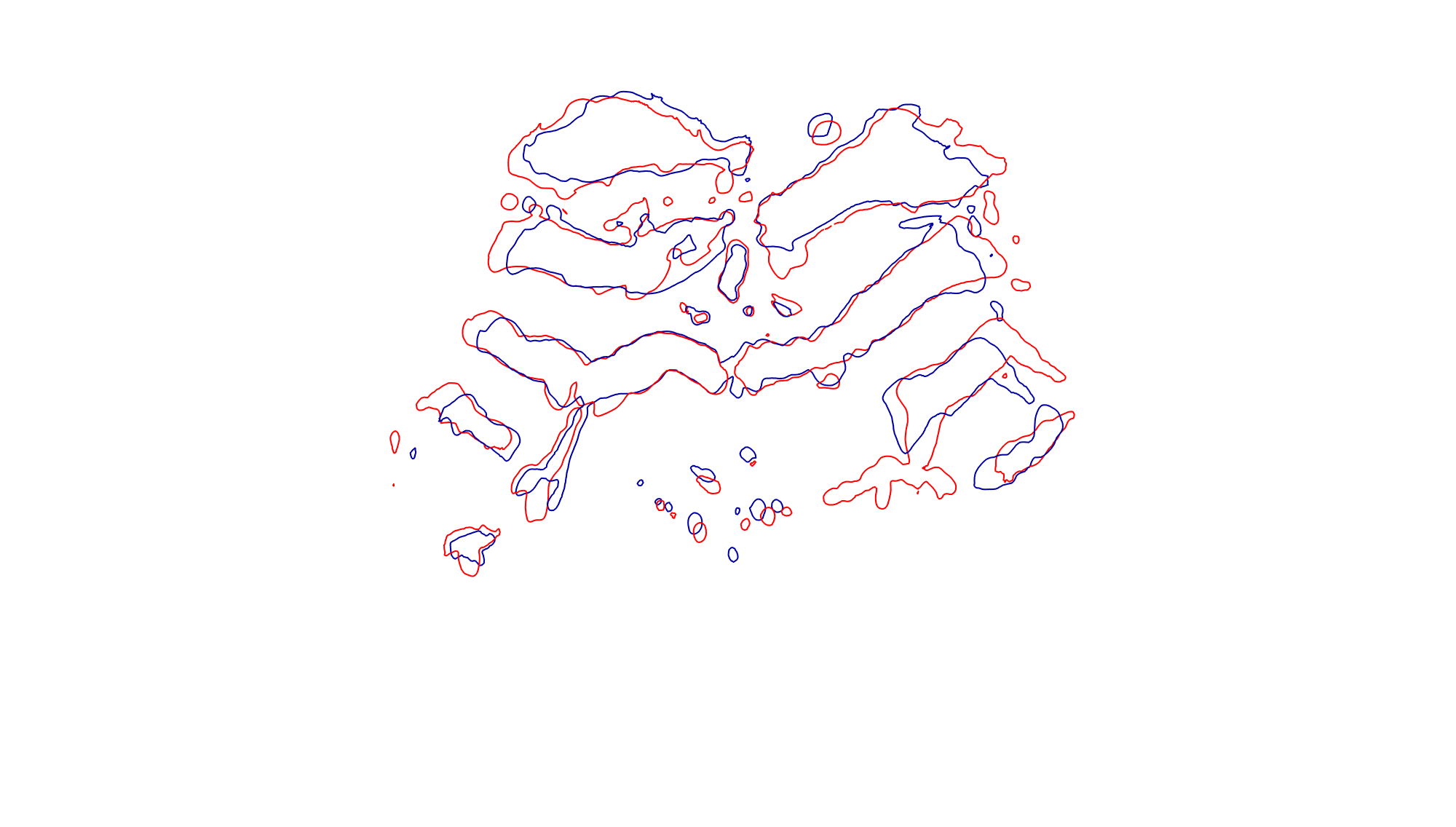 |
| **Nonex31 (23 years)** | **Nonex32 (15 years)** |
| 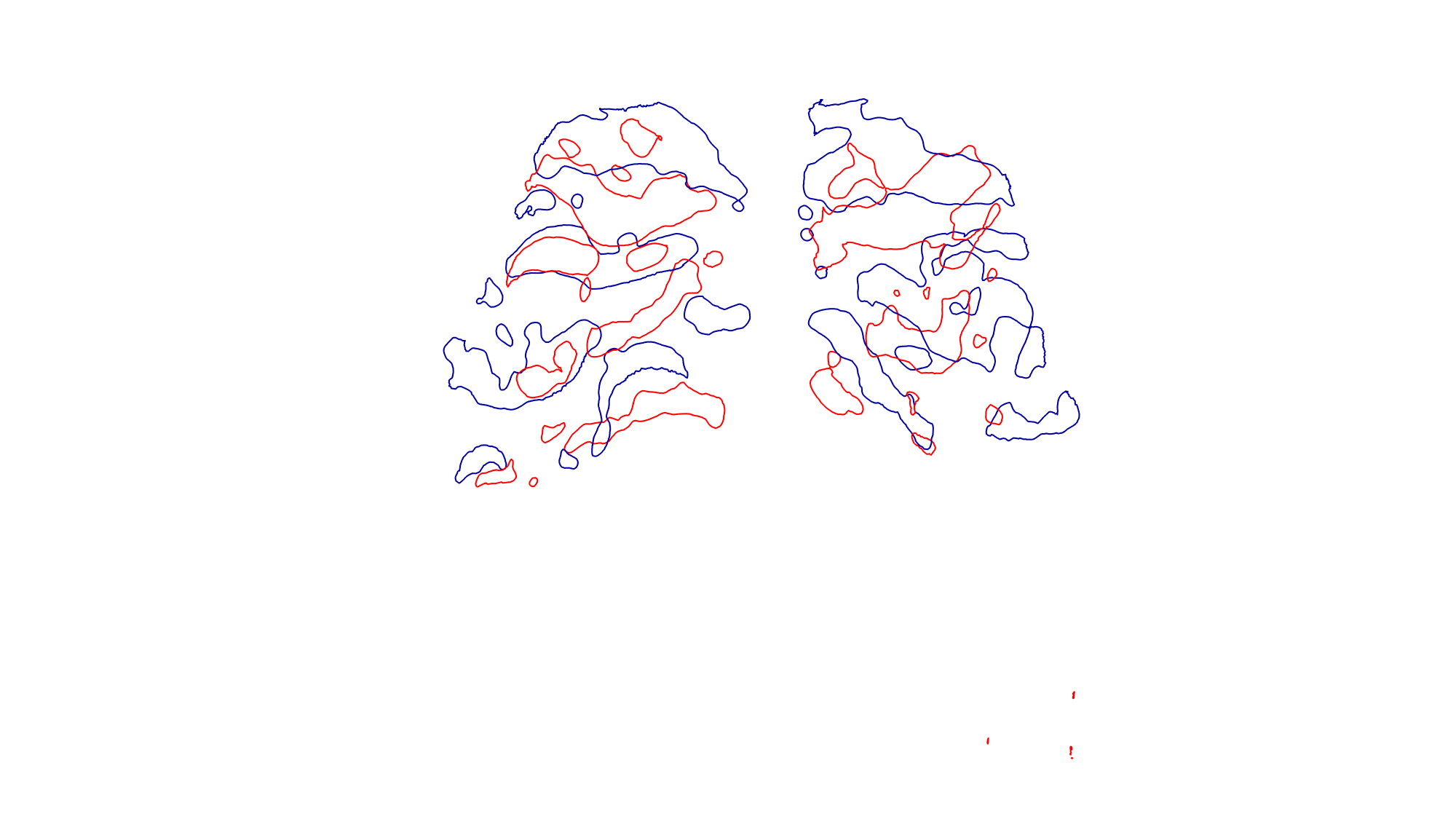 | 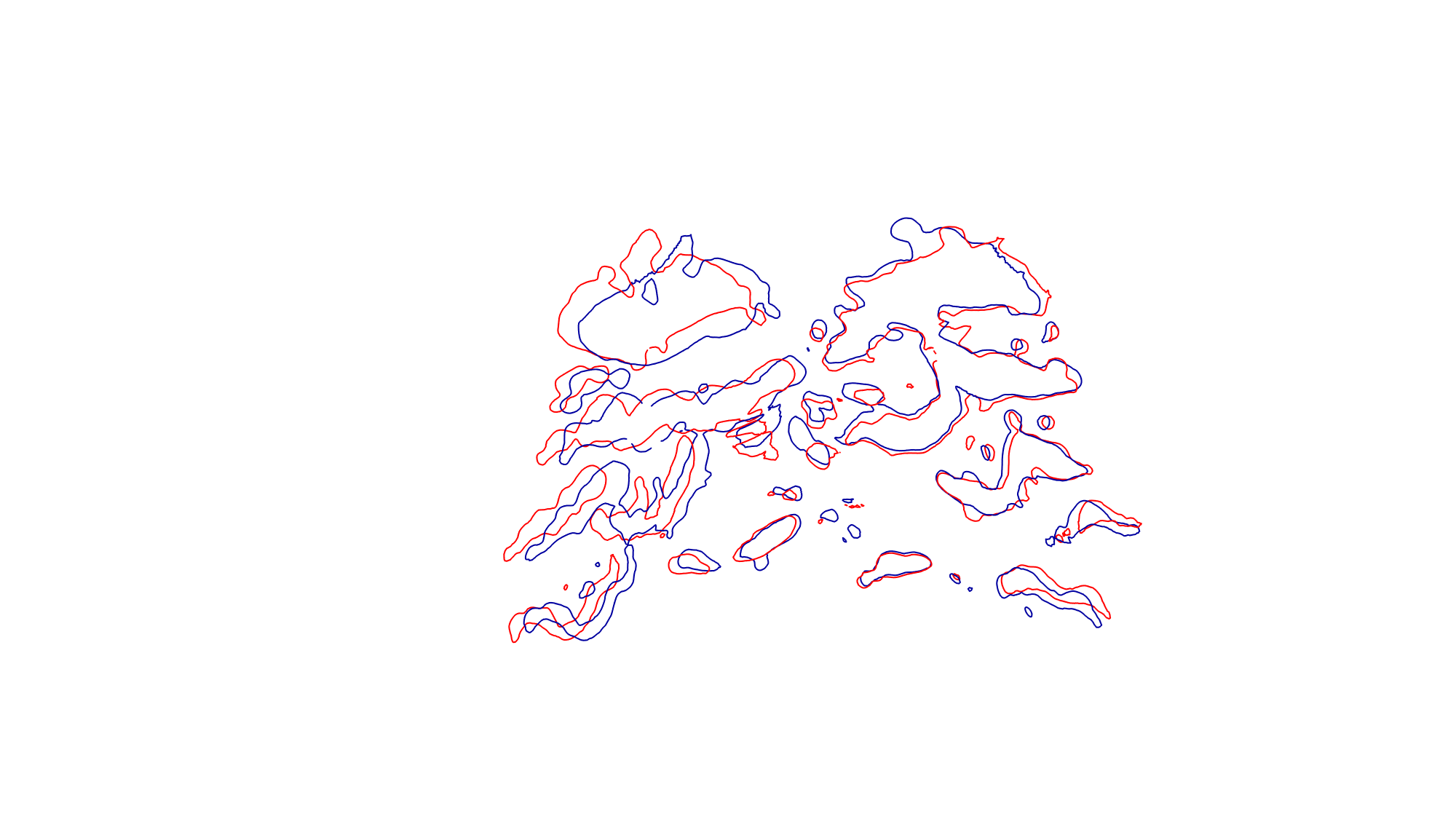 |
| **Nonex33 (27 years)** |  |
| 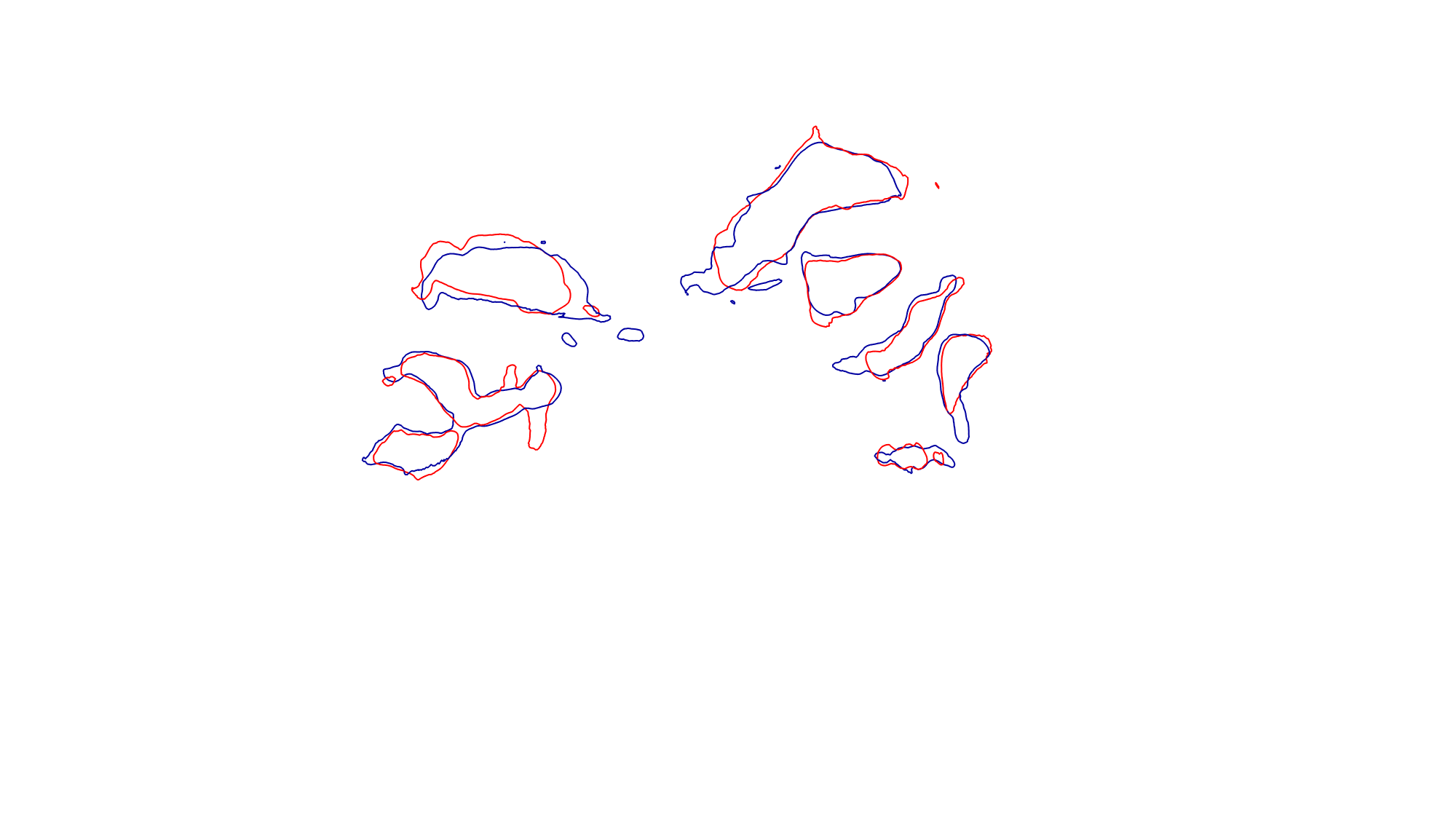 |  |
